# Cytological, Biochemical and Molecular Events of the Embryogenic State in Douglas-fir (*Pseudotsuga menziesii* [Mirb.])

**DOI:** 10.3389/fpls.2019.00118

**Published:** 2019-02-28

**Authors:** Florian Gautier, Philippe Label, Kateřina Eliášová, Jean-Charles Leplé, Václav Motyka, Nathalie Boizot, Zuzana Vondráková, Jiří Malbeck, Alena Trávníčková, Claire Le Metté, Marie-Claude Lesage-Descauses, Anne-Marie Lomenech, Jean-François Trontin, Guy Costa, Marie-Anne Lelu-Walter, Caroline Teyssier

**Affiliations:** ^1^BioForA, INRA, ONF, Orléans, France; ^2^PEIRENE, Sylva LIM, Université de Limoges, Limoges, France; ^3^UCA, INRA, UMR PIAF, Clermont-Ferrand, France; ^4^Institute of Experimental Botany of the Czech Academy of Sciences, Prague, Czechia; ^5^BIOGECO, INRA, University of Bordeaux, Cestas, France; ^6^Centre de Génomique Fonctionnelle, Plateforme Protéome, University of Bordeaux, Bordeaux, France; ^7^FCBA, Pôle Biotechnologie et Sylviculture Avancée, Cestas, France

**Keywords:** embryonal mass, non-embryogenic callus, histo-cytology, soluble carbohydrate, phytohormone, proteomic, network, transcriptomic

## Abstract

Somatic embryogenesis techniques have been developed for most coniferous species, but only using very juvenile material. To extend the techniques’ scope, better integrated understanding of the key biological, physiological and molecular characteristics of embryogenic state is required. Therefore, embryonal masses (EMs) and non-embryogenic calli (NECs) have been compared during proliferation at multiple levels. EMs and NECs originating from a single somatic embryo (isogenic lines) of each of three unrelated genotypes were used in the analyses, which included comparison of the lines’ anatomy by transmission light microscopy, transcriptomes by RNAseq Illumina sequencing, proteomes by free-gel analysis, contents of endogenous phytohormones (indole-3-acetic acid, cytokinins and ABA) by LC-MS analysis, and soluble sugar contents by HPLC. EMs were characterized by upregulation (relative to levels in NECs) of transcripts, proteins, transcription factors and active cytokinins associated with cell differentiation accompanied by histological, carbohydrate content and genetic markers of cell division. In contrast, NECs were characterized by upregulation (relative to levels in EMs) of transcripts, proteins and products associated with responses to stimuli (ABA, degradation forms of cytokinins, phenols), oxidative stress (reactive oxygen species) and carbohydrate storage (starch). Sub-Network Enrichment Analyses that highlighted functions and interactions of transcripts and proteins that significantly differed between EMs and NECs corroborated these findings. The study shows the utility of a novel approach involving integrated multi-scale transcriptomic, proteomic, biochemical, histological and anatomical analyses to obtain insights into molecular events associated with embryogenesis and more specifically to the embryogenic state of cell in Douglas-fir.

## Introduction

Douglas-fir [*Pseudotsuga menziesii* (Mirb) Franco] is a conifer native to the Pacific North-West of the United States and Canada, and one of the most important timber species globally. In Europe, it is frequently used for reforestation, partly to meet increasing demand for its wood, which has outstanding mechanical properties and durability. Commercial Douglas-fir plantations in France are constrained by limitation in capacities to produce seeds from the latest breeding tests. Although new seed orchards are being established to address this constraint, seed shortages in the near future cannot be excluded, especially if European demand increases. Vegetative propagation could provide a flexible, fast and efficient way to produce enough uniform genetically improved material for dissemination (Lelu-Walter et al., 2013). However, as in many conifers, early maturation resulting from a vegetative phase change in Douglas-fir hinders efficient, consistent and cost-effective mass cloning through conventional rooting of cuttings (Bastien et al., 2013). Somatic embryogenesis from immature seeds coupled with cryopreservation is a promising retroactive approach for clonal propagation of selected trees (Klimaszewska et al., 2016). However, despite several published studies on somatic embryogenesis in Douglas-fir (Durzan and Gupta, 1987; Pullman et al., 2009; Lelu-Walter et al., 2018; Reeves et al., 2018), and several relevant patents (Reeves et al., 2018 references therein), further information is required to realize its full potential.

Embryogenic cultures of conifers consist of EMs composed of early differentiated cells forming immature somatic embryo (SEs) that proliferate via cleavage polyembryony (von Aderkas et al., 1990). These SEs are typically bipolar structures with an apical embryonal head of meristematic cells tightly connected to a basal suspensor tissue composed of long, vacuolated cells. Cotyledonary SEs develop when EMs are exposed to maturation conditions (Lelu-Walter et al., 2018).

A characteristic cytological feature of somatic embryogenesis in Douglas-fir is interspersion of proliferating EMs with non-embryogenic cell clusters (Durzan and Gupta, 1987; Gautier et al., 2018; Reeves et al., 2018). The embryogenic state in plants, also referred to as embryogenic potential or embryogenicity, is defined in plants as the capability of cells to develop into rapidly proliferating early SEs resulting in establishment of embryo-generating culture (Bonga et al., 2010; Elhiti et al., 2013). It differs from regenerative capacity or maturation yield, which is the ability of propagated embryogenic lines to regenerate high-quality SEs after maturation (Miguel et al., 2016).

Conifers are considered highly recalcitrant to somatic embryogenesis from explants (e.g., shoot apices or needles) of selected trees in their adult vegetative and reproductive phases (Bonga et al., 2010; Trontin et al., 2016a). To date, the oldest coniferous material successfully used for the process has been shoot bud explants, of somatic origin, of up to 10-year-old *Picea glauca* trees (Klimaszewska et al., 2011). Strikingly, in this species, somatic embryogenesis proceeds from meristematic “nodules” that develop along needle primordia or embedded in non-embryogenic calli (NECs) formed on cut surfaces. In Douglas-fir, large polyembryogenic centers that occur in some embryogenic lines are cytologically similar to these nodules or meristemoids that typically develop during somatic embryogenesis in angiosperms (Gautier et al., 2018).

Thus, detailed characterization of embryogenic state, especially at molecular level, is required to complement macromorphological and cytological observations of proliferating structures (EMs, NECs, polyembryogenic centers, meristemoids and nodules) generated following somatic embryogenesis induction in conifers (Bonga et al., 2010; Rutledge et al., 2013; Klimaszewska et al., 2016; Miguel et al., 2016; Trontin et al., 2016b; Rutledge et al., 2017). A number of studies have already compared embryogenic and non-embryogenic tissues of several coniferous genera (*Araucaria, Larix, Picea*, and *Pinus* spp.) in terms of targeted gene expression (including miRNA), DNA methylation, and transcriptomic or proteomic profiling (reviewed in Miguel et al., 2016; Bravo et al., 2017; Klubicová et al., 2017). Putative marker genes of embryogenic state have been highlighted, including some that are reportedly involved in embryogenic induction in higher plants (Elhiti et al., 2013; Mahdavi-Darvari et al., 2015), particularly totipotency and commitment to somatic embryogenesis, e.g., *WUS*/*WOX2, LEC1*/*CHAP3A* and *BBM*/*SAP2C* (Miguel et al., 2016). However, most previous studies have focused on EMs and NECs of different genetic backgrounds or based on only one genotype. Usually NECs have been obtained from different vegetative tissues from those of the original explants used to initiate EMs. Moreover, there have been no previous integrated cytological, biochemical (especially phytohormonal), transcriptomic and proteomic comparisons.

Several previous studies have addressed the ability of cotyledonary SEs to undergo secondary somatic embryogenesis in various conifers, such as *Picea* spp., *Larix* spp., *Abies numidica, Pinus pinaster* and more recently Douglas-fir (Lelu-Walter et al., 2018 and references therein). In the last of these species, secondary EMs are typically induced in the presence of auxin and cytokinin from the root cap region of SEs, and under the same experimental conditions NECs are generally obtained from the hypocotyl region. Both types of culture obtained after secondary induction have the same genetic background, and thus provide ideal experimental systems to further characterize embryogenic state in a conifer species.

The objective in this study was to gain insights into molecular changes leading to the differentiation of immature SEs (EMs, embryogenic lines) rather than only calli (NECs). More knowledge of gene expression profiles associated with embryogenic state may help early detection of EMs and develop ways to overcome conifers’ recalcitrance to somatic embryogenesis and improve initiation rates of embryogenic lines. For this, we have compared isogenic EMs and NECs originating from a single SE, strictly initiated and then proliferated in the same media and under the same environmental conditions. We have applied a novel approach, involving integrated multi-scale analyses, combining genome-wide transcriptomic (RNAseq) and proteomic (free-gel/free-label) profiling followed by Sub-Network Enrichment Analyses (SNEA) to elucidate differences in functions and interactions of significant factors (transcripts and proteins) between EMs and NECs. Molecular data were complemented with morphological and histo-cytological, biological and biochemical analyses. We have also quantified endogenous phytohormones-auxin, ABA and CKs-with known importance in the control of cell proliferation and differentiation during somatic embryogenesis in conifers (Vondráková et al., 2018).

## Materials and Methods

### Plant Material

**FIGURE 1:**
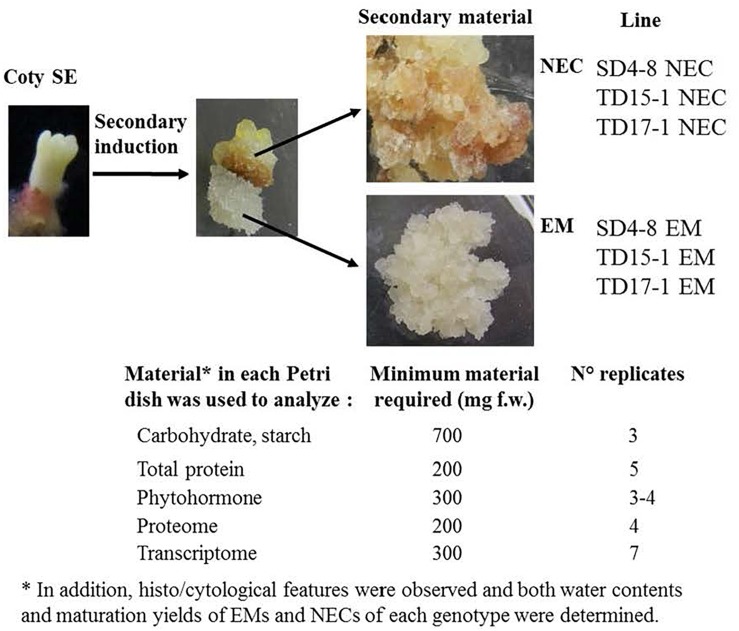
Origins and sampling scheme of the Douglas-fir plant material (EM and NEC) used for multi-scale characterization of the embryogenic state in three unrelated genotypes (SD4, TD15, and TD17). Cotyledonary SEs regenerated from primary SD4, TD15, and TD17 embryogenic lines were used as explants for induction of secondary somatic embryogenesis. Secondary EMs (SD4-8 EM, TD15-1 EM, TD17-1 EM) were obtained from the root cap region of SEs whereas NEC lines (SD4-8 NEC, TD15-1 NEC, TD17-1 NEC) were simultaneously obtained from the hypocotyl region of the same SEs during the same initiation experiment. All the EM and NEC material required (overall 1.7 g) for multi-scale comparison (soluble carbohydrate, starch, total protein, phytohormone, transcriptomic and proteomic), proliferated for 2 weeks and then samples were taken from the same Petri dishes, each treated as one biological replicate (3–7 replicates per type of analysis). Coty SE, cotyledonary somatic embryo; EM, embryonal mass; NEC, non-embryogenic callus.

Parental *Pseudotsuga menziesii* trees used in this study were obtained from either North Bend (trees designated 4440, 4455, and 4456) or Enumclaw (trees designated 4466, 4474, and 4477) in Washington State, United States. The following controlled crosses were performed at INRA, Orléans, France: 4474 × 4440, 4455 × 4466, and 4456 × 4477. Somatic embryogenesis was induced from immature zygotic embryos as described by Reeves et al. (2018) in 2011 to obtain primary (1^ry^) embryogenic line SD4 (4456 × 4477) and in 2012 to obtain 1^ry^ embryogenic lines TD15 (4474 × 4440) and TD17 (4455 × 4466). Secondary (2^ry^) somatic embryogenesis was then induced according to Lelu-Walter et al. (2018). Briefly, 6- to 11-week-old cotyledonary SEs (see maturation section) regenerated from 1^ry^ embryogenic lines, were isolated and cultured in 90 × 16 mm Petri dishes containing Glitz initiation medium supplemented with 4.5 μM 2.4-dichlorophenoxyacetic acid (2,4-D), 4.4 μM *N*^6^-benzyladenine (BA) and 0.087 M sucrose solidified with 4 g L^-1^ of gellan gum (Phytagel ^TM^, Sigma-Aldrich). The dishes were incubated in darkness at approximatively 23°C. Single SEs of each 1^ry^ embryogenic line gave rise simultaneously two morphologically and cytologically distinct types of cell lines (Figure 1): an embryogenic line (EM) developed from the embryonal root cap region, and NEC developed from the hypocotyl region. The 1^ry^ lines SD4, TD15 and TD17 generated 2^ry^ EMs respectively designated SD4-8 EM, TD15-1 EM and TD17-1 EM and NEC lines respectively designated, SD4-8 NEC, TD15-1 NEC and TD17-1 NEC. Each 2^ry^ line was subcultured as described below for proliferation. The pH of each medium was adjusted to 5.8 before autoclaving.

### Proliferation of EMs and NECs

Both the EMs and NECs were sub-cultured every 2 weeks on Glitz proliferation medium, supplemented with 4.5 μM 2,4-D, 2.2 μM BA and 0.087 M maltose solidified with 4 g L^-1^ of gellan gum (Reeves et al., 2018). When necessary, they were cultured as pieces of dispersed cell tissues (around 300 mg fresh weight, f.w.) onto a filter paper disk as described in Lelu-Walter et al. (2018). Briefly, proliferating plant material was collected and suspended in 4–5 mL of liquid proliferation medium, vigorously shaken to break up the tissue pieces into a fine suspension and poured as a thin layer onto a filter paper (Whatman No. 2, diameter 7 cm) in a Büchner funnel. EMs and NECs samples were collected after 2 weeks’ cultivation on proliferation medium. The material in each Petri dish, treated as a biological replicate, produced sufficient cellular biomass to analyze carbohydrates, starch, total proteins, phytohormones, the proteome and transcriptome. The number of biological replicates varied according to the type of analysis (Figure 1).

### Morphological and Microscopic Observations During the Proliferation Phase

EMs and NECs samples of all three investigated genotypes were collected after 10 days of multiplication for morphological and histo-cytological characterizations. Their morphology was documented using a SMZ 1500 stereomicroscope (Nikon, Tokyo, Japan). Fresh material was stained with either 0.4% (w/v) Trypan Blue (Sigma-Aldrich) as described by Vondráková et al. (2014) for cell structure observations or Lugol (iodine-potassium iodide) solution for observation of cells’ starch contents using a Jenaval transmission light microscope (Zeiss, Jena, Germany). For histological studies, EMs and NECs samples were fixed, dehydrated and infiltrated with paraffin as described by Lelu-Walter et al. (2018). Sections (12 μm thick) were stained with Alcian Blue and Nuclear Fast Red. Localization of phenolic compounds detected in vacuoles by Alcian Blue was also determined by staining with a 0.05% aqueous solution of Azur II and 1% Safranin in 50% ethanol (all dyes Sigma-Aldrich). Preparations were observed using a transmission light microscope. All images were captured using a DS-Fi3 camera (Nikon, Tokyo, Japan) and processed using NIS-Elements AR 5.0 software (Laboratory Imaging, Prague, Czechia).

### Maturation Conditions

Proliferating plant material (EMs and NECs) collected from filter papers was weighed, dispersed in liquid Glitz medium with no plant growth regulator and distributed on a filter paper disk placed on the surface of Glitz maturation medium supplemented with 0.2 M sucrose, 60 μM *cis- trans* ( ± )-ABA and 10 g L^-1^ gellan gum. The samples (initially 50 and 100 mg f.w. of EMs and NECs, respectively) were matured in darkness at approximatively 23°C. The number of cotyledonary SE produced after an 8-week period was counted in each of the Petri dishes and the maturation yield (number of SEs per g f.w.) was estimated. There were five Petri dishes for each type of material (technical replicates) and experiments were repeated two times (biological replicates), giving a total of 60 Petri dishes.

### Fresh Weight, Dry Weight, and Water Content

Samples of proliferating EMs and NECs (about 100 mg f.w.) collected from filter papers were immediately weighed to estimate their f.w. Their d.w. was determined after oven-drying at 70°C for 24 h (Teyssier et al., 2011) and their water content was calculated as g H_2_O g^-1^ d.w. (Dronne et al., 1997). These measurements were repeated 10 times.

### Phytohormones

Extracts of 3–4 biological replicates of each sample were used to analyze contents of the following phytohormones: ABA, ABA-GE, free auxin IAA and an array of CKs. The CK analysis included both isoprenoid forms—*cis*-zeatin (*cis*Z), *trans*-zeatin (*trans*Z), dihydrozeatin (DHZ), and *N*^6^-(Δ^2^-isopentenyl)adenine (iP)—and aromatic forms (*N*^6^-benzyladenine, BA) and their derivatives. Abbreviations of CKs follow Kaminek et al. (2000).

#### Pretreatment of Samples

Accurately weighed EMs and NECs samples (around 0.3 g f.w.) were placed in 2 mL Eppendorf tubes and mixed with 1.6 mL of modified Bieleski solution (methanol:formic acid:water, 15:1:4), supplemented with deuterated standards (Olchemim, Olomouc, Czechia) and ground using a MM 400 mixer mill (Retsch, Haan, Germany). The samples were left to extract overnight in a refrigerator at 4°C, then centrifuged and the pellets were re-suspended and re-extracted in the same volume (1.6 ml) of the same solution for 10 min in an DT 100 H ultrasonic bath (Bandelin electronic, Bandelin, Germany) at room temperature. After further centrifugation both supernatants were combined and loaded on a Strata C18-T SPE column (Phenomenex, Torrance, CA, United States) to remove non-polar compounds. The eluate was partly evaporated in an RVC Alpha rotary vacuum concentrator (Martin Christ, Osterode am Hartz, Germany) to approximately half the original volume and acidified by adding 1 mL of 1 M formic acid. The concentrated samples were loaded on an Oasis MCX SPE column (Waters, Milford, MA, United States) and further proceeded according to Dobrev and Kamínek (2002) with modifications below. Samples were cleaned with 2 mL of 1 M formic acid, then eluted in three fractions with 5 mL of 100% methanol (eluate containing ABA, ABA-GE, IAA), 5 mL of 2.5% (v/v) NH_4_OH (CK phosphates) and 5 mL of 2.5% (v/v) NH_4_OH in 60% methanol (non-phosphorylated CKs). The first and third fractions were evaporated in a rotary vacuum concentrator to dryness. The second fraction was evaporated to a 2 mL volume in a rotary vacuum concentrator, 100 μL of 2 M ammonium acetate (pH 10) and 10 μL of alkaline phosphatase solution were added and the samples were incubated at 37°C. After 1 h, the reaction was stopped by adding 20 μL of glacial acetic acid and the samples were loaded on a C18 SPE column. After cleaning with 2 mL of 5% methanol, the samples were eluted with 5 mL of 80% methanol and evaporated in the rotary vacuum concentrator to dryness.

#### LC-MS Analysis

Each dried extract was dissolved in 100 μL of 10% (v/v) acetonitrile, filtered through a nylon Micro-Spin 0.2 μm centrifugal filter (Grace, Columbia, MD, United States) and placed in a cooled sample stack. A portion of the extract (5 μL) was analyzed by a LC-MS system consisting of a Rheos 2200 HPLC pump (Flux Instruments, Basel, Switzerland) and HTS-Pal auto-sampler with cooled sample stack (CTC Analytics, Zwingen, Switzerland) coupled to a TSQ Quantum Ultra AM triple-quad high resolution mass spectrometer (Thermo Electron, San Jose, CA, United States) equipped with an electrospray interface. The HPLC column was tempered in a Delta Chrom CTC 100 Column oven (Watrex, Praha, Czechia).

The mass spectrometer was operated in multiple SRM (single reaction monitoring) mode (positive for CK analysis, negative for IAA, ABA and ABA-GE analysis) with acquisition of 2–4 transitions per compound. The most intense ion was used to quantify the analyte, the others to confirm its identity. The analytes were quantified using multilevel calibration curves with stable isotope labeled compounds used as internal standards. Each sample was analyzed twice.

#### CK Separation

Cytokinins were analyzed using an HPLC system with a Synergi 4 μm Hydro-RP 80 Å, 250 × 2.1 mm column (Phenomenex, Torrance, CA, United States) and a mobile phase consisting of a 30-min ternary gradient of water, acetonitrile and 0.01% of acetic acid (flow rate, 200 μL min^-1^). The proportion of acetonitrile was linearly increased from 8 to 50% and the acetic acid solution was maintained at 35% throughout each run, after which the column was washed with 90% acetonitrile.

#### IAA, ABA, and ABA-GE Separation

A Kinetex 2.6 μ C18 100 Å, 50 × 2.1 mm HPLC column (Phenomenex, Torrance, CA, United States) was used to analyze IAA, ABA and ABA-GE, using a 13-min water, acetonitrile and 1% (v/v) acetic acid ternary gradient as the mobile phase (flow rate, 200 μL min^-1^). The proportion of acetonitrile was linearly increased from 5 to 90%, and the proportion of acetic acid solution was kept at 10% during each run, after which the column was washed with 90% acetonitrile.

### Extraction of Soluble Proteins

Soluble proteins were extracted from five biological replicates of both EMs and NECs (200 mg f.w. of frozen material) with 1 mL of buffer containing 4 M urea, 0.1% v/v SDS, 0.1 M DTT, 80 mM Tris HCl (pH 6.8) and 10% (v/v) glycerol. Protein content was determined using the Bradford assay with bovine serum albumin as a standard. Results were expressed as soluble protein content in μg g^-1^ d.w.

### Quantification of Carbohydrates and Starch

Carbohydrates and starch were extracted following Bonhomme et al. (2010), with modifications. Briefly, EMs and NECs samples were lyophilized and ground into fine powder using a MM400 Retsch mixer mill. Each powder (20–40 mg d.w.) was extracted three times at 85°C in 1 mL of ethanol:water (80:20, v/v) containing mannitol as an internal standard (1 mg mL^-1^). Supernatants obtained after centrifugation were pooled, purified on activated charcoal (Merck) and poly(vinylpolypyrrolidone) (PVPP) to remove polyphenols and dried under vacuum in a Speedvac concentrator. The dry residues were suspended in 250 μl ultrapure water and centrifuged again before quantitative analyses, for which samples were injected in a HPLC system (VWR-Hitachi Chromaster) equipped with a 300 × 7.8 mm Rezex^TM^ RPM-Monosaccharide Pb+2 (8%) column (Phenomenex), and eluted with ultrapure H_2_O at a flow rate of 0.6 mL min^-1^. Eluates were quantitatively detected with an ELSD 85 detector (VWR Hitachi: drift tube temperature 85°C, N_2_ gas pressure 3.5 bars, gain 5, filter 6 s) and the peak areas were electronically integrated using OpenLAB CDS EZChrom (Agilent). Carbohydrates were identified by co-elution with standards, quantified from the calibration curves and expressed in mg carbohydrates per gram d.w. From the resulting pellets, we quantified starch content in glucose equivalents after hydrolysis with amyloglucosidase (Morel et al., 2014). Each sample was assayed in triplicate.

### Transcriptomic Analysis

RNA were extracted from seven biological replicates of both EMs and NECs (100 mg f.w. of frozen material). Samples were ground for 5 min with a mortar and pestle to a fine powder. Total RNA was extracted following Chang et al. (1993), and purified with an RNeasy Plant kit following the manufacturer’s recommended protocol, including in-column digestion of residual DNA using the RNase Free-DNase set (Qiagen). The extracted RNA’s quality was estimated using an Experion RNA analysis kit and automated electrophoresis system (Bio-Rad, France).

Five samples (1 of SD4-8 EM and TD15-1 EM, and 3 of TD17-1 EM) were removed after RNA extraction quality control made by the outsourced company (GATC Biotech, Germany) and before sequencing, resulting in a total of 37 samples. Libraries were constructed and sequenced by GATC after polyA selection. Strand specific cDNA libraries were synthesized and all samples were paired-end sequenced using an Illumina HiSeq 2500 system and the manufacturer’s recommended procedures. Raw sequencing reads (between 40,000,000 and 50,000,000 reads per library) are available through SRA BioProject accession N° PRJNA491234^1^. Primer-adapter removal, quality filtering, ambiguous base trimming, polyT and polyA removal and decontamination were performed following standard procedures, resulting in removal of 0.89% of short-reads, leaving 5,033,238,314 short-reads, 60–126 bp-long, in total from the 37 samples.

The short-reads were mapped using reference *Pseudotsuga menziesii* sequences obtained from the V1 transcriptome database (UC-Davis, United States)^2^. Transcripts were annotated using Blast2GO (Götz et al., 2008). GSEA (Gene Set Enrichment Analysis) was performed using GO database (Carlson, 2017) and TopGO package (Adrian and Rahnenfuhrer, 2016). Over-abundance of GO terms in differentially expressed transcript sets was detected with a classification index generated using elim and weight algorithms in combination with three statistical tests (Fisher, KS, T) according to the TopGO user manual, yielding multi-ranking *p*-values. Graphics were produced with ggplot2 (Wickham, 2009) and Tidyverse (Wickham, 2017) R libraries. Short-reads were mapped using the bwa-sampe algorithm (Li and Durbin, 2009), allowing no more than 1 nucleotide mismatch per mapped read (estimated from the sequencing error rate). Fifty-eight percent of Illumina short-reads mapped onto the Douglas-fir reference transcriptome.

### Proteomic and nLC-MS/MS Analyses

Four biological replicates per sample were subjected to proteomic and nLC-MS/MS analyses following Gautier et al. (2018). Briefly, each protein sample was loaded onto SDS-PAGE gel and digested with trypsin. The eluted peptide mixture was analyzed using an Ultimate 3000 nanoLC system (Dionex, Amsterdam, Netherlands) equipped with a C18 PepMap^TM^ trap column (LC Packings) coupled to an Electrospray Q-Exactive quadrupole Orbitrap mass spectrometer (Thermo Fisher Scientific, San Jose, CA, United States). Proteins were identified by SEQUEST searches implemented via Proteome Discoverer 1.4 (Thermo Fisher Scientific Inc.) against a *Pseudotsuga menziesii* v1 transcriptome proteome database from PineRefSeq (54,830 HQgenes complete proteins^3^). The mass spectrometry proteomics data have been deposited to the ProteomeXchange Consortium via the PRIDE (Vizcaíno et al., 2016) partner repository with the dataset identifier PXD011176.

Significantly differentially expressed proteins were functionally classified using Gene Ontology Consortium (GO) codes^4^, drawn from the Douglas-fir and *Arabidopsis*^5^ (TAIR) databases to complete the annotation.

### Functional Characterization of Proteins and Gene Ontology Analysis

Changes in protein expression were calculated in comparison with corresponding control based on the cumulative intensity in each peptide. All sequences have been mapped with GO terms against *Arabidopsis thaliana* database^5^ to complete functional annotation. The proteins were then classified based on their biological functions using Web Gene Ontology Annotation Plot software at level 2 for biological process (BP) (Panther)^6^ (Mi et al., 2013). A binomial test and Bonferroni’s correction were performed with the Panther software to determine significant GO identifiers occurring more often in a group.

### Sub-network Enrichment Analysis

SNEA of selected genes was performed using Plant Pathway Studio^®^ version 12 (Elsevier B.V.), with a threshold *p*-value of 0.05 and minimum overlap of two genes per significant Sub-Network. Only proteins/chemicals regulating diseases or cell processes neighbors in the Pathway Studio^®^ database were searched. A pathway was built by combining the significant Sub-Networks found.

### Statistical Analyses

R version 3.3.2 (R Development Core Team, 2011) was used for all statistical analyses. Observed differences between EMs and NECs in maturation yield or contents of water, starch, carbohydrate, phytohormones or protein were assessed by one-way analysis of variance (ANOVA) and multiple comparisons of means with Tukey contrasts (*P* < 0.05).

Transcriptome mapping results were analyzed using DESeq2 library (Love et al., 2014, R, Bioconductor). Complete analyzed transcripts are available in Supplementary Data Sheet S1 on the journal’s web site and/or upon request from the corresponding author. A maximum false-discovery rate (Benjamini and Hochberg, 1995) of one false-positive per differential transcript set was applied. Principal Component Analysis (PCA) was applied to log-transformed mapping results after sequencing depth normalization (Love et al., 2014), using FactoMineR library (Lê et al., 2008).

For proteomic analysis, differential expression of proteins in the tissue types was analyzed by ANOVA using the Limma R-package^7^ (*P* < 0.05). Only significantly differentially expressed proteins with similar abundance profile in abundance between EMs and NECs of the three investigated genotypes were considered when interpreting results.

## Results

### Morphological and Microscopic Analyses

**FIGURE 2:**
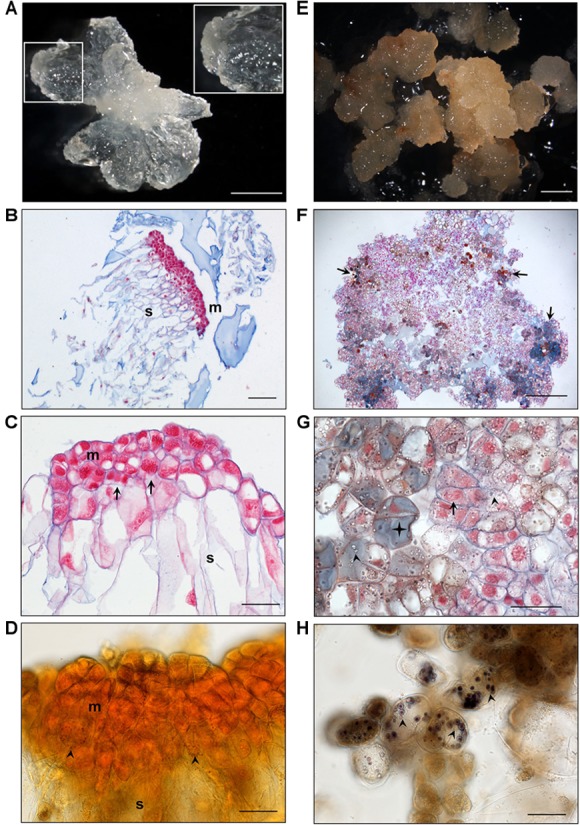
Histological characterization of isogenic embryonal mass (EM, **A–D**) and non-embryogenic callus (NEC, **E–H**) of Douglas-fir. **(A)** Morphology of TD15-1 EM, detail of polyembryogenic center (PC) in the inset; **(B)** Polyembryogenic center of TD15-1 EM, section stained with Nuclear Fast Red/Alcian Blue; **(C)** Small early somatic embryo of SD4-8 EM, note the numerous mitotic figures (metaphase, telophase) in meristematic cells (arrows); **(D)** Rare small starch grains in the lower part of the meristem in a PC of TD15-1 EM (Lugol staining; arrows); **(E)** morphology of TD15-1 NEC; **(F)** arrangement of cells in TD17-1 NEC, note the cells accumulating phenolic compounds (in gray-blue, brownish or amber color, arrows); **(G)** detail of TD17-1 NEC, note dividing cell (arrow) in the vicinity of cells accumulating numerous starch grains (arrowheads) and phenolic compounds (star); **(H)** large starch grains in cells of SD4-8 NEC (arrowheads). Scale bars represent: 2 mm **(A,E,F)**; 200 μm **(B)**; 50 μm **(C,D,G,H)**; m, meristem; s, suspensor.

EMs and NECs cultures derived from all three Douglas-fir genotypes (SD4-8, TD15-1, and TD17-1) fundamentally differed in color, morphology, cell arrangement and levels of secondary metabolites. EMs were usually whitish, yellowish (Figure 2A) or slightly pinkish-white. Cytologically, they consisted of polyembryogenic centers (PCs, Figure 2B) and early SEs (Figure 2C). Cells of both PCs and SEs were organized into bipolar structures with embryonal heads, consisting of meristematic cells, and elongated suspensor cells. Mitotic activity of the meristematic cells was usually very high. A characteristic attribute of EM was scarcity of starch grains. Only small ones were detected in a few cells of the distal parts of the PCs’meristems (Figure 2D). More abundant and larger starch grains were found only in isolated cells from the very distal part of PCs’ suspensors. In some of these cells phenolic compounds also accumulated. Besides these general features of EMs, we found differences among lines (Supplementary Figure S1). Line SD4-8 EM produced smaller PCs and higher amount of small SEs than the other two lines. Both lines TD17-1 EM and SD4-8 EM had more starch grains and phenolic compounds than TD15-1 EM.

Non-embryogenic calli were usually brownish-yellow, light or dark brown, indicating that they had higher levels of phenolic compounds than EMs, as confirmed by histochemical staining (Figure 2E–G). Cells located inside NECs usually formed compact structures with no polar organization, while peripheral cells were arranged more loosely, tended to become detached and accumulated substantial amounts of phenolic compounds in their vacuoles. Some regions of calli were formed by mitotically active cells with no detected phenolic contents (Figure 2G), although dividing cells were quite rare. Almost all NEC cells accumulated starch grains (Figure 2H). Both morphology and secondary metabolite levels differed among the three non-embryogenic lines (Supplementary Figure S1). Line TD15-1 NEC produced the most compact calli and accumulated much fewer starch grains and smaller amounts of phenolic compounds than the other two lines. The highest levels of phenolics and starch were observed in line TD17-1 NEC.

### Biological Comparison of EMs and NECs: Water Content, Embryogenic Potential

**FIGURE 3:**
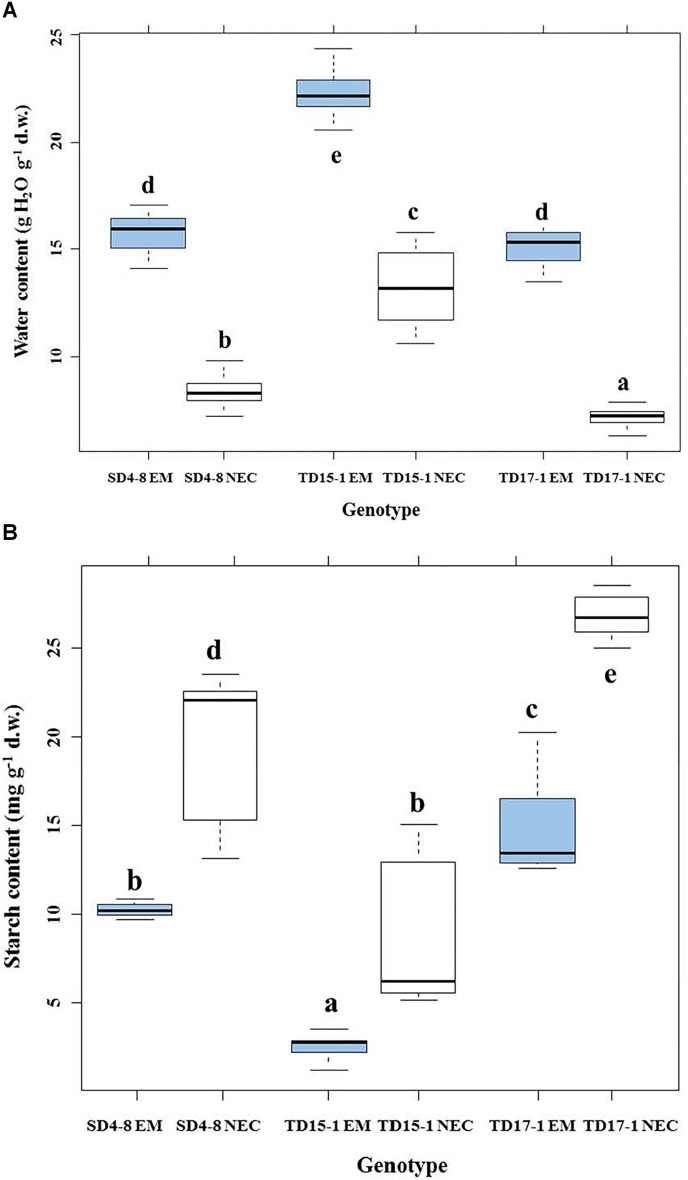
Water content **(A)** and starch content **(B)** in isogenic embryonal mass (EM) and non-embryogenic callus (NEC) of three genotypes (SD4-8, TD15-1, TD17-1) of Douglas-fir during the proliferation phase of somatic embryogenesis. Different letters indicate significant differences according to the multiple comparisons of means (*P* < 0.05, *n* = 20 for water content and *n* = 4 for starch content).

Multifactorial analysis of the water contents of EMs and NECs cultures indicated a significant effect of tissue type (*P* < 2.10^-16^). EMs had significantly higher water content (15.2–22.2 g H_2_O g^-1^ d.w.) than NECs (7.2–13.3 g H_2_O g^-1^ d.w., Figure 3A). TD15-1 EM and NEC had significantly higher water contents than corresponding tissues of lines TD17-1 and SD4-8, while TD17-1 NEC had significantly lower water contents than NEC of the other two lines (Figure 3A).

In maturation experiments, NECs cultures never gave rise to SEs, while all EMs yielded cotyledonary SEs. Mean SE production significantly (*P* < 0.05) varied between the lines: TD17-1, TD15-1, and SD4-8, respectively yielding 268, 815, and 3942 SE g^-1^ f.w.

### Biochemical Comparison of EMs and NECs: Soluble Carbohydrate, Starch and Total Protein Contents

Soluble sugar levels varied between genotypes and tissue types (*P* < 2.10^-16^). Total levels of soluble sugars significantly differed between EMs and NECs (*P* < 5.17 10^-8^) (Table 1). Generally, contents of all sugars were low, and rather variable between genotypes (Supplementary Table S1). No galactose or melibiose were detected in NECs. Maltose, present in the culture medium, was not detected in EMs of SD4-8 and TD17-1 lines. Glucose was the most abundant sugar in both tissue types, and significantly more abundant in the EMs than the NECs, although it was only measured in the TD15-1 and SD4-8 lines. The (Glc + Fruc)/Suc ratio was significantly higher in EMs (*P* < 4.64 10^-4^) than in NECs. The starch concentration was significantly higher in NECs than in EMs tissues (*P* < 8.79 10^-9^; Table 1) of all genotypes (Figure 3B). However, no significant differences in total protein contents were detected between either tissues (*P* < 0.787, Table 1) or genotypes (data not shown).

**Table 1:** Soluble sugar, starch and soluble protein contents of isogenic embryonal mass (EM) and non-embryogenic callus (NEC) of Douglas fir after 2 weeks of multiplication: means obtained from analyses of three genotypes (SD4-8, TD15-1, and TD17-1), *n* = 4 for carbohydrate and starch analyses and 5 for soluble protein determinations (in each case ±SE).

Compounds	EM	NEC
**Carbohydrate content (μg g^-1^ d.w.)**		
Fructose (Fru)	17.14 ± 4.27 ^ab^	10.78 ± 12.80 ^ab^
Galactose	2.68 ± 0.77 ^a^	0.00 ^a^
Glucose (Glc)	208.40 ± 32.74 ^d^	90.79 ± 76.97 ^c^
Sucrose (Suc)	41.23 ± 19.53 ^b^	26.87 ± 12.93 ^ab^
Maltose	6.09 ± 0.76 ^a^	8.95 ± 12.53 ^a^
Melibiose	3.46 ± 0.82 ^a^	0.00 ^a^
Myo-inositol	3.30 ± 0.82 ^a^	4.03 ± 0.66 ^a^
Raffinose	11.58 ± 5.12 ^ab^	5.33 ± 3.92 ^a^
Carbohydrate total	869.47 ± 70.00 ^e^	440.25 ± 43.32 ^f^
[(Glc + Fru)/Suc]	7.50 ± 1.49 ^*^	3.14 ± 0.59 ^**^
Starch (mg g^-1^ d.w.)	9.03 ± 0 0.89 ^α^	19.01 ± 1.37 ^β^
Soluble proteins (mg g^-1^ d.w.)	94.19 ± 7 0.54 ^A^	92.27 ± 27.85 ^A^


*Superscript letters and asterisks indicate significance differences between EM and NEC according to multiple comparison of means (*P* < 0.05).*

### Phytohormone Analysis

Considerable differences were detected in concentrations of measured phytohormones (auxins, ABA and CKs) between EMs and NECs of all three genotypes (SD4-8, TD15-1, and TD17-1).

**FIGURE 4:**
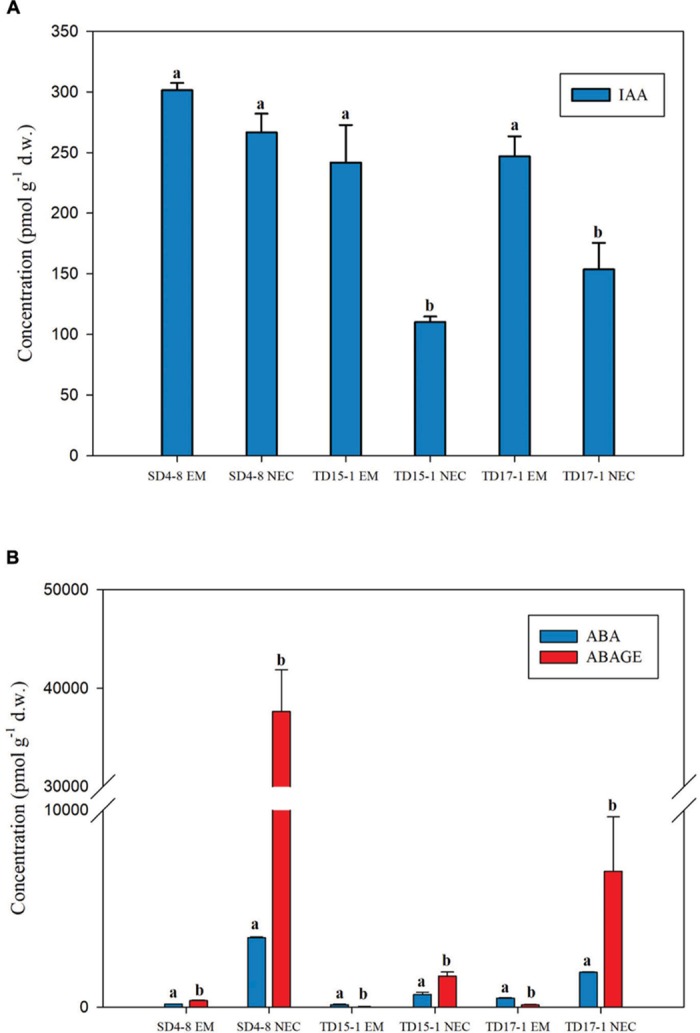
Concentrations of the auxin indole-3-acetic acid (IAA, **A**), abscisic acid (ABA) and ABA-glucose ester (ABA-GE) **(B)** in isogenic embryonal mass (EM) and non-embryogenic callus (NEC) of three genotypes (SD4-8, TD15-1, TD17-1) of Douglas-fir in the proliferation phase of somatic embryogenesis. All data are means ± standard errors for 3–4 replicates. Different letters indicate significant differences according to the multiple comparisons of means (*P* < 0.05).

Free IAA was the only auxin detected, at levels ranging from 241.81 to 301.45 pmol g^-1^ d.w. in EMs (in TD15-1 EM and SD4-8 EM, respectively) and 110.15–266.58 pmol g^-1^ d.w. in NECs (in TD15-1 NEC and SD4-8 NEC, respectively). IAA levels were 1.1- to 2.2-fold higher in EMs than in NECs of all genotypes with statistically significant differences in two out of three lines (TD15-1 and TD17-1) (Figure 4A).

In contrast, contents of the “stress hormone” ABA and its conjugated storage form ABA-GE varied from tens to ten thousands of picomols per gram d.w. and were respectively 3.8- to 22.7-fold and 41.7- to 112.4-fold higher in NECs than EMs. In all NECs, ABA-GE was more abundant than ABA (1.9-, 3.3-, and 9.2-fold in TD15-1 NEC, TD17-1 NEC, and SD4-8 NEC, respectively). ABA-GE levels exceeded ABA levels in the SD4-8 EMs (1.9-fold), but ABA was ca. 4-fold more abundant than ABA-GE in the other two embryogenic lines (Figure 4B).

**FIGURE 5:**
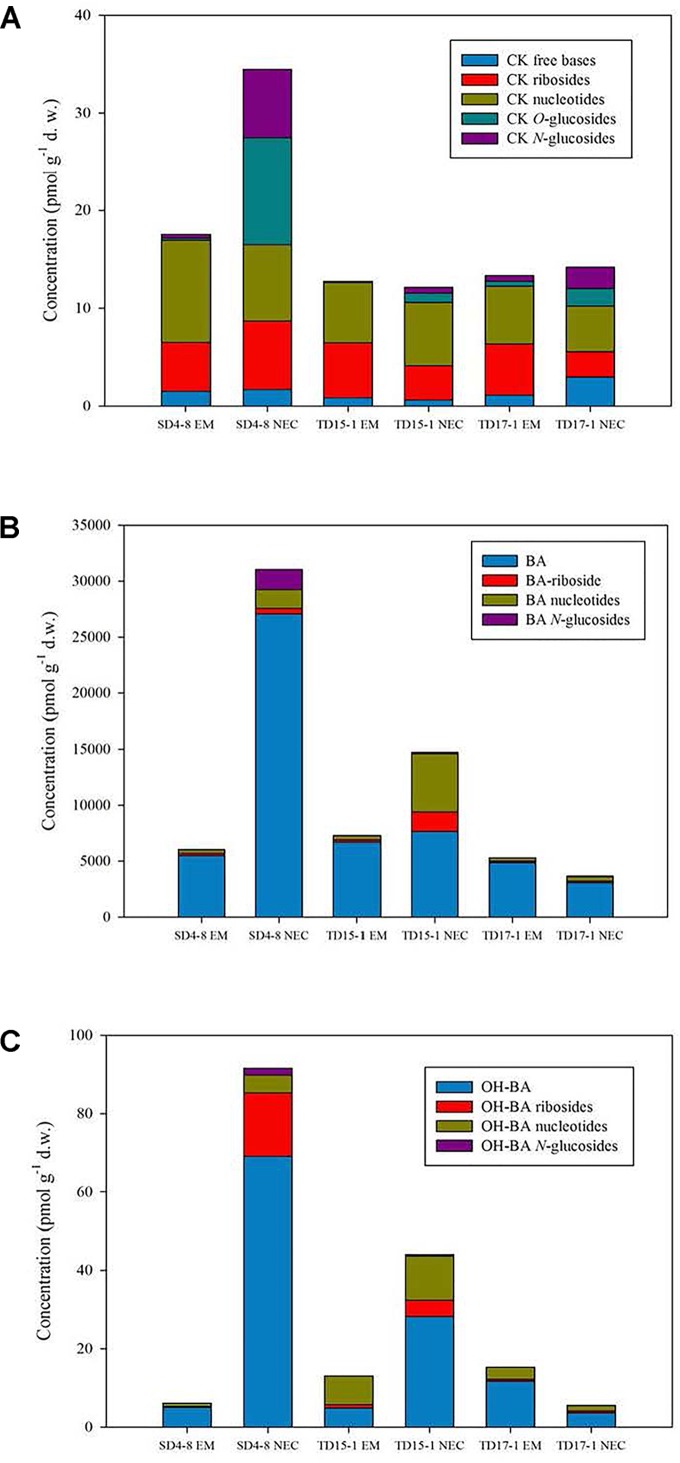
The concentrations of isoprenoid cytokinins (CKs; **A**), aromatic CKs **(B)** and hydroxylated *N*^6^-benzyladenine (BA) derivatives **(C)** in isogenic embryonal mass (EM) and non-embryogenic callus (NEC) for three genotypes (SD4-8, TD15-1, TD17-1) of Douglas-fir at the proliferation phase of somatic embryogenesis. The following isoprenoid and aromatic CK derivatives were detected: CK free bases: *N^6^*-(Δ^2^-isopentenyl)adenine. CK ribosides: *trans*-zeatin 9-riboside, *cis*-zeatin 9-riboside, *N*^6^-(Δ^2^-isopentenyl)adenosine. CK nucleotides: *cis*-zeatin 9-riboside-5’-monophosphate, *N^6^*-(Δ^2^-isopentenyl)adenosine-5^′^-monophosphate, dihydrozeatin 9-riboside-5^′^-monophosphate. CK *O-*glucosides: *trans*-zeatin *O*-glucoside, *cis*-zeatin 9-riboside *O*-glucoside. CK *N-*glucosides: *trans*-zeatin-*N*7-glucoside, *cis*-zeatin-*N*7-glucoside, dihydrozeatin-*N*7-glucoside. BA: *N*^6^-benzyladenine. BA riboside: BA-9-riboside. BA nucleotide: BA-9-riboside-5^′^- monophosphate. BA *N-*glucosides: BA-*N*3-glucoside, BA-*N*7-glucoside, BA-*N*9-glucoside. OH-BA: *para*-hydroxy-BA, *meta*-hydroxy-BA, *ortho*-hydroxy-BA. OH-BA ribosides: *para*-hydroxy-BA-9-riboside, *meta*-hydroxy-BA-9-riboside. OH-BA nucleotides: *para*-hydroxy-BA-9-riboside-5^′^-monophosphate, *meta*-hydroxy-BA-9-riboside-monophosphate, *ortho*-hydroxy-BA-9-riboside- monophosphate. OH-BA N-glucosides: *meta*-hydroxy-BA-*N*9-glucoside, *ortho*-hydroxy-BA-*N*9-glucoside.

Wide spectra of endogenous CKs were detected in both EMs and NECs of all three genotypes, including bioactive (free bases), transport (ribosides) and immediate CK biosynthetic precursor (nucleotides) forms, irreversibly deactivated derivatives (*N*-glucosides) of both isoprenoid and aromatic CKs, as well as storage forms (*O-*glucosides) of some isoprenoid CKs (Figure 5). The total isoprenoid CK concentration varied from 12.14 to 34.48 pmol g^-1^ d.w. (in TD15-1 NEC and SD4-8 NEC, respectively) while aromatic CK levels were substantially (two orders of magnitude) higher, due to presence of *N*^6^-benzyladenine (BA) in the proliferation medium, ranging from 3,663.28 pmol g^-1^ d.w. (in TD17-1 NEC) to 31,034.76 pmol g^-1^ d.w. (in SD4-8 NEC) (Figure 5, Supplementary Figure S2, and Supplementary Table S2).

The most abundant isoprenoid CKs were *cis*Z- and iP-types in all lines, and were maximal in SD4-8 EMs and NECs (16.42 and 28.59 pmol g^-1^ d.w., respectively). In TD15-1 and TD17-1 lines, overall concentrations of *cis*Z- and iP-type CKs ranged from 11.34 to 11.92 pmol g^-1^ d.w. in EMs and 10.54 to 10.75 pmol g^-1^ d.w. in NECs (Supplementary Table S2). Levels of *trans*Z- and DHZ-types were considerably lower than *cis*Z and iP levels in all lines (Supplementary Figure S2 and Supplementary Table S2).

Only two forms of *cis*Z-type CKs (ribosides and nucleotides) were found in EMs of all three genotypes but relatively high levels of other *cis*Z derivatives (*N7*- and *O*-glucosides) were detected in NECs (e.g., 3.48 pmol g^-1^ d.w. of *cis*Z7G and 9.21 pmol g^-1^ d.w. of *cis*ZROG in SD4-8 NEC). The bioactive free base *cis*Z was not found in any examined tissues. The nucleotide iPRMP was the most abundant iP-type CK in both EMs and NECs, followed by iP and its riboside iPR. However, no *N*-glucosides of iP or *N9*-glucosides of any CK were detected in either EMs or NECs (Supplementary Table S2).

Proportions of bioactive and transport CKs (free bases and ribosides) in the total CK pool were lower in NECs than in EMs of all three genotypes (Figure 5A), while their contents of inactive and/or weakly active CK forms (*N7*- and mainly *O*-glucosides) were higher. The difference in relative amounts of active and inactive CKs was apparently driven by differences in CK production and metabolism. The differences were strongest between lines of genotype SD4-8, where they resulted (*inter alia*) in almost two-fold higher levels of total CKs in SD4-8 NEC than in the SD4-8 EM (34.48 and 17.54 pmol g^-1^ d.w., respectively). Interestingly, in addition to the previously mentioned *N7*- and *O*-glucosides of *cis*Z, production of *trans*Z7G and *trans*ZOG in the NECs (but not EMs) was also apparently involved in the metabolic differences. The prevailing isoprenoid CK forms were CK nucleotides, at levels ranging from 4.68 pmol to 7.82 pmol g^-1^ d.w. in NECs (in TD17-1 NEC and SD4-8 NEC, respectively) and 5.9–10.45 pmol g^-1^ d.w. in EMs (in TD17-1 EM and SD4-8 EM, respectively). Analogously to the bioactive and transport CK forms, CK nucleotides were more abundant in EMs than in NECs of two genotypes (SD4-8 and TD17-1), but their levels were comparable in EMs and NECs of TD15-1 (6.19 and 6.43 pmol g^-1^ d.w., respectively, Supplementary Table S2).

Overall contents of aromatic CKs were considerably higher in SD4-8 NEC and TD15-1 NEC than in corresponding EMs, but slightly higher in TD17-1 EM than in TD17-1 NEC (Figure 5B and Supplementary Table S2). In the total pool of aromatic CKs, exceptionally high concentrations of BA (1000 or 10,000 of picomols per gram d.w.) were found in both EMs and NECs of all three genotypes due to presence of BA in the proliferation medium. BA levels were much higher in the SD4-8 NEC (27,048.7 pmol g^-1^ d.w.) than in SD4-8 EM (5,486.1 pmol g^-1^ d.w.), but there were no striking differences in BA concentrations between the other pairs of EMs and NECs. Analogously to isoprenoid CKs, there were lower proportions of free BA and its riboside BAR in the total pool of BA-type aromatic CKs in all three NECs than in corresponding EMs, but higher levels of BA nucleotides and other BA derivatives (*N3*-, *N7*-, and *N9*-glucosides). However, even in the NECs levels of the derivatives were lower than levels of free BA and BAR.

Moderate concentrations (picomols to tens of picomols per gram d.w.) of BA derivatives hydroxylated on the sidechain phenyl ring in *ortho, meta* and *para* positions were also detected in both EMs and NECs of all three genotypes. Analogously to BA and its metabolites, levels of BA hydroxyderivatives were higher in SD4-8 NEC and TD15-1 NEC than in corresponding EMs, but comparable in TD17-1 NEC and TD17-1 EM (Figure 5C and Supplementary Table S2).

To summarize, EMs of all three genotypes were characterized by higher content of auxin IAA (although statistically significant differences only in two out of three lines) whereas the levels of ABA and its conjugated storage form ABA-GE in NECs exceeded those in EMs. Concentrations of bioactive, transport and prevailing biosynthetic precursor forms of CKs were higher in EMs compared to NECs while the inactive and/or weakly active CK forms were detected mainly in NECs.

### Transcriptomic Profiles

#### Global Expression

RNAseq analysis with 4–7 biological repetitions revealed very similar differences between EMs and NECs of all three genotypes. Our Illumina short-reads mapped to 53,185 (97%) of the 54,830 sequences compiled in the Douglas-fir reference transcriptome (Neale et al., 2017). PCA clearly separated transcriptomes of NECs and EMs, principal components 1 and 2 respectively accounting for 75% of the global variance in transcriptomes for all genotypes.

Of the mapped transcripts, 8,955 were differentially expressed in EMs and NECs (4,149 more strongly expressed in EMs than in NECs, and 4,806 more strongly in NECs). Of these, 8,152 (91%) were assigned at least one functional annotation by Blast2GO. Complete GO annotation, in terms of all three ontologies (Molecular Function, MF; Biological Process, BP; and Cellular Component, CC), was obtained for 2,029 transcripts (23%) of the transcripts.

#### Gene Set Enrichment Analysis (GSEA)

Most GSEA results were obtained from Molecular Function (MF) ontology annotations. In some cases, BP ontology provided support at another level of complexity, and in a single case, Cellular Component (CC) ontology supported MF ontology results. Briefly, early EM events seem to include reorganization of sugar metabolism at transcriptome level and deep reprogramming of the ribosomic protein production and post-maturation system. In contrast, key features of NEC formation seem to include upregulation of genes encoding diverse trans-membrane transporters for water, ions, metals, sugars, amino acids and lipids, numerous homeobox leucine zipper and bHLH transcription factors as well as genes involved in phenolic secondary metabolism.

#### Transcripts Up-Regulated in EMs

**FIGURE 6:**
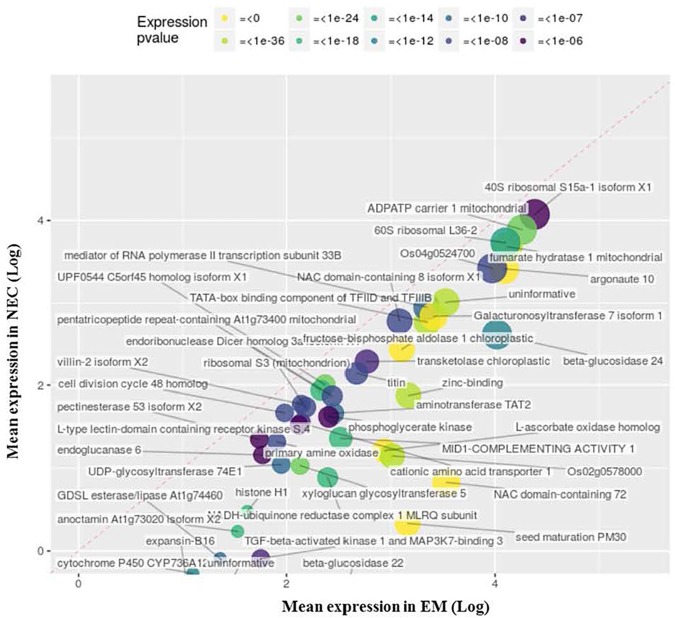
Upregulated transcripts in EM (relative to NEC) annotated to MF GO:0003735, “structural constituent of ribosome.” Expression values are normalized mapping scores from DESeq2 analysis: means for NEC and EM obtained from analyses of 16 and 21 samples, respectively. Transcript labels are from annotations obtained by Blast2GO. The dashed red line indicates non-differential expression between EM and NEC. Differential expression *p*-values are color-coded, and sizes of dots are proportional to mapping scores.

The most strongly upregulated sets of transcripts in EMs (relative to levels in NECs), were 45, 29 and 96 transcripts annoted with MF GO terms GO:0003735 (structural constituent of ribosomes, Figure 6), GO:0016757 (transferase activity, transferring glycosyl groups, Supplementary Figure S3) and GO:0003723 (RNA binding, Supplementary Table S3). *P*-values of their differential expression were < 2 10^-87^, <4 10^-7^ and < 2 10^-86^, respectively.

The most strongly upregulated transcripts of enriched group MF GO:0003735 were PSME_00003749-RA (“seed maturation PM30”; 580-fold differentially expressed, mapping score 1,446 in EMs) (Figure 6), and PSME_00050941-RA encoding a “NAC domain-containing” protein (480-fold differentially expressed, mapping score 3,400). These transcripts exhibited remarkable expression selectivity in EMs. The significance of this transcript set is supported by: BP GO:0006417 (*P* < 1 10^-90^), “regulation of translation”; BP GO:0006412 (*P* < 1 10^-112^), “translation”; and the only supportive CC term, GO:0005840 (*P* < 2 10^-78^), “ribosome.”

The most differentially (65-fold) and highly expressed (mapping score 350) transcript in the MF GO:0016757 group (Supplementary Figure S2) is related to “DNA-directed RNA polymerase IV and V subunit 2” (PSME_00055005-RA), sharing common protein motifs with glycosyltransferases. The second most differentially (35-fold) and highly expressed (mapping score 827) transcript is annotated as a “cyanogenic beta-glucosidase isoform X1” (PSME_00002211-RA) involved in protein *N*-glycosylation.

#### Transcripts Up-Regulated in NECs

**FIGURE 7:**
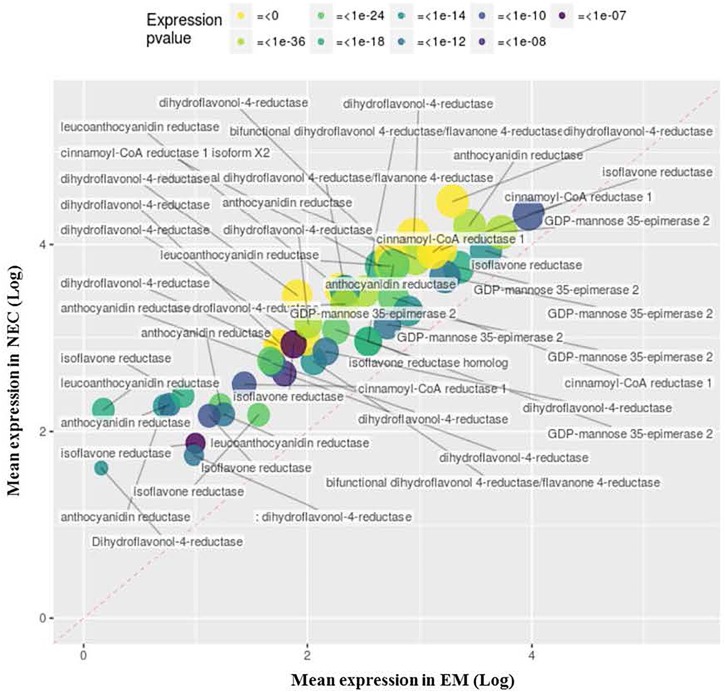
Upregulated transcripts in NEC (relative to EM) annotated to MF GO:0003854, “3-beta-hydroxy-delta5-steroid dehydrogenase activity.” Expression values are normalized mapping scores from DESeq2 analysis: means for NEC and EM obtained from analyses of 16 and 21 samples, respectively. Transcript labels are from annotations obtained by Blast2GO. The dashed red line indicates non-differential expression between EM and NEC. Differential expression p-values are color-coded, and sizes of dots are proportional to mapping scores.

Enrichment of 19 GO terms was detected in NECs (Table 2), involving upregulation (relative to levels in EMs) of 66 transcripts annotated to MF GO:0003854 (3-beta-hydroxy-delta5-steroid dehydrogenase activity) (*P* < 2 10^-22^), 48 of which are associated with flavonoid metabolism (Figure 7). These transcripts were highly differentially expressed (up to 34-fold for PSME_00045373-RA) and expressed (mapping scores up to 29,552, for PSME_00044321-RA). The significance of this transcript set is also supported by BP GO:0006694 (*P* < 3 10^-19^), “steroid biosynthetic process.”

**Table 2:** MF ontology terms enriched in non-embryogenic callus (NEC), numbers of transcripts annotated to the terms detected in NEC and isogenic embryonal mass (EM), GOid and GO code for each term obtained by BLAST2GO and *P*-values obtained by topGO (R package).

GO id	EM	NEC	*P*-value	GO terms
GO:0005506	26	131	3 10^-19^	*Iron ion binding*
GO:0016705	13	117	5 10^-18^	*Oxidoreductase activity, acting on paired donors, with incorporation or reduction of molecular oxygen*
GO:0016887	50	109	4 10^-5^	*ATPase activity*
GO:0043531	16	94	8 10^-32^	*ADP binding*
GO:0050662	22	86	3 10^-12^	*Coenzyme binding*
GO:0016616	24	79	1 10^-9^	*Oxidoreductase activity, acting on the CH-OH group of donors, NAD or NADP as acceptor*
GO:0003854	16	66	2 10^-22^	*3-beta-hydroxy-delta5-steroid dehydrogenase activity*
GO:0046983	19	51	1 10^-3^	*Protein dimerization activity*
GO:0005215	13	49	5 10^-5^	*Transporter activity*
GO:0043565	17	41	9 10^-5^	*Sequence-specific DNA binding*
GO:0016773	19	41	2 10^-53^	*Phosphotransferase activity, alcohol group as acceptor*
GO:0008289	9	33	1 10^-8^	*Lipid binding*
GO:0022857	15	30	3 10^-3^	*Transmembrane transporter activity*
GO:0008236	4	28	9 10^-16^	*Serine-type peptidase activity*
GO:0016829	12	27	2 10^-3^	*Lyase activity*
GO:0019001	3	21	1 10^-5^	*Guanyl nucleotide binding*
GO:0016614	2	18	1 10^-9^	*Oxidoreductase activity, acting on CH-OH group of donors*
GO:0008233	6	17	6 10^-8^	*Peptidase activity*
GO:0016798	2	13	9 10^-6^	*Hydrolase activity, acting on glycosyl bonds*


Four upregulated MF GO terms group transcripts associated with membrane transport activity (GO:0005215, Figure 8) and cellular export to some extent (GO:0022857, Supplementary Figure S4; GO:0019001, Supplementary Figure S5; and GO:0016614, data not shown). Another, GO:0005215 (“transporter activity”), groups 49 transcripts annotated as “NRT1/PTR” (24), “aquaporin” (14), “nucleobase-ascorbate transporter” (2) or “peptide transporter” (2). The most strongly differentially expressed (49-fold) of these transcripts (PSME_00049392-RA) is related to “NRT1/PTR2.13” transport, and the most highly expressed is PSME_00000782-RA (“nucleobase-ascorbate transporter,” mapping score 24,947).

**FIGURE 8:**
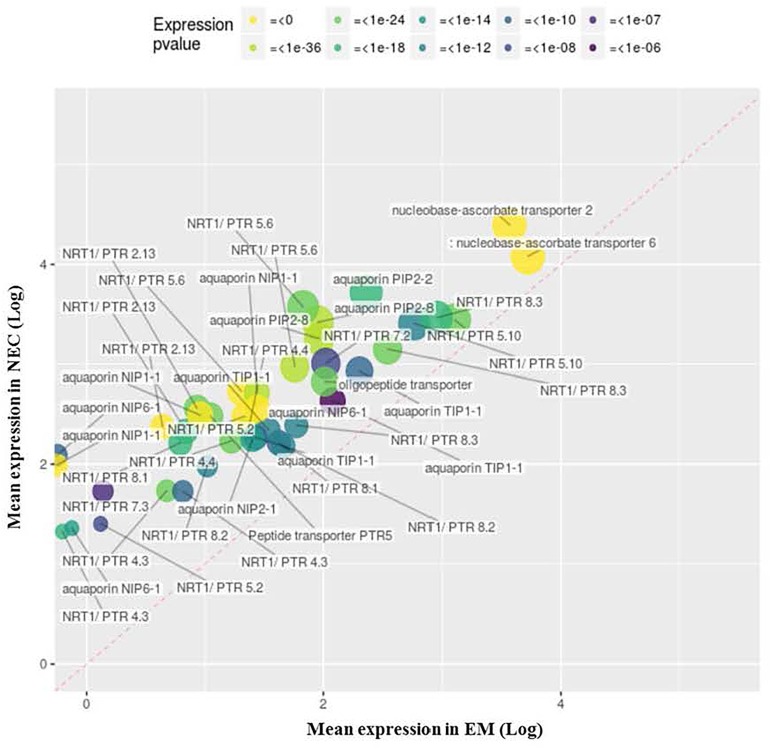
Upregulated transcripts in NEC (relative to EM) annotated to MF GO: 0005215, “transporter activity.” Expression values are normalized mapping scores from DESeq2 analysis: means for NEC and EM obtained from analyses of 16 and 21 samples, respectively. Transcript labels are from annotations obtained by Blast2GO. The dashed red line indicates non-differential expression between EM and NEC. Differential expression *p*-values are color-coded, and sizes of dots are proportional to mapping scores.

Thirty upregulated transcripts are annotated to MF GO:0022857, “transmembrane transporter activity” (Supplementary Figure S4), including 24 encoding sugar transporters with up to 40-fold differences in expression (for PSME_00019229-RA) and mapping scores up to 38,006 (for PSME_00049464-RA). In addition, MF GO:0019001 (“guanyl nucleotide binding,” Supplementary Figure S5) is strikingly enriched by 17 transcripts related to “ABC transporter” or “multidrug resistance” proteins, with up to 148-fold differences in expression (for PSME_00004142-RA) and up to 14,475 mapping scores (for PSME_00039058-RA). Another set of 18 transcripts enriches MF GO:0016614 (“oxidoreductase activity, acting on CH-OH group of donor,” data not shown), dominated by four and 12 transcripts annotated as FAO1/FAO4A and HOTHEAD, respectively.

Upregulated GO terms in NECs included two classes of transcription factors: MF GO:0008289 and MF GO:0046963 (Supplementary Figures S6, S7). Twenty-one out of 33 genes assigned to GO:0008289 (“homeobox-leucine zipper”) in Douglas-fir were upregulated; the most differentially expressed (34-fold) was PSME_00034712-RA while the most highly expressed (mapping score 28,232) was PSME_00017677-RA. Thirty-nine out of 51 genes assigned to GO:0046963, all with bHLH-related annotations, were upregulated; the most differentially expressed (82-fold), was PSME_00010577-RA, and the most highly expressed was PSME_00039747-RA (mapping score 17,892).

### Proteomic Analysis

**FIGURE 9:**
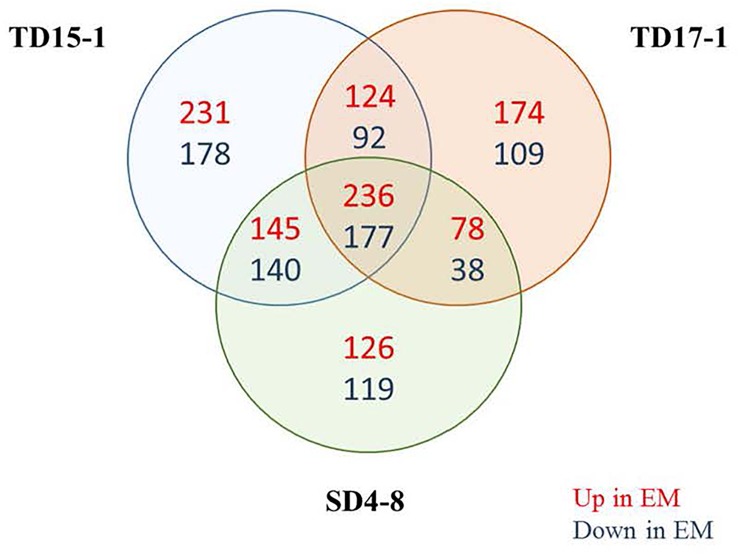
Venn diagram showing overlaps of the identified proteins (3028) in isogenic embryonal mass (EM) and non-embryogenic callus (NEC) of the three genotypes SD4-8, TD15-1 and TD17-1 of Douglas-fir. In total 413 proteins were significantly differentially expressed between EMs and NECs of all three genotypes: 236 more strongly expressed in EMs than in NECs and 177 more strongly expressed in NECs. Interpretation of results of the proteomic study was based on expression patterns of these 413 proteins.

Further differences between EMs and NECs of the three genotypes were explored by nLC-MS/MS-based quantitative proteome characterization. A global profiling of quantitative proteome was obtained for these tissues. In total, 3,028 proteins were identified (Supplementary Data Sheet S2). Among the 2619 differentially expressed proteins (ANOVA, *p* < 0.05), only 413 (15.8% of the significant proteins) significant for the three genotypes and with the same tissue abundance profile were considered (Supplementary Table S4). Of the 413 proteins, 236 and 177 were more strongly expressed in EMs and NECs, respectively (Figure 9). Then the abundance of about 14% of identified proteins changed between EMs and NECs. PCA clearly separated proteomes of the samples (Supplementary Figure S8). Axis 1 and 2 explained 71 and 7% of the total variance in expression levels of the 413 proteins, and mainly separated samples according to tissue type and genotype, respectively. Functional GO-based classifications of the 413 significantly differentially expressed proteins (Supplementary Table S5) indicate that large proportions of proteins upregulated in EMs, relative to NECs, are associated with metabolic processes and cellular differentiation or division (30.6 and 24.0%, respectively). Moreover, 36.4 and 16.7% of proteins downregulated in EMs are respectively associated with these processes. In addition, 9.4% of upregulated proteins in EMs are associated with localization and 4.5% of downregulated ones with responses to stimuli.

#### Sub-network Enrichment Analysis

**FIGURE 10:**
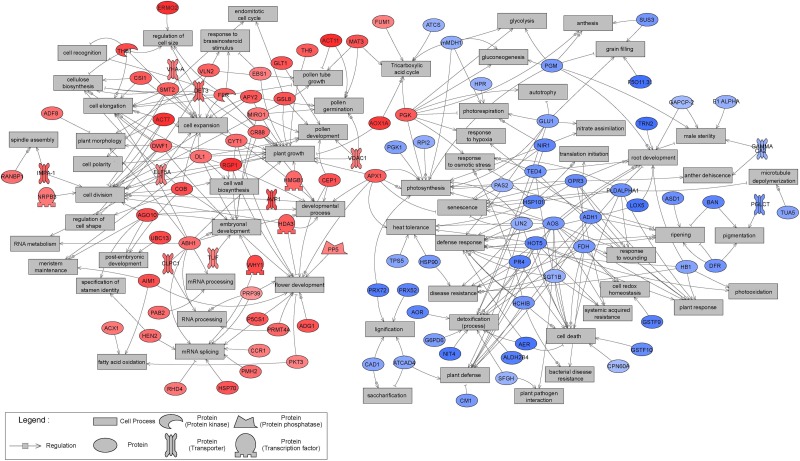
Results of Sub-Network Enrichment Analysis (SNEA) connecting significantly differentially expressed (*P* < 0.05) proteins and regulators or targets in isogenic embryonal mass (EM) and non-embryogenic callus (NEC) of three genotypes (SD4-8, TD15-1, TD17-1) of Douglas-fir in the proliferation phase of somatic embryogenesis detected in proteomic studies. The proteins are named according to homologs in *Arabidopsi*s. The correspondences between Douglas-fir and *Arabidopsis* protein names are given in Supplementary Table S4. Red, blue and gray indicate enrichment in EM and NEC or absence, respectively, and the colors’ intensities indicate relative expression levels.

Sub-network enrichment analysis of the significant proteins, based on the *Arabidopsis thaliana* bibliographic database, and hence network representation with names of orthologous *Arabidopsis* proteins, provided further indications of their functions, interactions and putative targets, as well as regulators involved in metabolic pathways that may be affected by tissue type. Non-isolated protein networks with a minimum of two protein connections were selected for further analysis. However, not all significantly differentially expressed proteins were represented, and only those with known orthologs in *Arabidopsis thaliana* were considered. Overall, 57 and 50 proteins significantly up- and down-regulated in EMs, relative to NECs, respectively, were integrated in the constructed networks (Figure 10). The distribution of significant proteins in different BPs has an important partition according to the tissue. The proteins overexpressed in EMs are linked with processes such as “cell division,” “cell expansion,” “cell elongation,” “developmental process” and “embryonal development,” while those more strongly expressed in NECs are involved in processes such as “cell death,” “defense response,” “detoxification (process)” or “plant defense” (Table 3).

**Table 3:** Significantly differentially expressed proteins related to the biological processes identified in the SNEA (see Figure 10).

Biological process	Overlap	% overlap	Overlapping Entities	*p*-value	Exp.
Plant growth	18	1	AGO10; CR88; DWF1; ABH1; EBS1; ELF5A-1; AOX1A; DET3; APX1; TH9; DL1; CYT1; APY2; RGP1; GSL8; HMGB1; FER; VDAC1	2.7 10^-02^	EM
Cell death	13	1	LIN2; mMDH1; AOS; HCHIB; SGT1B; HOT5; AER; GSTF10; FDH; TED4; PR4; PLDALPHA1; CPN60A	6.6 10^-03^	NEC
Cell division	13	1	AGO10; MIRO1; SMT2; DWF1; ELF5A-1; ACT7; IMPA-1; COB; APY2; AVP1; RGP1; HDA3; NRPB3	5.8 10^-03^	EM
Defense response	13	1	LIN2; GLU1; TPS5; AOS; HCHIB; SGT1B; LOX5; HOT5; AER; FDH; HB1; PR4; OPR3	1.3 10^-03^	NEC
Flower development	13	1	P5CS1; PRMT4A; AIM1; DWF1; APX1; PP5.2; CYT1; RGP1; ADG1; PRP39; HDA3; PKT3; CEP1	4.9 10^-02^	EM
Photosynthesis	13	1	PGM; PGK1; LIN2; mMDH1; GLU1; AOS; APX1; PGK; PAS2; PRX72; NIR1; RPI2; OPR3	1.3 10^-03^	NEC
Cell expansion	12	3	CSI1; SMT2; THE1; ELF5A-1; DET3; DL1; ACT7; COB; CYT1; VHA-A; APY2; FER	5.1 10^-05^	EM
Detoxification (process)	12	4	GLU1; AOS; G6PD6; HOT5; SFGH; AER; NIR1; FDH; ALDH2B4; NIT4; ADH1; AOR	2.0 10^-07^	NEC
Developmental process	9	1	DL1; CYT1; AGO10; AVP1; ABH1; HDA3; APX1; FER; CEP1	1.6 10^-02^	EM
Plant defense	9	1	HOT5; LIN2; ATCAD4; CM1; AOS; HCHIB; PR4; ADH1; SGT1B	2.2 10^-02^	NEC
Root development	9	1	HOT5; PGM; GAPCP-2; TRN2; AOS; HB1; TED4; SGT1B; LOX5	3.1 10^-02^	NEC
Embryonal development	9	2	DL1; COB; CYT1; AGO10; CR88; MIRO1; TUF; WHY1; CLPC1	6.8 10^-03^	EM
mRNA splicing	9	4	P5CS1; PMH2; PRP39; WHY1; ABH1; HEN2; RHD4; CCR1; HSP70	4.6 10^-05^	EM
Cell elongation	8	2	ACT7; DWF1; CSI1; SMT2; THE1; ABH1; FER; DET3	1.1 10^-02^	EM
Disease resistance	7	1	HOT5; LIN2; FDH; HSP90.1; HB1; PR4; SGT1B	2.2 10^-02^	NEC
Plant response	7	1	HOT5; AOS; HB1; ADH1; PLDALPHA1; TED4; OPR3	2.9 10^-02^	NEC
Ripening	7	1	HOT5; DFR; FDH; ASD1; ADH1; BAN; PAS2	9.7 10^-03^	NEC
Senescence	7	1	LIN2; NIR1; AOS; TED4; PLDALPHA1; OPR3; APX1	4.5 10^-02^	NEC
Pollen development	7	2	DL1; MIRO1; RGP1; GSL8; GLT1; CEP1; VDAC1	4.7 10^-03^	EM
Cellulose biosynthesis	7	6	DL1; COB; SMT2; CSI1; THE1; DET3; FER	3.9 10^-05^	EM
Heat tolerance	6	2	HOT5; HSP90.1; TPS5; AOS; APX1; HSP101	3.1 10^-03^	NEC
Plant morphology	5	2	ACT7; VLN2; DWF1; ABH1; ADF8	1.8 10^-02^	EM
Pollen germination	5	2	MIRO1; MAT3; ACT11; APY2; VDAC1	1.6 10^-02^	EM
Pollen tube growth	5	2	VLN2; MAT3; MIRO1; ACT11; FER	4.2 10^-02^	EM
Response to osmotic stress	5	3	HCHIB; PLDALPHA1; TED4; PGK; HSP101	2.0 10^-03^	NEC
Male sterility	5	4	E1 ALPHA; PGM; GAPCP-2; GAMMA CA2; OPR3	1.2 10^-03^	NEC
Photorespiration	5	6	HPR; mMDH1; NIR1; GLU1; PGK	1.7 10^-04^	NEC
Lignification	4	2	PRX72; ATCAD4; PRX52; CAD1	2.6 10^-02^	NEC
Regulation of cell size	4	2	CYT1; ERMO2; THE1; FER	3.7 10^-02^	EM
Systemic acquired resistance	4	2	HOT5; AOS; OPR3; SGT1B	3.9 10^-02^	NEC
Response to wounding	4	3	FDH; AOS; PLDALPHA1; OPR3	6.7 10^-03^	NEC
Grain filling	4	4	PGM; GLU1; SUS3; F5O11.31	3,0 10^-03^	NEC
Tricarboxylic acid cycle	4	4	MAT3; AOX1A; FUM1; PGK	9.2 10^-03^	EM
RNA processing	4	7	PRMT4A; PRP39; ABH1; CLPC1	1.0 10^-03^	EM
Anthesis	3	2	PGM; SUS3; PGK	3.4 10^-02^	NEC
Cell redox homeostasis	3	2	FDH; AOS; GSTF9	3.0 10^-02^	NEC
Glycolysis	3	2	PGM; mMDH1; PGK	3.4 10^-02^	NEC
Pigmentation	3	2	DFR; PGLCT; BAN	4.0 10^-02^	NEC
Fatty acid oxidation	3	3	AIM1; ACX1; PKT3	3.9 10^-02^	EM
Meristem maintenance	3	3	DL1; AGO10; AIM1	4.2 10^-02^	EM
Tricarboxylic acid cycle	3	3	mMDH1; ATCS; PGK	2.5 10^-02^	NEC
Cell wall biosynthesis	3	4	DL1; COB; RGP1	1.7 10^-02^	EM
Regulation of cell shape	3	4	ACT7; COB; DWF1	1.7 10^-02^	EM
Gluconeogenesis	3	5	PGM; mMDH1; PGK	6.4 10^-03^	NEC
Response to brassinosteroid stimulus	3	5	DWF1; EBS1; FER	1.3 10^-02^	EM
Post-embryonic development	3	6	COB; UBC13; AGO10	6.7 10^-03^	EM
mRNA processing	3	12	PAB2; ECT2; ABH1	1.1 10^-03^	EM
Anther dehiscence	2	3	GAMMA CA2; OPR3	4.8 10^-02^	NEC
Autotrophy	2	3	GLU1; PGK	5.0 10^-02^	NEC
Nitrate assimilation	2	4	NIR1; GLU1	2.8 10^-02^	NEC
Photooxidation	2	4	HOT5; HB1	3.5 10^-02^	NEC
Plant pathogen interaction	2	4	SFGH; AOS	4.1 10^-02^	NEC
Translation initiation	2	4	FDH; HSP101	3.6 10^-02^	NEC
Cell polarity	2	5	DL1; SMT2	4.2 10^-02^	EM
Saccharification	2	5	ATCAD4; CAD1	2.2 10^-02^	NEC
Spindle assembly	2	5	IMPA-1; RANBP1	3.6 10^-02^	EM
Microtubule depolymerization	2	6	TUA5; PLDALPHA1	1.7 10^-02^	NEC
RNA metabolism	2	6	ABH1; ELF5A-1	3.1 10^-02^	EM
Response to hypoxia	2	8	ADH1; PGK	1.1 10^-02^	NEC
Specification of stamen identity	2	11	AGO10; HEN2	8.1 10^-02^	EM
Bacterial disease resistance	2	20	FDH; HCHIB	1.5 10^-03^	NEC
Endomitotic cell cycle	2	22	SMT2; GSL8	2.0 10^-03^	EM
Cell recognition	2	25	THE1; FER	1.5 10^-03^	EM


*Biological process: name of the group of proteins regulating the biological process. Overlap: number of proteins in this group and present in the tested list (all up- or down-regulated proteins). % overlap: number of proteins in this group as a percentage of total number of proteins in the reference group of the database. Overlapping entities: names of proteins in this group. *p*-value: *p-*value obtained from Fisher’s Exact test of enrichment of groups in the lists of up- and down-regulated proteins). Exp., tissue (embryonal mass or non-embryogenic callus; EM and NEC, respectively) in which group of proteins was upregulated.*

**FIGURE 11:**
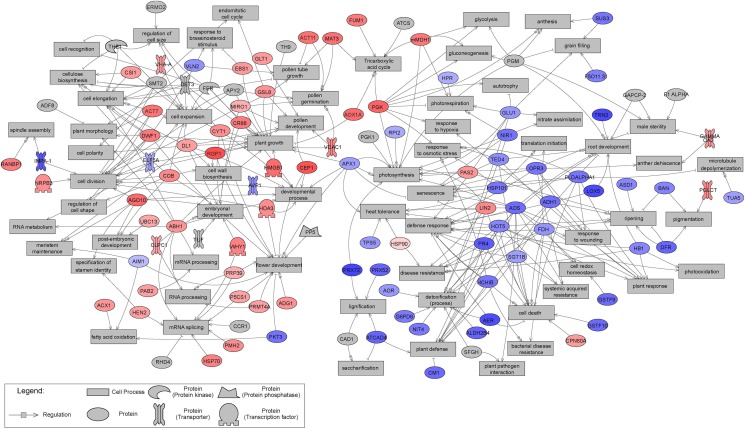
Mapping of significantly differentially expressed (*P* < 0.05) transcripts to sub-network generated from proteomics studies and presented in Figure 10. This transcript Sub-Network Enrichment Analysis (SNEA) map connects transcripts and regulators or targets in isogenic embryonal mass (EM) and non-embryogenic callus (NEC) of three genotypes (SD4-8, TD15-1, TD17-1) of Douglas-fir in the proliferation phase of somatic embryogenesis. The transcripts are named according to homologues in *Arabidopsis*. The correspondences between Douglas-fir and *Arabidopsis* gene names are given in Supplementary Table S4. Red and blue indicate enrichment in EM and NEC, respectively, and the colors’ intensities indicate relative transcript expression levels.

In order to compare the proteomic and transcriptomic results, mapping of significantly differentially expressed transcripts (*P* < 0.05) to sub-network generated from proteomics studies were established (Figure 11). A large concordance of the results were obtained: while 107 significant proteins constitute this sub-network, 88 were confirmed as transcript. The expression pattern was also very similar between transcripts and proteins, with only fourteen transcripts showing an opposite accumulation profile compared to proteins.

## Discussion

### Biological and Cytological Analyses

Maturation yields of embryogenic lines of the three unrelated genotypes (SD4-8, TD15-1, TD17-1) varied from just 268 SEs g^-1^ f.w. (TD17-1) to 3942 SEs g^-1^ f.w. (SD4-8). Similar “genotype” effects are often observed in conifers (Klimaszewska et al., 2016) such as Douglas-fir (Lelu-Walter et al., 2018) and may result from interactions between genotypes and culture conditions. They also reflect differences in EMs structure between lines that we have recently described in detail (Gautier et al., 2018). In contrast, NECs never developed SEs, confirming cytological identification of their non-embryogenic status (Figure 2). NECs, which were brown due to the presence of phenolic compounds, had higher starch contents than EMs (Figure 2), as confirmed by carbohydrate analysis (Table 1).

### Sugar Metabolism

The (Glc + Fru)/Suc ratio (Table 1), which indicates sucrose hydrolysis activity and tissues’ energy status, was higher in EMs than in NECs. This pattern, previously observed in conifers (Lipavská and Konrádová, 2004; Navarro et al., 2017), confirms a need for an increase in levels of reducing sugars during embryonic cell multiplication, to support the high frequencies of cell divisions (as illustrated in Figure 2). In contrast, cells in NECs were oriented toward energy storage in starch (Figure 2 and Table 1). Similar differences in sugar partitioning between embryogenic and NEC have been previously described in alfalfa (Martin et al., 2000). However, there are considerable variations in other taxa. For example, higher concentrations of Glc, Fru, Suc and starch have been observed in EMs than in NECs of sugarcane (Mahmud et al., 2015), higher levels of mannose have been observed in EMs than in NECs of mangosteen (Maadon et al., 2016), and sucrose was the only metabolite detected by Businge et al. (2012) in EMs of *Picea abies*, but isomaltose accumulated in a line with blocked embryonic development. Therefore, there is no need to analyze further the sugar composition distribution in relation to the tissue type because the composition seems species dependent.

### Phytohormones

Phytohormones of auxin, ABA and CK classes were analyzed in EMs and NECs of the three Douglas-fir genotypes. Auxins and CKs were examined because they regulate cellular multiplication and differentiation, and changes in their levels correlate with multiplication phases of EMs and NECs. ABA is particularly involved in embryos’ differentiation and maturation (Fehér, 2015), but it also apparently participates in SE initiation, probably by modulating local auxin biosynthesis and polar transport, which is important for establishment of auxin response patterns in *Arabidopsis* (Su et al., 2013).

#### Auxin

Indole-3-acetic acid concentrations were higher in the EMs than in NECs although statistically significant differences were found only in two out of three lines. It suggests an important role of auxins in somatic embryogenesis in Douglas-fir. Auxins’ participation in maintenance of multiplying cells has been demonstrated in somatic embryogenesis of rubber tree (*Hevea brasiliensis*) and hybrid larch where significant differences in IAA levels between EMs and NECs have also been reported (Etienne et al., 1993; Jourdain et al., 1997). However, conflicting indications of auxins’ functions in proliferation (and induction) of various conifer embryogenic cultures have been published, including observations of both inhibitory and stimulatory effects (Nørgaard and Krogstrup, 1991; Liao et al., 2008; Vondráková et al., 2011, 2016).

Upregulation of 10 transcription factors of the *Wuschel-related* *homeobox* family (*WOX11* and *WOX9*) in EMs, including 4420-fold differential expression and a 6928 ± 327 mapping score (*n* = 16) for PSME_00019221-RA recorded in this study supports a role for auxin in somatic embryogenesis of Douglas-fir. Moreover, an auxin receptor TIR1 (Transport Inhibitor Response 1 (FC = 1.5), known to function in root gravitropism was also upregulated in EMs. The WOX transcription factors presumably participate in early determination of embryo development, analogously to their participation in zygotic embryogenesis (Laux et al., 1996; Mayer et al., 1998; Brand et al., 2002; Haecker et al., 2004). These factors also, as has been demonstrated recently, participate in somatic embryogenesis of conifers (Palovaara et al., 2010; Vestman et al., 2011; Trontin et al., 2016b). Identification of differentially expressed genes encoding auxin efflux carriers from the *PIN-like* family involved in polarization of SEs (Gälweiler et al., 1998; Steinmann et al., 1999; Friml et al., 2003) corroborates a substantial role of auxins in somatic embryogenesis.

#### Cytokinins

CKs play key roles in the initiation and further development of embryogenic cultures, as demonstrated by the requirement for aromatic derivatives in media during the induction of conifer somatic embryogenesis and subsequent proliferation (Nørgaard and Krogstrup, 1991; Klimaszewska et al., 2016). Both EMs and NECs of the three tested genotypes had substantially higher (ca. 260- to 1220-fold) concentrations of aromatic CKs than isoprenoid CKs, due to presence of BA in the culture medium. In addition, total contents of aromatic CKs were higher in NECs than in corresponding EMs (except in the TD17-1 genotype), in accordance with findings in hybrid larch (Jourdain et al., 1997) and indicative of either a higher intake of BA from the culture medium or its lower utilization by NECs of Douglas-fir. Analogously, levels of BA metabolites hydroxylated on the sidechain phenyl ring in SD4-8 NEC and TD15-1 NEC exceeded those in the EMs, but in all three genotypes they were 2–3 orders of magnitude lower than those of non-hydroxylated derivatives. This provides the first evidence for the involvement of endogenous hydroxy derivatives of BA in somatic embryogenesis.

Detected concentrations of isoprenoid CKs (largely *cis*Z- and iP-types) were relatively low, similar to those of BA hydroxylated derivatives. *cis*Z-type CKs also predominate throughout Norway spruce somatic embryogenesis according to Vondráková et al. (2018). Distinct changes in levels of three isoprenoid CK forms—iP, its riboside and *cis/trans*Z—have been observed during growth of EMs in hybrid larch (Jourdain et al., 1997). We detected ca. two-fold higher levels of total isoprenoid CKs in NECs than in EMs of the SD4-8 genotype, but no striking differences in this respect could be observed between NECs and EMs of the other two genotypes.

Among the differentially expressed genes, we identified an adenosine kinase (AK) upregulated in EMs, represented by a single upregulated (2-fold) transcript, PSME_00004950-RA, with a mapping score of 36556 ± 1772 (*n* = 16), responsible for phosphorylation of CK ribosides to CK nucleotides. Upregulation of AK may explain enhanced levels of isoprenoid CK nucleotides in SD4-8 EMs. Although CK ribosides and nucleotides were the major isoprenoid CK forms in EMs, their proportions in the total CK pool were smaller in all three NECs, which had considerably higher contents of inactive and/or weakly active CK forms, either irreversibly or reversibly glucosylated (*N7-* and *O*-glucosides, respectively). In addition, *N7*-glucosides of *cis*Z and *trans*Z were only detected in NECs, while CK *N9*-glucosides, which are relatively common in the plant kingdom (e.g., Vaňková, 1999), have not been detected in Douglas-fir EMs and NECs at all. These findings, together with a total absence of *N7*- and *N9*-glucosides in SEs of Norway spruce (Vondráková et al., 2018), indicate that the CK-*N*-glucosyltransferase pathway is inactive or insignificant in conifer somatic embryogenesis. Similarly, bioactive CKs have been shown to promote zygotic embryogenesis in *Pisum sativum* (Quesnelle and Emery, 2007), suggesting that inactive CK forms are probably not involved in somatic embryogenesis. We identified several differentially expressed genes encoding enzymes involved in metabolic pathways of isoprenoid CKs. Two, zeatin *O*-glucosyltransferase responsible for *O*-glucosylation of *trans*Z and CK dehydrogenase catalyzing degradation of isoprenoid CK free bases and ribosides, were upregulated in NECs (fold-differences, 9.0 and 8.7, respectively). Upregulation of expression and activity of these enzymes in NECs of Douglas-fir may also contribute to the higher proportions of bioactive and transport CK forms (free bases and ribosides) in the total CK pool in EMs compared to NECs.

To summarize, using advanced HPLC-MS methodology we analyzed differences in patterns and levels of about 20 CKs and CK derivatives in EMs and NECs of three Douglas-fir genotypes. To our knowledge, this provides the most comprehensive overview to date of profiles of endogenous CKs, including aromatic CKs, during proliferation of conifer SEs.

#### Abscisic Acid

In addition to phytohormones involved in cell cycle regulation, levels of ABA and its (reversible) storage form ABA-GE were examined in EMs and NECs of the three tested genotypes. Levels of ABA and its derivatives are typically low in initial phases of embryonic development in conifers (Stasolla and Yeung, 2003). Accordingly, ABA and ABA-GE levels were rather low in EMs and at most 1–8% of levels in corresponding NECs. These findings are consistent with patterns observed in rubber trees (Etienne et al., 1993), but no differences in levels have been found between EMs and NECs of hybrid larch (Jourdain et al., 1997). Contrary to the patterns observed in larch, high levels of ABA-GE were detected in NECs of all three Douglas-fir genotypes.

Transcripts upregulated in NECs included six (one, PSME_00028832-RA, with a 6-fold difference in expression and 9544 ± 2560 mapping score, *n* = 21) encoding zeaxanthin epoxidase (for which there was a 31.14-fold difference in protein-level expression). Expression of these ABA biosynthetic enzymes is known to be increased, while induction of ABA 8’-hydroxylase (catalyzing oxidative degradation of ABA) is blocked, by water stress (Cutler and Krochko, 1999). Taken together, observed changes in ABA contents and expression of genes involved in ABA metabolism suggest that NECs of Douglas-fir may be under stress.

### Transcriptomic Profiles

#### EM-Favored Expression

##### Ribosome functions and protein maturation are re-organized

A set of 45 transcripts upregulated in EMs annotated to “structural constituent of ribosome” (MF GO:0003735, Figure 6), and a 96-transcript set annotated to MF GO:0003723 (RNA binding, data not shown), suggest a reorientation in EMs toward production of proteins involved in embryogenic cell differentiation. In addition, a 45-transcript set including PSME_00003749-RA (annotation, “seed maturation PM30”) has sequence similarity to *Glycine max* AAD30864.1, encoding a late embryogenic abundant protein involved in acquisition of desiccation tolerance in zygotic embryos (Schmutz et al., 2010; Shih et al., 2012). Detection of this transcript in EMs in early stages suggests that it is expressed well before it is required during SE formation in Douglas-fir (or has unsuspected roles in early stages). Another member of the 45-transcript set is PSME_00050941-RA, linked to a transcription factor family (“NAC domain-containing 72”), widely involved in cell development control through ribosome biogenesis (Ohbayashi and Sugiyama, 2018). For PSME_00050941-RA, homolog of AtRD26 (a.k.a. ANAC72, accession AT4G27410.3), we found a proteic percent identity at 62%, 50% and 46% with *Pinus pinaster* PpNAC15, PpNAC28 and PpNAC17 respectively, (Clustal 2.1 by MUSCLE v3.8.31, Edgar, 2004), differing from PpNAC2 and PpNAC3 (39 and 38% percent identity) reported as stress-response related NAC in *Pinus pinaster* (Pascual et al., 2015). PSME_00050941-RA homolog AtRD26 is reported as a transcription factor induced by dessication and a transcriptional activator in ABA-mediated dehydration response (Fujita et al., 2004) in line with somatic embryo differentiation expected in EMs. These findings, along with other members of this gene set and differentially expressed proteins we detected by nLC-MS/MS (Supplementary Table S5) showing that enzymes involved in protein synthesis and processing were upregulated in EMs, suggest an important reconfiguration of protein production in EMs during early cell differentiation stages.

Another set of 29 transcripts upregulated in EMs, annotated to GO:0016757, encodes CAZymes (Lombard et al., 2014) (Supplementary Figure S3). These include diverse alpha-glycosidases, beta-glycosidases and transglycosylases that cleave glycosidic bonds and transfer glycosyl groups between various compounds, notably amino acids in numerous metabolic pathways (Withers, 2001). Upregulation of this set suggests possible reconfiguration of amino-acid metabolism and protein maturation pathways in EMs’ cells (Supplementary Table S5).

#### NEC-Favored Expression

##### Enhancement of antioxidation capacity through increase in phenolic compounds

Transcripts upregulated in NECs included 48 involved in production of flavonoids (Figure 8), which are steroid-like secondary metabolites (Rosenberg-Zand et al., 2000). Such increases in flavonoid production occur in many plant stress responses (Treutter, 2006) and are consistent with the tissue browning observed during cultivation of our NEC lines (Figure 2E–G).

##### Membrane transport in and out of cell

Putative functions of transcripts associated with another GO term enriched in NECs, GO:0005215 (Figure 6) are associated with transport across cellular membranes of water (Willigen et al., 2004) and diverse substrates: nitrate, peptides, amino acids, dicarboxylates, glucosinolates, IAA, ABA (Léran et al., 2014); and purine (Gournas et al., 2008). IAA export from NECs could explain the low IAA concentrations detected in all three NECs (Figure 4). Similarly, water transport from NECs (Figure 3A) could be related to the higher expression of transcripts involved in production of trans-membrane aquaporins (both plasma membrane intrinsic proteins and nodulin-26 like intrinsic proteins) in NECs, although this would imply additional changes in molecular mechanisms to orient such transport toward export, such as transcripts enriched in the MF GO:0019001 set.

Nearly all of 24 transcripts involved in production of monosaccharide sugar transporters annotated to the GO term MF GO:0022857 were significantly upregulated in NECs (Supplementary Figure S4). These transporters are strongly involved in glucose import from media in many *in vitro* culture systems (Klip et al., 1994) and seed development in plants (Weber et al., 1997). Our results suggest that sugar-sensing in media and monosaccharide import through sugar carriers (Williams et al., 2000) could play major roles in initiation of NECs, and their high subsequent levels of starch storage (Figure 2 and Table 1).

A set of upregulated ABC transporter transcripts associated with MF GO:0019001 (Supplementary Figure S5) are involved in export of diverse cytotoxic molecules (Dawson and Locher, 2006) and have pleiotropic effects in plants (Crouzet et al., 2006). Their upregulation suggests reorganization of non-embryogenic cells toward trans-membrane export of potential toxins (Martinoia et al., 2002), lipids (Pighin et al., 2004), and/or water (Klein et al., 2003). Transcripts associated with MF GO: 0016614 (data not shown) and HOTHEAD or FAO1/FAO4A functions, both of which are involved in biosynthesis of long-chain fatty acids used in the formation of extracellular matrix (Kurdyukov et al., 2006) as lipid barriers (Pollard et al., 2008) were also upregulated. Collectively, these results suggest enhancement of functions oriented toward production of long-chain lipids.

##### Transcription factors involved in cell proliferation

Enrichment of MF GO:0008289 (Supplementary Figure S6) suggests that homeobox transcription factors may play a major role in the Douglas-fir NECs undifferentiated cell proliferation which could appear questionable due to their known role as master regulators of organ identity, i.e., in ovule development in grapes (Li et al., 2017). Inclusion of a set of transcripts with “bHLH” annotation (Supplementary Figure S7), indicates involvement of basic helix-loop-helix motif transcription factors (Heim et al., 2003), which have pleiotropic effects in cell proliferation and differentiation. Five other transcripts have “iron deficiency-induced” transcription factor annotation and are functionally related to the bHLH family (e.g., Long et al., 2010). These results suggest that control of cell proliferation differs between NECs and EMs, which may be strongly linked to the difference in meristematic status (Figure 11 and discussion below). Indications of orientation toward cell divisions are much stronger in EMs (Figure 2) at the transcriptomic level, preceding production of functional proteins, but the cell cycling machinery seems to be oriented toward callus proliferation, which also involves mitosis, in NECs too. Thus, differences between meristematic status and callus production are very subtle in terms of cell division, and our use of EM and NEC isogenic lines enables valuable comparisons.

### Proteomic Profiles

Among the proteins significantly different between EMs and NECs (Figure 9 and Supplementary Figure S8), only 15.8% showed the same expression pattern in all three genotypes, which gave information about the variability among genotypes in protein expression. During the last 10 years, protein expression analysis has improved dramatically due to both technological and methodological advances (Heringer et al., 2018). This had direct consequences on the significant protein number, which were often less than 40 in embryogenic and non-embryogenic tissue comparison studies based on 2 dimension gels. The much higher number of significant proteins identified with free-gel-based studies, thus consolidates the hypotheses made on the BPs determining the embryogenic character of a tissue. We detected 2,619 significantly differentially expressed proteins in total, but there were substantial quantitative differences between genotypes (Supplementary Figure S8), and only 413 were significantly differentially expressed between EMs and NECs of all three genotypes. Similar variability has been observed in *Pinus nigra*, where PCA of significantly differentially expressed proteins revealed that genotype explained more of the observed variance than tissue type (Klubicová et al., 2017).

We analyzed our results by SNEA, a type of enrichment analysis based on protein interaction networks using daily updated bibliographic databases, connecting significantly differentially expressed proteins and BPs. Our results show that proteins more strongly expressed in EMs were associated with cell division and differentiation, embryonic development, protein synthesis and carbon metabolism, while those more strongly expressed in NECs were associated with defense and stress responses, and to a lesser extent primary (carbon and nitrate) metabolism (Figure 10).

Cell divisions are usually more frequent in embryogenic lines, which grow more rapidly than non-embryogenic lines. Accordingly, microscopic comparison revealed large numbers of mitotic meristematic cells in our EMs (Figure 2). Evidence of at least 13 BPs were detected in EMs, associated with cell division, cell polarization and embryonic development activity, in accordance with our morphological analyses and observations in other conifers (Jourdain et al., 1997; Zhao et al., 2015; Klubicová et al., 2017; Gautier et al., 2018). Other proteins upregulated in EM could also be related to these BPs, in addition to those identified in Figure 10. The protein activating GTPase factor ADP-ribosylation (AGD8) is involved, as well as ERMO2, in efflux of auxins and subsequent polar localization in cells (Heringer et al., 2018), expansin A11 causes loosening and extension of plants’ cell walls (Sampedro and Cosgrove, 2005) and proline-tRNA ligase C19C7.06 mediates cell elongation and polarization. The last of these is also reportedly upregulated in the non-embryogenic tissue of *Larix*, where cell multiplication is nonetheless activated (Zhao et al., 2015). Our transcriptomic results were similar (Figure 11), since only three transcripts corresponding to significantly differentially expressed proteins involved in these cell division activities were downregulated in the EMs (PSME_00000169-RA, PSME_00024028-RA and PSME_00033280-RA). Two corresponded to transporters (IMPA-1 and ELF5A) and one to transcript VLN2, encoding villin-2, which is involved in actin filament bundling. However, while ratios of the transcripts’ abundance in EMs and NECs were quite large, they were lower for the corresponding proteins. Such differences in transcriptomic and proteomic dynamics has been frequently described and explained in the literature (Li et al., 2015). However, actins (ACT7, ACT11), UDP-L-arabinose mutase 1 (RGP1), and Sec24-like protein transport protein (ERMO2) were among the most highly overexpressed actors at both transcript and protein levels. Alpha-1,4-glucan-protein synthase and UDP-glucose 6 dehydrogenase (both absent in Figure 7) as well as RGP1 are all involved in synthesis of cell wall polysaccharides, and have been found to be more abundant in embryogenic than in non-embryogenic tissue in *Larix* (Zhao et al., 2015) and *Pinus nigra* (Klubicová et al., 2017), or early stages of somatic embryogenesis (Yu et al., 2016). ERMO2 acts as a major regulator of vesicle trafficking, which affects cells’ polarization, maintenance of endoplasmic reticulum organization, and protein transport (Nakano et al., 2009).

The large number of BPs associated with numerous upregulated proteins and corresponding transcripts demonstrates the very strong orientation of EM tissues toward cell differentiation, which could be considered their main characteristic relative to NEC. This conclusion is new regarding the comparison studies between embryogenic and non-embryogenic tissues.

Active carbon and energy metabolism play key roles in embryogenicity by supporting cell division and modification. Hence, more than 30% of the significantly differentially expressed proteins detected in both this (Supplementary Table S4) and most previous proteomic comparisons are involved in these functional processes. Activation of these proteins associated with primary and energetic metabolism in EMs is consistent with the detected sets of differentially over-expressed transcripts related to metabolic processes. This could explain our findings regarding the energetic balance, as expressed by the (Glc + Fru)/Suc ratio, in EMs and the starch content in NECs (Table 1 and Figure 2). Nevertheless, while numerous upregulated enzymes in EMs are related to primary or energetic metabolism (alcohol dehydrogenase, enolase, fructokinase, inositol-3-phosphate synthase isoform X1, phosphoenolpyruvate carboxylase 4, phosphoglycerate kinase, UDP-arabinopyranose mutase 1, UDP-glucose 6-dehydrogenase 4, UDP-glucuronic acid decarboxylase), no connection was detected in the protein network with the BPs “gluconeogenesis,” “tricarboxylic acid cycle” and “glycolysis” (Figure 10). However, at transcriptomic level, no clear differences between EMs and NECs were detected by GSEA in MF GO terms associated with primary metabolism, whereas the BPs “gluconeogenesis” and “tricarboxylic acid cycle” seemed to be activated in EMs, as shown in Figure 11. We detected higher expression of enolase and fructokinase in EMs than in NECs, as previously observed in other species (Varhanikova et al., 2014; Chin and Tan, 2018).

Protein synthesis and processing play key roles in all cellular development processes, and thus differ between non-embryogenic and EM tissues (Amsterdam et al., 1993; Lyngved et al., 2008). We detected up-regulation in our Douglas-fir EMs of proteins involved in all of the protein processing steps. However, only some proteins mentioned below were found in the protein network (Figure 10), although others confirmed the identified BPs. This was also the case at the transcriptomic level (Figure 11). The involvement of DNA and RNA metabolism is confirmed by high abundance of proteins such as histones (histone deacetylase HDT2-like, histone-arginine methyltransferase 1.3), glycine-rich RNA-binding proteins (glycine-rich RNA-binding protein GRP1A-like (CCR1), glycine-rich RNA-binding (AtRZ-1c), ABA-inducible protein isoform X1), and proteins related to the resulting transport (alpha importin, putative, importin beta-like SAD2). For protein synthesis, numerous proteins were upregulated in EMs, including, 60S ribosomal protein isomers (60S ribosomal L4, L8, L12-like protein L15-1, L27a-3 and L35), as well as elongation factors (eukaryotic translation initiation factor 3 subunit A, eukaryotic translation initiation factor 3 subunit G and eukaryotic translation initiation factor 5A-2-like). At the transcript level, ribosome production also appeared to be strongly upregulated in Douglas-fir EMs. Among identified chaperones, which assist proteins’ folding, T-complex protein 1 was found to be more abundant in the embryogenic tissues of Douglas-fir, and *Pinus nigra* (Klubicová et al., 2017), but more abundant in non-embryogenic tissues of *Crocus sativus* (Sharifi et al., 2012). HSP70 is frequently identified in proteomic studies among chaperones associated with embryogenicity (Chin and Tan, 2018). This protein is still often considered a stress-related protein in proteomic studies, and the corresponding stress response process as necessary for embryogenic competence acquisition (Zhang et al., 2009; Sharifi et al., 2012; Kumaravel et al., 2017). Protein recycling is strongly involved in cell proliferation processes (Amsterdam et al., 1993), thus proteases, peptidases, and various other proteins associated with proteasomal degradation are often associated with embryogenic capacity (Sharifi et al., 2012) or early stages of embryogenesis (Lippert et al., 2005). Accordingly, proteins upregulated in our EMs included UBP1-associated protein 2B-like, ubiquitin-conjugating enzyme E2 7 (UBC13), 26S proteasome non-ATPase regulatory subunit 5 involved in proteasomal activity, and two isomers of aspartic proteinase nepenthesin involved in protease activity.

The importance of the number of proteins involved in protein metabolism, overexpressed in EM compared to NEC, suggest that these BPs are a key factor in embryogenic competence and somatic embryogenesis.

Defense responses against biotic or abiotic stress modulate embryogenic culture capacity or somatic embryogenesis (Silva Rde et al., 2014; Fehér, 2015; Li et al., 2015), and proteins associated with stress are generally upregulated in EMs (Trontin et al., 2016b). However, the protein network we obtained clearly indicates that defense responses against various stresses were induced more strongly in our NECs rather than EMs.

Cell cultures as well as plants develop against pathogen attack various reactions involving notably pathogenesis-related (PR) proteins (Edreva, 2005), including chitinases, glucanases, lysozyme-active proteins, that have been found in relation to cellular development. They may be more abundant in either embryogenic tissues (Helleboid et al., 2000; Silva Rde et al., 2014; Zhao et al., 2015), or non-embryogenic tissues (Marsoni et al., 2008; Zhang et al., 2009; Correia et al., 2012; Klubicová et al., 2017). Thus, relations of these proteins to embryogenic capacity are not clear, although only a few of them (1–3) have been examined in some previous studies. We found that several PR proteins were less strongly expressed in EMs than in NECs, including: endochitinase B (HCHIB), a class I chitinase that is reportedly upregulated in NECs of *Pinus nigra* (Klubicová et al., 2017); PR-4-like pathogenesis-related protein (PR4); pathogenesis-related protein bet VI family protein; and thaumatin-like protein 1. Peroxidase 12 (PRX52 and PRX72) due to their action in response to environmental stresses such as pathogen attack, belong to the PR protein family (Goncalves et al., 2013), and should be linked to a defense process in the protein network (Figure 10) and not to the cellular process of “lignification” as in *Arabidopsis thaliana*.

Reactive oxygen species are readily detectable in plants’ responses to abiotic and biotic stresses, which may be key triggers of somatic embryogenesis (Sharifi et al., 2012). Auxin generates ROS, thus contributing to its induction of embryogenesis. We found that endogenous auxin levels were higher in EMs than in NECs (Figure 4), suggesting activation of the ROS detoxification system and thus anti-oxidative proteins such as catalase, ascorbate peroxidase (APX1) and glutathione-*S*-transferase (ERD9). The abundance of these enzymes, is frequently higher in EM than in NEC tissues (Marsoni et al., 2008; Sharifi et al., 2012; Silva Rde et al., 2014), and it has even been regarded as a determinant of cultures’ embryogenic capacity. However, glutathione-*S*-transferase can also participate in cell signaling, catalase in sugar or amino acid metabolism and ascorbate peroxidase in the glutathione-ascorbate cycle, all of which were activated in embryogenic tissues. Thus, these enzymes may contribute to embryogenic capacity through various developmental and cell cycling processes rather than merely through deactivation of ROS. In addition, superoxide dismutase, also involved in oxidative-stress responses, has not generally been found to be more abundant in EMs than in NECs, except in palm oil trees (Silva Rde et al., 2014). In our Douglas-fir lines, these ROS detoxification systems did not appear to be activated more significantly in either EMs or NECs, since only two peroxidase ascorbate (APX) isoform were detected among the differentially expressed proteins, each of which was upregulated in one type of tissue, as found in *Vitis vinifera* (Zhang et al., 2009). In addition, previous authors have found higher abundances of these anti-oxidative enzymes in non-embryogenic tissues of other various species, including conifers, *Larix* (Zhao et al., 2015) *Pinus nigra* (Klubicová et al., 2017), and *Vitis vinifera* (Zhang et al., 2009). Thus at least in conifers, there is no confirmed link between abundance of these enzymes and embryogenic capacity.

These stress defense reactions have been interpreted as responses of the tissues to *in vitro* culture conditions (Teyssier et al., 2011; Correia et al., 2012; Morel et al., 2014; Zhao et al., 2015). They are probably not directly related to a specific morphogenic pathway such as somatic embryogenesis, but may be prerequisites for embryogenesis. We could partly explain our results by the culture of NECs and EMs on the same multiplication medium, which has been optimized for EM multiplication. It did not seem to be totally adapted to NECs, which showed signs of difficulties in these conditions: lower multiplication rates than the EMs (result not shown); substantially lower frequencies of dividing cells (Figure 2) contrasting with the important presence of mitotic centers observed in the EMs, enhancement of phenolic compounds (Figure 2); and signs of necrosis (data not shown). In *Pinus nigra*, Klubicová et al. (2017) found that all detected differentially expressed proteins involved in detoxification were more abundant in non-embryogenic than in embryogenic tissues, and related this pattern to the tissues’ predisposition to necrosis following oxidation of phenolic compounds. This could also apply to Douglas-fir NECs, which exhibited similar characteristics.

To summarize, in proteomic analysis the establishment of a network of interactions between significant proteins was proven very useful. In addition, our study confirms again the importance of working with modern proteomics techniques and with several unrelated genotypes to screen for proteins involved in embryogenic state.

## Conclusion

Numerous differences between EM and NEC of Douglas-fir were observed at the cytological, biochemical and molecular levels. Key characteristics of Douglas-fir EMs may include cell multiplication and differentiation of embryogenic tissue, supported by enhancement of energy metabolism and protein recycling machinery, while upregulation of stress responses may be more characteristic of NECs. In addition, numerous differences between EMs and NECs of the three genotypes indicate that auxin, bioactive and transport forms of isoprenoid and aromatic CKs, as well as isoprenoid CK nucleotides, may be markers of EM formation. In contrast, NECs were characterized by high levels of ABA, ABA-GE and glycosylated CKs (both isoprenoid and aromatic). Finally, the study illustrates the value of comprehensive multi-level comparisons of isogenic embryogenic and non-embryogenic lines, with advanced methodology. Our innovative application of network analysis also contributed to our results, which provide the first report providing integrated insights into cellular, biochemical and molecular events involved in embryogenesis in conifers, and more specifically cellular embryogenic state in Douglas-fir.

## Author Contributions

FG participated in the acquisition of all the data, as well as somatic embryogenesis, and transcriptomic and protein analysis. PL participated in design of the study and transcriptomic analysis. KE performed histological and microscopical analyses. J-CL participated in Sub-Network Enrichment analysis. VM analyzed phytohormone data. NB performed carbohydrate analysis. ZV carried out somatic embryogenesis. JM performed LC-MS phytohormone analysis. AT pre-treated samples for phytohormone analysis. CL carried out somatic embryogenesis and collected the material. M-CL-D helped in the transcriptomic analysis. A-ML carried out mass spectrometric analysis. J-FT participated in design of the study. GC participated in design of the study. CT participated in design of the study, protein and Sub-Network Enrichment analysis. M-AL-W participated in design of the study, coordinated it and participated in somatic embryogenesis. FG, PL, KE, J-CL, VM, NB, ZV, M-CL-D, A-ML, J-FT, GC, CT, and M-AL-W also contributed to writing of the manuscript, and all authors read and approved the final manuscript.

## Conflict of Interest Statement

The authors declare that the research was conducted in the absence of any commercial or financial relationships that could be construed as a potential conflict of interest.

## Footnotes

1 https://www.ncbi.nlm.nih.gov/bioproject/PRJNA491234

2 https://treegenesdb.org/FTP/Transcriptome/cDNA/Psme/Psme_cDNA.fasta

3 https://treegenesdb.org/FTP/Genomes/Psme/v1.0/annotation/

4 http://www.geneontology.org/

5 https://www.arabidopsis.org/

6 http://pantherdb.org/

7 https://www.bioconductor.org/

## Abbreviations

ABA: abscisic acid
ABA-GE: ABA-glucose ester
CKs: cytokinins
d.w.: dry weight
EM: embryonal mass
f.w.: fresh weight
GO: Gene Ontology
IAA: indole-3-acetic acid
LC-MS/MS: liquid chromatography-tandem mass spectrometry
NEC: non-embryogenic callus
ROS: reactive oxygen species
SE: somatic embryo
SNEA: Sub-Network Enrichment Analysis
