# Supplementary material for: Cytological, Biochemical and Molecular Events of the Embryogenic State in Douglas-fir (Pseudotsuga menziesii [Mirb.])
Source: Front Plant Sci. 2019 Feb 28;10:118. doi: 10.3389/fpls.2019.00118 (PMC6403139; doi:10.3389/fpls.2019.00118)
Supplement: Supplementary file 3 [file Image_1.pdf]

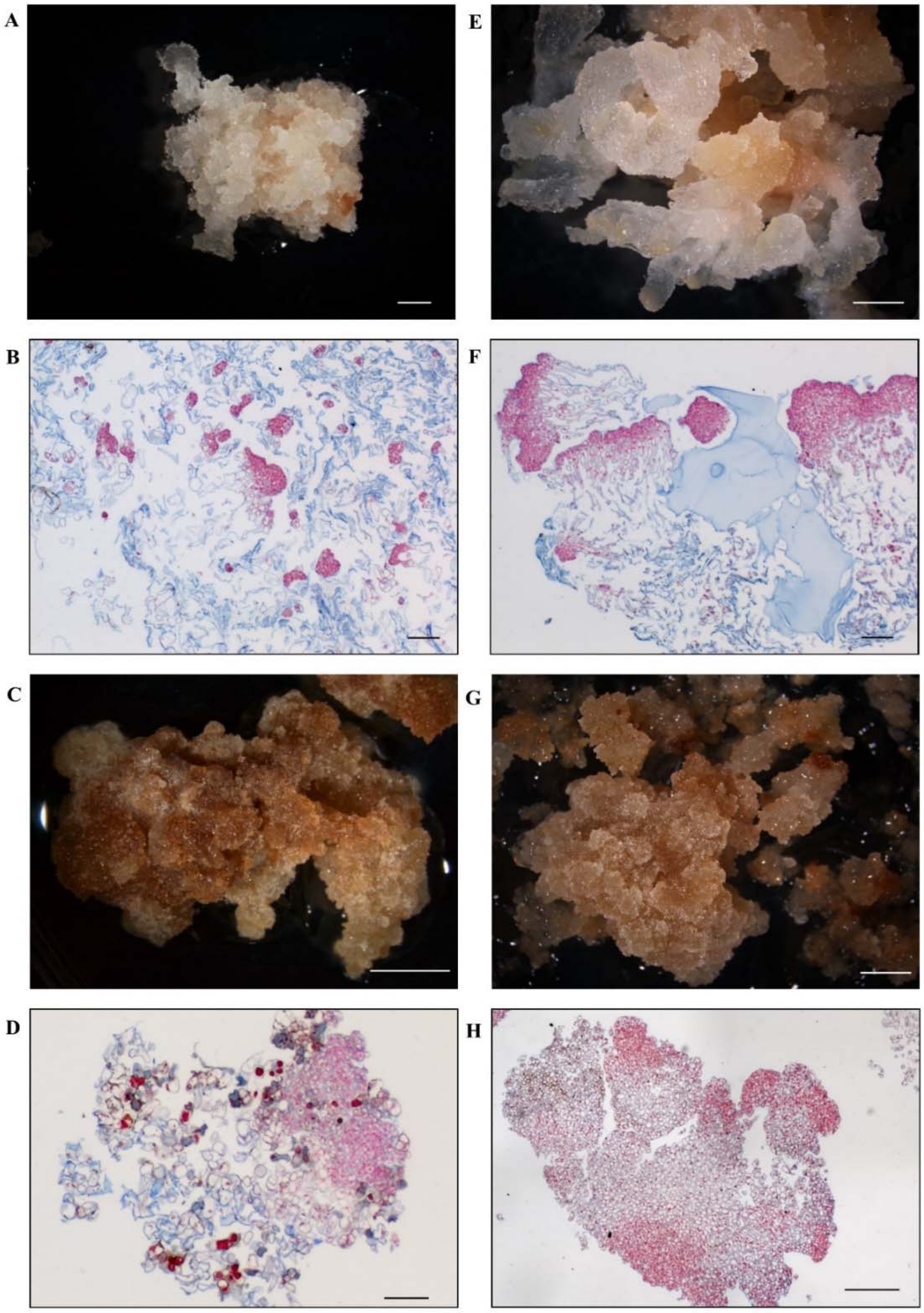

Supplementary Figure S1. Morphological and histological characterization of isogenic embryonal mass (EM) and non-embryogenic callus (NEC) of Douglas-fir.

(**A**) Morphology of SD4-8 EM, (**B**) Small somatic embryos of SD4-8 EM; paraffin section stained with Nuclear Fast Red/Alcian Blue; (**C**) Morphology of SD4-8 NEC; (**D**) Histology of SD4-8 NEC; note the cells accumulating phenolic compounds (in grey-blue, brownish or amber colour); (**E**) morphology of TD17-1 EM; (**F**) Polyembryogenic centres of TD17-1 EM; (**G**) morphology of TD17-1 NEC, (**H**) Histology of TD15-1 NEC. Scale bars represent: 2 mm (A, E); 500 µm (C, H); 200 µm (B, D, F).
