# Supplementary material for: Cytological, Biochemical and Molecular Events of the Embryogenic State in Douglas-fir (Pseudotsuga menziesii [Mirb.])
Source: Front Plant Sci. 2019 Feb 28;10:118. doi: 10.3389/fpls.2019.00118 (PMC6403139; doi:10.3389/fpls.2019.00118)
Supplement: Supplementary file 4 [file Image_2.pdf]

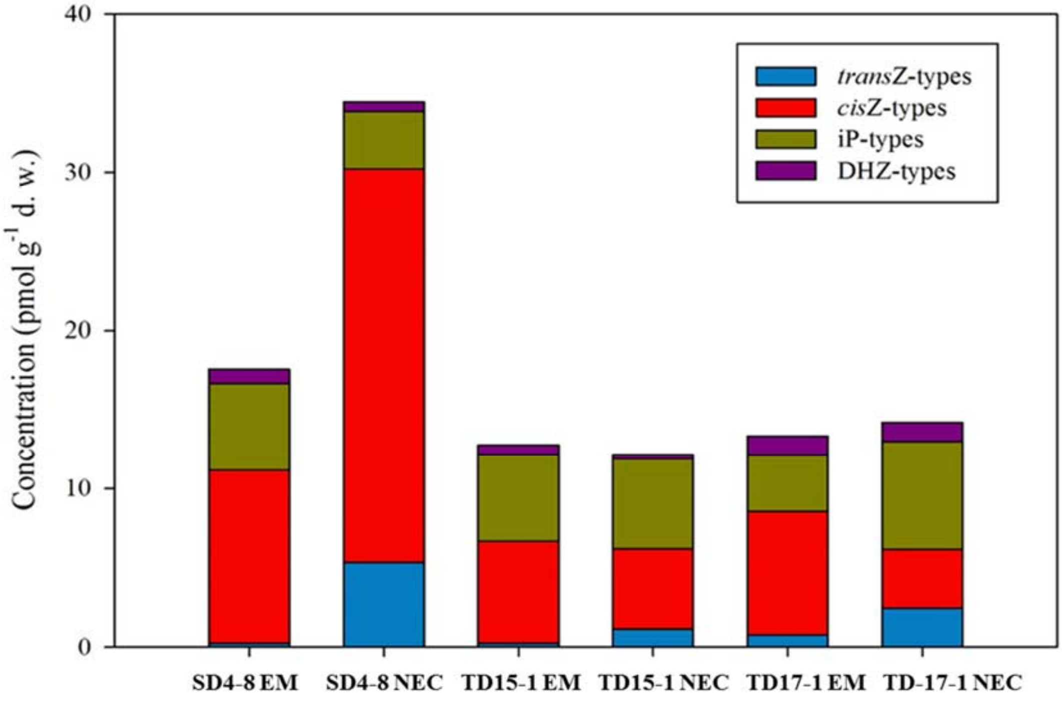

Supplementary Figure S2. Summary of isoprenoid cytokinin (CK) types concentrations in isogenic embryonal mass (EM) and non-embryogenic callus (NEC) of three genotypes (SD4-8, TD15-1, TD17-1) of Douglas-fir during the proliferation phase of somatic embryogenesis.
The following isoprenoid CK derivatives were detected:
trans-zeatin types: *trans*-zeatin 9-riboside, *trans*-zeatin *O*-glucoside, *trans*-zeatin-*N*7-glucoside
cis-zeatin types: *cis*-zeatin 9-riboside, *cis*-zeatin 9-riboside-5'-monophosphate, *cis*-zeatin 9-riboside *O*-glucoside, *cis*-zeatin-*N*7-glucoside
N^6-(Δ^2-isopentenyl)adenine types: N^6-(Δ^2-isopentenyl)adenine, N^6-(Δ^2-isopentenyl)adenosine, N^6-(Δ^2-isopentenyl)adenosine-5'-monophosphate
dihydrozeatin types: dihydrozeatin 9-riboside-5'-monophosphate, dihydrozeatin-*N*7-glucoside.
