# Supplementary material for: Cytological, Biochemical and Molecular Events of the Embryogenic State in Douglas-fir (Pseudotsuga menziesii [Mirb.])
Source: Front Plant Sci. 2019 Feb 28;10:118. doi: 10.3389/fpls.2019.00118 (PMC6403139; doi:10.3389/fpls.2019.00118)
Supplement: Supplementary file 5 [file Image_3.pdf]

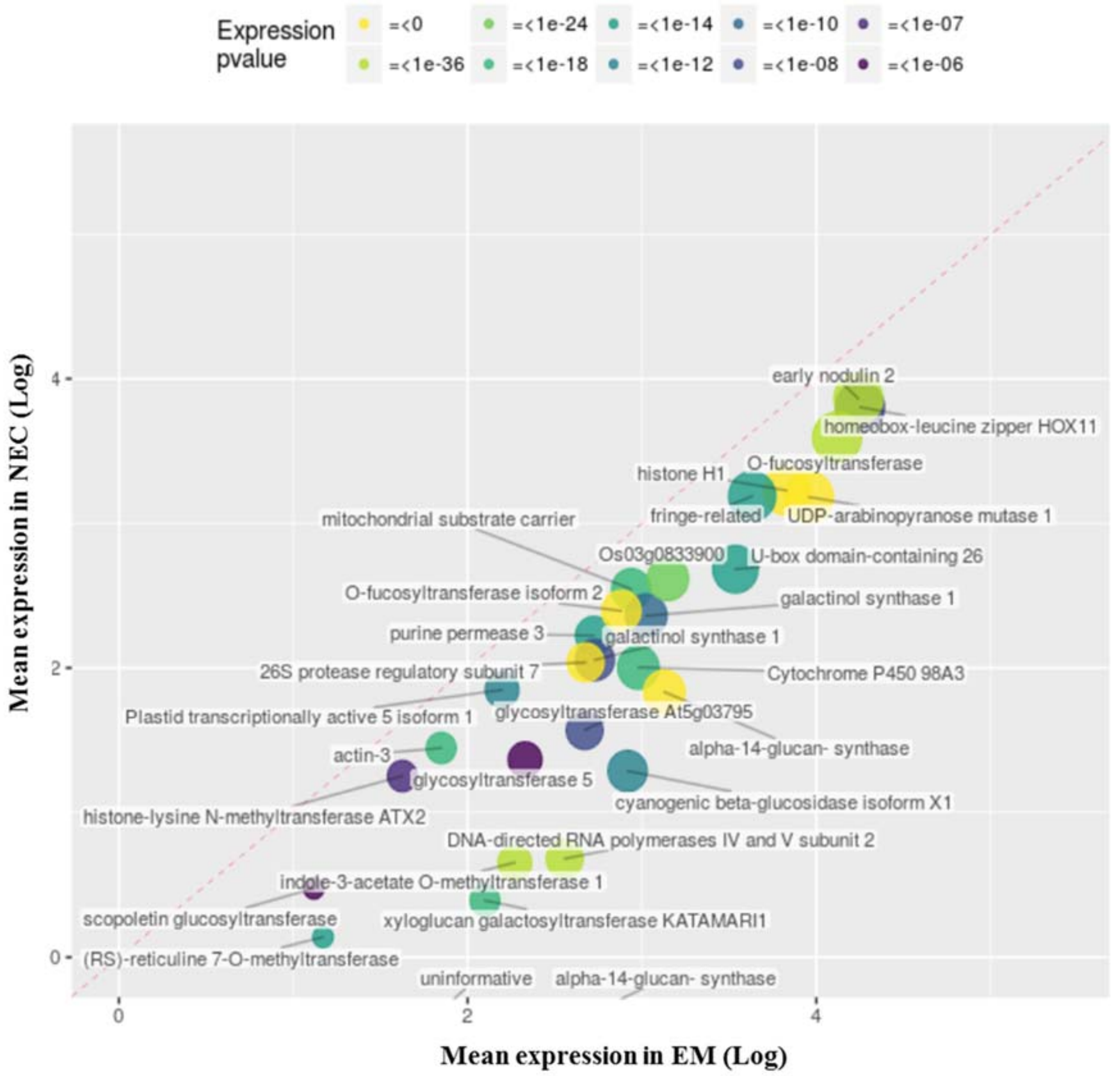

Supplementary Figure S3. Transcripts up-regulated in embryonal mass (EM) relative to non-embryogenic callus (NEC) annotated to MF GO:0016757, "transferase activity, transferring glycosyl groups". See legend of **Figure 6** for further information.
