# Supplementary material for: Cytological, Biochemical and Molecular Events of the Embryogenic State in Douglas-fir (Pseudotsuga menziesii [Mirb.])
Source: Front Plant Sci. 2019 Feb 28;10:118. doi: 10.3389/fpls.2019.00118 (PMC6403139; doi:10.3389/fpls.2019.00118)
Supplement: Supplementary file 6 [file Image_4.pdf]

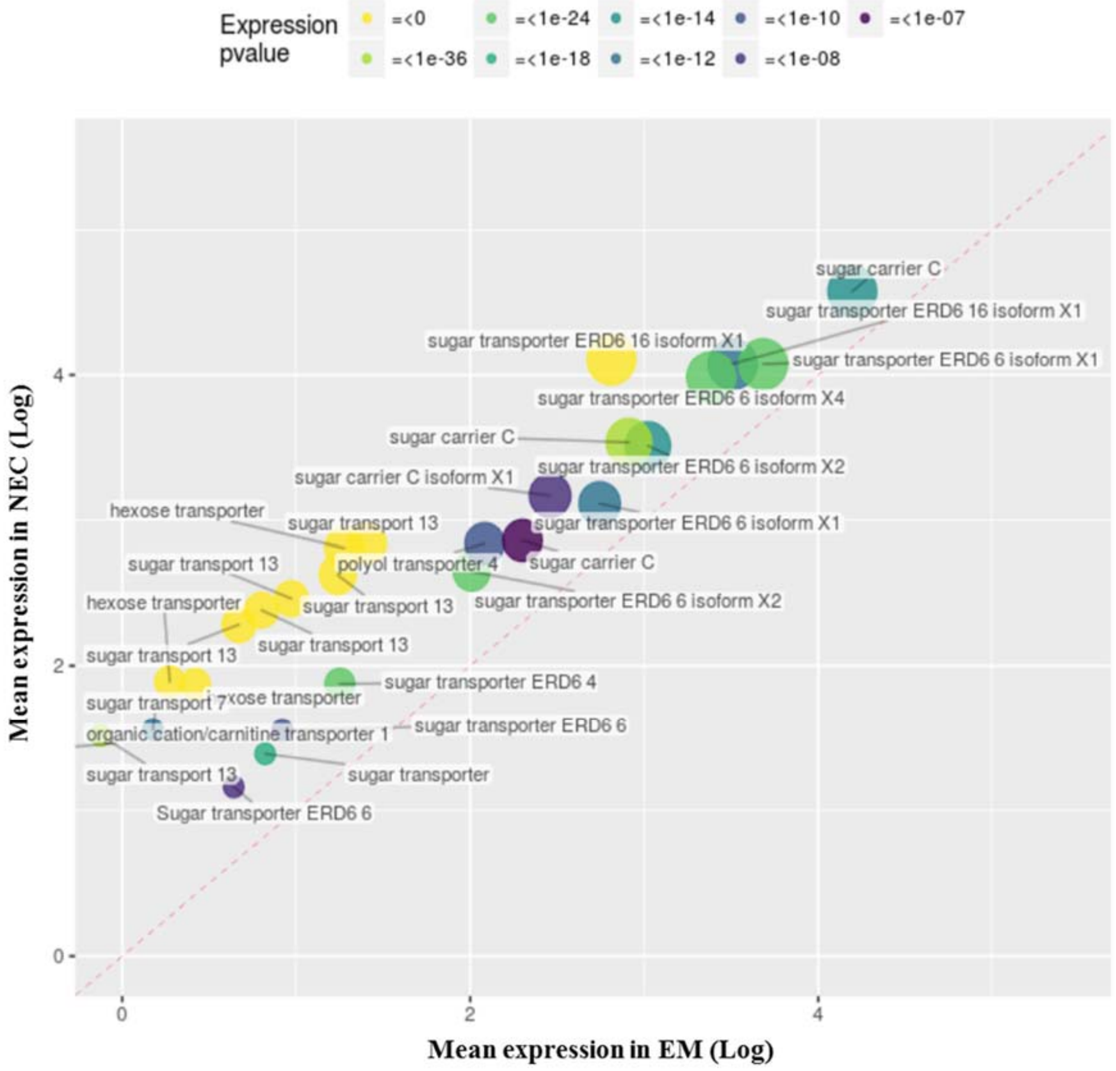

Supplementary Figure S4. Transcripts up-regulated in non-embryogenic callus (NEC) relative to embryonal mass (EM) annotated to MF GO:0022857, “transmembrane transporter activity”. See legend of **Figure 7** for further information.
