# Supplementary material for: Cytological, Biochemical and Molecular Events of the Embryogenic State in Douglas-fir (Pseudotsuga menziesii [Mirb.])
Source: Front Plant Sci. 2019 Feb 28;10:118. doi: 10.3389/fpls.2019.00118 (PMC6403139; doi:10.3389/fpls.2019.00118)
Supplement: Supplementary file 10 [file Image_8.pdf]

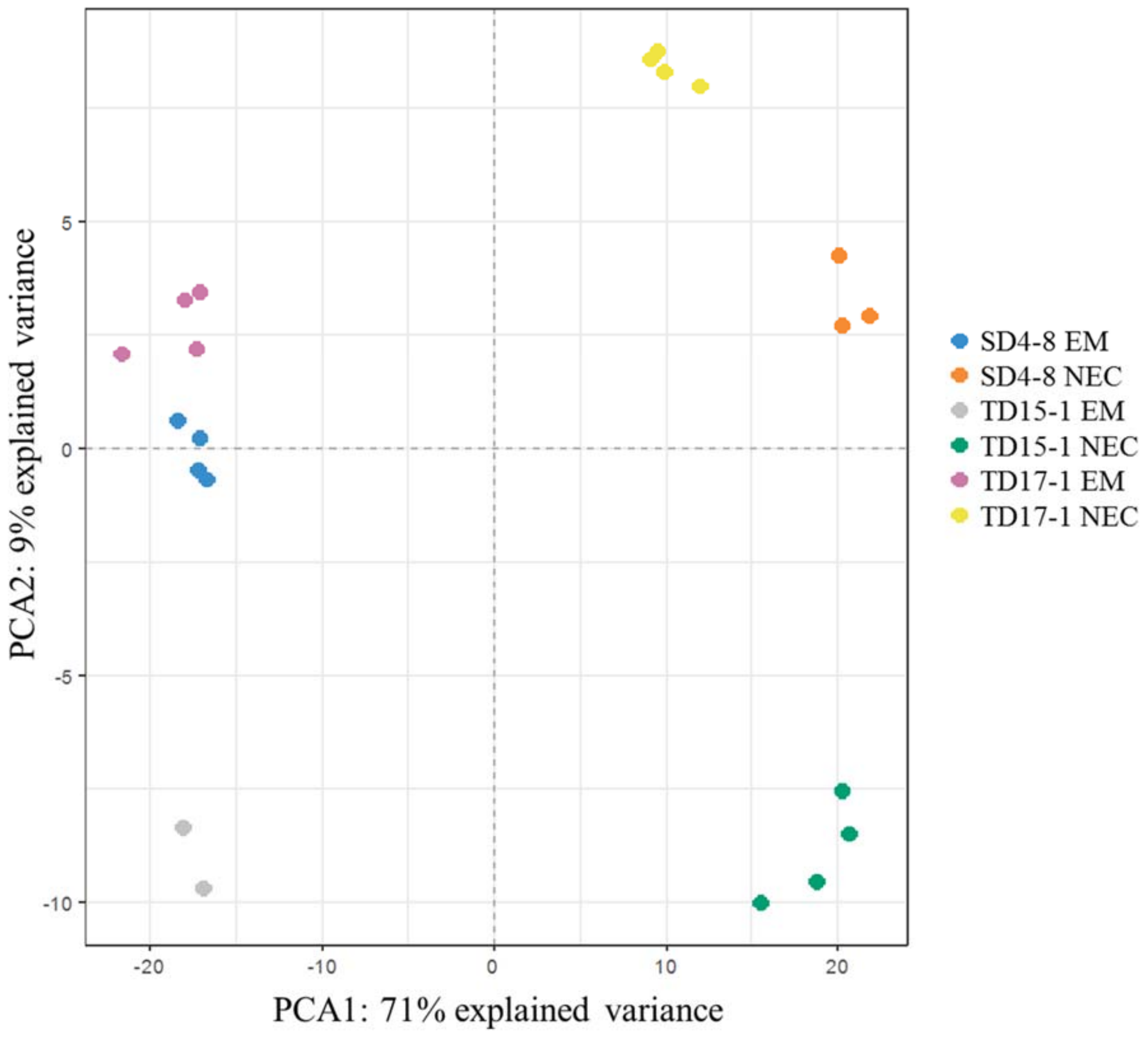


**Supplementary Figure S8**: Score plot obtained from Principal Component Analysis of proteomic profiles of isogenic embryonal mass (EM) and non-embryogenic callus (NEC) of three genotypes (SD4-8, TD15-1, TD17-1) of Douglas-fir during the proliferation phase of somatic embryogenesis based on 413 identified significantly differentially expressed proteins.
