# Supplementary material for: Cytological, Biochemical and Molecular Events of the Embryogenic State in Douglas-fir (Pseudotsuga menziesii [Mirb.])
Source: Front Plant Sci. 2019 Feb 28;10:118. doi: 10.3389/fpls.2019.00118 (PMC6403139; doi:10.3389/fpls.2019.00118)
Supplement: Supplementary file 11 [file Table_1.pdf]

Supplementary Table S1 Soluble sugar and starch contents of isogenic embryonal mass (EM) and non-embryogenic callus (NEC) of three genotypes (SD4-8, TD15-1, TD17-1) of Douglas-fir during the proliferation phase of somatic embryogenesis.

	SD4-8 EM	SD4-8 NEC	TD15-1 EM	TD15-1 NEC	TD17-1 EM	TD17-1 NEC
**Carbohydrate** (µg g^{-1} d.w.)						
**fructose**	18.9 ± 0.63*	3.87 ± 0.14*	17.5 ± 3.27	4.53 ± 0.46	15.0 ± 1.75	23.9 ± 7.92
**galactose**	2.22 ± 0.17	0 ±	3.31 ± 0.46	0	2.50 ± 0.30	0
**glucose**	209.9 ± 12.5*	13.6 ± 0.70*	203.3 ± 25.3*	78.6 ± 10.0*	211.9 ± 13.1	180.1 ± 15.1
**maltose**	0 ±	3.57 ± 0.12	6.09 ± 0.38	15.5 ± 13.8	0	7.74 ± 1.23
**melibiose**	4.06 ± 0.29	0 ±	2.83 ± 0.31	0	3.49 ± 0.43	0
**myo-inositol**	3.38 ± 0.23	3.53 ± 0.13	4.06 ± 0.27	4.56 ± 0.52	2.46 ± 0.23	4.01 ± 0.26
**raffinose**	16.9 ± 1.49*	1.91 ± 0.13*	6.88 ± 0.88	6.62 ± 3.64	10.9 ± 2.00	7.48 ± 0.86
**sucrose**	59.4 ± 2.24*	17.3 ± 2.31*	18.4 ± 4.01	21.1 ± 4.54	45.8 ± 6.23	42.1 ± 3.25
**starch** (mg g^{-1} d.w.)	10.2* ± 0.21*	19.3 ± 2.44*	2.56 ± 0.37*	8.69 ± 2.42*	14.9 ± 1.76*	26.8 ± 0.66*

Values are means of four measurements ± CI 5% error level. Asterisks indicate significant differences between EM and NEC lines obtained from multiple comparison of means ($P < 0.05$).
