# Supplementary material for: Cytological, Biochemical and Molecular Events of the Embryogenic State in Douglas-fir (Pseudotsuga menziesii [Mirb.])
Source: Front Plant Sci. 2019 Feb 28;10:118. doi: 10.3389/fpls.2019.00118 (PMC6403139; doi:10.3389/fpls.2019.00118)
Supplement: Supplementary file 12 [file Table_2.pdf]

Supplementary Table S2. Concentrations of endogenous phytohormones in isogenic embryonal mass (EM) and non-embryogenic callus (NEC) of three genotypes (SD4-8, TD15-1, TD17-1) of Douglas fir after two weeks of proliferation.

Phytohormones* (pmol.g^{-1} d.w.)	EM			NEC		
	**SD4-8**	**TD15-1**	**TD17-1**	**SD4-8**	**TD15-1**	**TD17-1**
**IAA**	301.45±11.69^{a}	241.81±60.62^{a}	246.90±32.42^{a}	266.58±30.44^{a}	110.15±8.93^{a}	153.57±43.04^{a}
**ABA**	176.60±20.76^{a}	139.40±51.69^{a}	513.91±297.39^{a}	4013.45±1481.83^{b}	756.49±283.88^{b}	1927.8±1187.97^{a}
**ABA-GE**	329.67±76.31^{a}	33.58±8.26^{a}	128.73±53.58^{a}	37055.47±6038.00^{b}	1401.17±309.58^{b}	6437.11±4683.65^{b}
**Isoprenoid CKs**						
*trans*Z	0	0	0	0	0	0
*trans*ZR	0	0.12±0.06	0.28±0.06	0	0	0
*trans*ZRMP	0	0	0	0	0	0
*trans*ZROG	0	0		0	0	0
*trans*ZOG	0.23±0.03	0.12±0.06	0.50±0.16	1.77±0.94	0.56±0.18	1.81±0.51
*trans*Z7G	0	0	0	3.52±0.14	0.58±0.28	0.63±0.37
*trans*Z9G	0	0	0	0	0	0
**Total *trans*Z-types**	**0.23**	**0.24**	**0.78**	**5.3**	**1.15**	**2.44**
*cis*Z	0	0	0	0	0	0
*cis*ZR	4.01±0.33	3.95±0.37	4.07±0.44	7.00±0.64	2.63±0.58	1.74±1.03
*cis*ZRMP	6.94±0.79	2.49±0.80	3.71±0.59	5.23±1.64	1.97±1.08	1.07±0.53
*cis*ZROG	0	0	0	9.21±0.68	0.42±0.14	0
*cis*ZOG	0	0	0	0	0	0
*cis*Z7G	0	0	0	3.48±1.06	0	0.89±0.51
*cis*Z9G	0	0	0	0	0	0
**Total *cis*Z-types**	**10.94**	**6.44**	**7.78**	**24.93**	**5.01**	**3.71**
iP	1.50±0.66	0.85±0.22	1.10±0.07	1.67±0.18	0.64±0.28	2.98±2.16
iPR	1.00±0.15	1.52±0.48	0.91±0.12	0	0.87±0.22	0.82±0.38
iPRMP	2.98±0.60	3.11±0.89	1.55±0.46	1.99±0.75	4.22±0.34	3.03±0.49
iP7G	0	0	0	0	0	0
iP9G	0	0	0	0	0	0
**Total iP-types**	**5.48**	**5.49**	**3.56**	**3.66**	**5.73**	**6.82**
DHZ	0	0	0	0	0	0
DHZR	0	0	0	0	0	0
DHZRMP	0.53±0.10	0.58±0.39	0.64±0.10	0.59±0.49	0.25±0.07	0.58±0.27
DHZROG	0	0	0	0	0	0
DHZOG	0	0	0	0	0	0
DHZ7G	0.36±0.09	0	0.58±0.09	0	0	0.64±0.13
DHZ9G	0	0	0	0	0	0
**Total DHZ-types**	**0.89**	**0.58**	**1.22**	**0.59**	**0.25**	**1.23**
**∑ Isoprenoid CKs**	**17.54**	**12.74**	**13.34**	**34.48**	**12.14**	**14.21**
**Aromatic CKs**						
BA	5486.1±434.2^{a}	6716.79±395.41^{a}	4853.41±352.34^{a}	27048.7±11899.1^{b}	7674.02±1894.03^{a}	3086.45±622.12^{b}
BAR	176.3±28.16^{a}	172.23±20.11^{a}	142.63±11.96^{a}	530.05±110.04^{b}	1729.5±277.07^{b}	133.69±27.25^{a}
BARMP	350.66±62.58^{a}	379.40±46.86^{a}	298.26±49.00^{a}	1655.14±72.27^{b}	5187.6±1132.87^{b}	375.69±96.79^{a}
BA3G + BA7G	6.19±1.84^{a}	7.83±2.27^{a}	6.54±2.63^{a}	221.97±41.53^{b}	22.14±3.90^{b}	20.46±2.4^{b}
BA9G	11.09±1.12^{a}	4.55±1.17^{a}	3.73±1.75^{a}	1578.89±214.41^{b}	117.49±20.39^{b}	46.99±31.88^{a}
**Total BA-types**	**6030.33**	**7280.8**	**5304.57**	**31034.76**	**14730.75**	**3663.28**
OH-BA	5.07±0.11	4.84±0.85	11.75±7.35	69.08±25.59	28.28±3.72	3.63±1.80
OH-BAR	0.17±0.10	0.90±0.12	0.49±0.1	16.18±5.2	4.13±0.67	0.51±0.33
OH-BARMP	0.87±0.24	7.32±3.59	3.03±0.72	4.6±0.63	11.26±1.13	1.35±0.78
OH-BA9G	0	0	0	1.68±0.73	0.43±0.65	0
**Total OH-BA derivatives**	**6.12**	**13.06**	**15.27**	**91.54**	**44.10**	**5.49**

All data are means ± CI. Different letters indicate significant differences according to multiple comparisons of means ($P < 0.05$, n=3-4).

*IAA, indole 3-acetic acid; ABA, abscisic acid, ABA-GE, ABA-glucose ester;

Isoprenoid cytokinins (CKs) included *trans*-zeatin (*trans*Z), *cis*-zeatin (*cis*Z), N^6-(Δ^2-isopentenyl)adenine (iP) and dihydrozeatin (DHZ) types;

Aromatic CKs included *N*[6]-benzyladenine (BA) types, including derivatives hydroxylated on the side-chain phenyl ring in *ortho*, *meta* and *para* positions (OH-BA types).
Abbreviations of CKs adopted and modified according to Kamínek et al. (2000).
