# Supplementary material for: Cytological, Biochemical and Molecular Events of the Embryogenic State in Douglas-fir (Pseudotsuga menziesii [Mirb.])
Source: Front Plant Sci. 2019 Feb 28;10:118. doi: 10.3389/fpls.2019.00118 (PMC6403139; doi:10.3389/fpls.2019.00118)
Supplement: Supplementary file 13 [file Table_3.pdf]

**Supplementary Table S3**. Up-regulation of 96 Douglas transcripts set in EM grouped under 'GO:0003723' (pvalue = $2.4e^{-24}$, from TopGO) MF ontology. Douglas transcript identification (ID_Douglas) is from Douglas reference transcriptome (Neale's Laboratory, UC Davis). Functional annotation (Function), *Arabidopsis thaliana* orthology (At_orthologs) and GO ontologies (MF, molecular function ; BP, biological process ; CC, cellular component) were obtained from Blast2GO. pvalue : FDR adjusted pvalue for differential expression obtained from DESeq2. mean mapping score and standard errors, ME, NE and ±se are provided for EM (n=16) and NEC (n = 21) respectively. FC : fold-change. Expr : trend of expression relative to fold-change. Mapping scores are rounded to integer.

| ID_Douglas | Function | At_ortholog | MF | BP | CC | Expr. | FC | pvalue | EM | NEC |
|---|---|---|---|---|---|---|---|---|---|---|
| PSME_00000323-RA | Os09g0400100 | AT4G39330.1 | GO:0098519 GO:0016887 GO:0016787 GO:0016740 GO:0016301 GO:0009378 GO:0008134 GO:0005525 GO:0005524 GO:0003924 GO:0003724 GO:0003723 GO:0003677 GO:0003676 GO:0000166 | GO:0007264 GO:0006810 GO:0006777 GO:0006614 GO:0006576 GO:0006355 GO:0006310 GO:0006281 GO:0002949 | | EM | 276,1 | $4e^{-28}$ | 4612 ±1192 | 13 ±3 |
| PSME_00000476-RA | ATP-dependent chaperone ClpB | AT1G74310.1 | GO:0098519 GO:0016887 GO:0016787 GO:0016301 GO:0009378 GO:0008134 GO:0005525 GO:0005524 GO:0003924 GO:0003724 GO:0003723 GO:0003677 | GO:0019538 GO:0007264 GO:0007155 GO:0006914 GO:0006810 GO:0006614 GO:0006355 GO:0006310 GO:0006281 GO:0002949 | | NE | 4,1 | $1e^{-24}$ | 2387 ±223 | 9754 ±813 |
| PSME_00000477-RA | chaperone ClpB1 | AT1G74310.1 | GO:0098519 GO:0016887 GO:0016787 GO:0016301 GO:0009378 GO:0008134 GO:0005525 GO:0005524 GO:0003924 GO:0003724 GO:0003723 GO:0003677 | GO:0019538 GO:0008608 GO:0007264 GO:0007155 GO:0006914 GO:0006810 GO:0006614 GO:0006576 GO:0006355 GO:0006310 GO:0006281 GO:0002949 | GO:0042729 GO:0005876 | NE | 3,5 | $2e^{-22}$ | 1427 ±129 | 5029 ±410 |
| PSME_00000852-RA | LRR receptor serine/threonine- kinase ERL1 | AT5G62230.1 | GO:0016887 GO:0009378 GO:0008134 GO:0005524 GO:0003724 GO:0003723 GO:0003678 | GO:0006355 GO:0006310 GO:0006281 GO:0002949 | | EM | 17,7 | $4e^{-07}$ | 786 ±93 | 38 ±13 |
| PSME_00001272-RA | arginine/serine-rich-splicing factor RSP41 isoform X1 | AT3G61860.1 | GO:0046872 GO:0004535 GO:0003723 GO:0003676 | GO:0006402 | GO:0005737 GO:0005634 | NE | 9,4 | $5e^{-20}$ | 313 ±71 | 3012 ±309 |
| PSME_00001740-RA | BREAST CANCER SUSCEPTIBILITY 1 homolog | AT1G04020.1 | GO:0016887 GO:0009378 GO:0005524 GO:0003724 GO:0003723 | GO:0006310 GO:0006281 | | EM | 2,6 | $9e^{-14}$ | 125 ±8 | 47 ±5 |
| PSME_00001938-RA | ABC transporter B member 9 | AT2G47000.1 | GO:0042626 GO:0031683 GO:0019001 GO:0016887 GO:0016301 GO:0008134 GO:0005525 GO:0005524 GO:0004871 GO:0003924 GO:0003860 GO:0003724 GO:0003723 | GO:0055085 GO:0008152 GO:0007186 GO:0006810 GO:0006355 | GO:0016021 | NE | 4,8 | $7e^{-07}$ | 30 ±5 | 151 ±24 |
| PSME_00002021-RA | serine carboxypeptidase 45 | AT1G28110.1 | GO:0015093 GO:0005525 GO:0003924 GO:0003724 GO:0003723 | GO:0015684 | GO:0016021 | EM | 7,5 | $1e^{-23}$ | 11566 ±2253 | 1526 ±141 |
| PSME_00002394-RA | galactinol synthase 1 | AT2G47180.1 | GO:0016887 GO:0005525 GO:0005524 GO:0003924 GO:0003724 GO:0003723 | | GO:0016020 | EM | 4,2 | $4e^{-07}$ | 242 ±34 | 56 ±9 |
| PSME_00002564-RA | glucan 13-beta-glucosidase A | | GO:0003723 | | | EM | 24,5 | $9e^{-07}$ | 419 ±114 | 13 ±6 |
| PSME_00002668-RA | cytokinin dehydrogenase 1 | AT3G63440.1 | GO:0019901 GO:0005515 GO:0003723 | GO:0016070 GO:0010508 GO:0007030 GO:0006914 | | EM | 16,9 | $2e^{-14}$ | 378 ±62 | 21 ±5 |
| PSME_00004238-RA | Coatomer subunit beta-1 | AT1G59870.1 | GO:0098519 GO:0016887 GO:0016301 GO:0009378 GO:0005525 GO:0005524 GO:0003924 GO:0003724 GO:0003723 | GO:0007264 GO:0006777 GO:0006576 GO:0006310 GO:0006281 GO:0002949 | GO:0016020 | NE | 11 | $2e^{-06}$ | 2 ±1 | 27 ±5 |

| | | | | | | | | | | |
|---|---|---|---|---|---|---|---|---|---|---|
| PSME_00004494-RA | transcription repressor MYB5 | AT1G22640.1 | GO:0098519 GO:0016887 GO:0005525 GO:0005524 GO:0004127 GO:0003924 GO:0003724 GO:0003723 | GO:0006139 | GO:0016020 | EM | 18,6 | 8e$^{-09}$ | 206 ±34 | 10 ±3 |
| PSME_00004747-RA | APO 3 mitochondrial | AT5G61930.1 | GO:0003723 | | | EM | 2,2 | 1e$^{-08}$ | 171 ±15 | 79 ±7 |
| PSME_00005341-RA | chaperone ClpB1 | AT1G74310.1 | GO:0098519 GO:0016887 GO:0016787 GO:0016301 GO:0009378 GO:0008134 GO:0005525 GO:0005524 GO:0003924 GO:0003724 GO:0003723 GO:0003677 | GO:0019538 GO:0008608 GO:0007264 GO:0007155 GO:0006914 GO:0006810 GO:0006614 GO:0006355 GO:0006310 GO:0006281 GO:0002949 | GO:0042729 GO:0005876 | NE | 2,9 | 6e$^{-19}$ | 4275 ±451 | 12236 ±523 |
| PSME_00007886-RA | PHD finger MALE STERILITY 1 | AT5G22260.1 | GO:0003723 | | | EM | 4,4 | 1e$^{-04}$ | 22 ±4 | 5 ±1 |
| PSME_00007978-RA | Germin sub 1 member 1 | AT1G02335.1 | GO:0016887 GO:0009378 GO:0005524 GO:0003724 GO:0003723 GO:0003678 GO:0003677 | GO:0006310 GO:0006281 | | EM | 6,6 | 2e$^{-05}$ | 76 ±11 | 11 ±3 |
| PSME_00008177-RA | blue copper | AT1G08500.1 | GO:0016887 GO:0009378 GO:0005524 GO:0005126 GO:0003724 GO:0003723 | GO:0019079 GO:0006955 GO:0006310 GO:0006281 GO:0002949 | GO:0005576 | EM | 3,4 | 2e$^{-31}$ | 2617 ±154 | 764 ±58 |
| PSME_00008587-RA | abscisic stress-ripening 2 | | GO:0003723 | | | EM | 50,6 | 5e$^{-36}$ | 794 ±145 | 15 ±3 |
| PSME_00009075-RA | 7-deoxyloganetin glucosyltransferase | AT1G22400.1 | GO:0004525 GO:0003723 | GO:0006396 | | EM | 2,5 | 7e$^{-06}$ | 91 ±8 | 36 ±6 |
| PSME_00009405-RA | Sulfate/thiosulfate import ATP-binding cysA | AT3G07350.1 | GO:0003723 GO:0003676 | | | EM | 6,6 | 2e$^{-11}$ | 1450 ±129 | 216 ±37 |
| PSME_00009681-RA | : S-acyltransferase 21 | AT4G15080.1 | GO:0008168 GO:0003723 | GO:0008033 GO:0006364 | | EM | 3,2 | 1e$^{-25}$ | 848 ±41 | 260 ±26 |
| PSME_00009725-RA | multidrug resistance 1 2 | AT1G02520.1 | GO:0042626 GO:0031683 GO:0019001 GO:0016887 GO:0016301 GO:0008134 GO:0005525 GO:0005524 GO:0004871 GO:0003924 GO:0003724 GO:0003723 GO:0003676 | GO:0055085 GO:0008152 GO:0007264 GO:0007186 GO:0006810 GO:0006355 | GO:0016021 | NE | 3 | 4e$^{-27}$ | 387 ±18 | 1184 ±87 |
| PSME_00010934-RA | peptidyl-prolyl cis-trans isomerase CYP40 | AT2G15790.1 | GO:0098519 GO:0043531 GO:0016887 GO:0016787 GO:0016740 GO:0016301 GO:0009378 GO:0008134 GO:0005524 GO:0004127 GO:0003724 GO:0003723 GO:0003678 GO:0003677 | GO:0019079 GO:0006810 GO:0006576 GO:0006355 GO:0006310 GO:0006281 GO:0006139 GO:0002949 | | EM | 4 | 1e$^{-47}$ | 12492 ±265 | 3149 ±244 |
| PSME_00011755-RA | ATP binding | AT4G25835.1 | GO:0016887 GO:0009378 GO:0005524 GO:0003724 GO:0003723 | GO:0006310 GO:0006281 | | NE | 7,2 | 7e$^{-29}$ | 687 ±92 | 5011 ±481 |
| PSME_00011756-RA | ATP binding | AT4G25835.1 | GO:0016887 GO:0009378 GO:0005524 GO:0003724 GO:0003723 | GO:0006310 GO:0006281 | | NE | 4,4 | 5e$^{-11}$ | 326 ±68 | 1447 ±132 |
| PSME_00011757-RA | mitochondrial chaperone BCS1 | AT4G25835.1 | GO:0016887 GO:0009378 GO:0005524 GO:0003724 GO:0003723 | GO:0006310 GO:0006281 | | NE | 4,5 | 3e$^{-11}$ | 486 ±101 | 2239 ±225 |
| PSME_00011996-RA | anthocyanidin reductase | AT1G61720.1 | GO:0050662 GO:0016616 GO:0016301 GO:0005524 GO:0003854 GO:0003824 GO:0003723 | GO:0055114 GO:0006694 GO:0006353 | | NE | 11,4 | 6e$^{-24}$ | 17 ±4 | 191 ±23 |
| PSME_00012081-RA | kinesin NACK2 isoform X2 | AT1G18370.1 | GO:0016887 GO:0009378 GO:0008134 GO:0005524 GO:0003724 GO:0003723 GO:0003677 | GO:0006810 GO:0006576 GO:0006355 GO:0006310 GO:0006281 GO:0006260 GO:0002949 | | EM | 78,2 | 2e$^{-20}$ | 30 ±7 | 0 ±0 |
| PSME_00012402-RA | ice binding | AT1G65450.1 | GO:0003723 GO:0003676 | | | EM | 31,9 | 1e$^{-30}$ | 147 ±21 | 4 ±1 |

| | | | | | | | | | | |
|---|---|---|---|---|---|---|---|---|---|---|
| PSME_00012550-RA | cell division cycle 48 homolog | AT5G03340.1 | GO:0033897 GO:0003735 GO:0003723 | GO:0006412 | GO:0005840 GO:0005622 | EM | 2,1 | $6e^{-07}$ | 96 ±9 | 46 ±4 |
| PSME_00012870-RA | Coatomer subunit beta-1 | AT1G15520.1 | GO:0098519 GO:0016887 GO:0016301 GO:0009378 GO:0005525 GO:0005524 GO:0003924 GO:0003724 GO:0003723 | GO:0007264 GO:0006614 GO:0006576 GO:0006310 GO:0006281 GO:0002949 | GO:0016020 | NE | 44,6 | $1e^{-39}$ | 740 ±197 | 34811 ±6556 |
| PSME_00013088-RA | UDP-glycosyltransferase 86A1 | AT2G36970.1 | GO:0009982 GO:0003723 | GO:0009451 GO:0001522 | | EM | 5,7 | $1e^{-05}$ | 770 ±95 | 130 ±37 |
| PSME_00013643-RA | pleiotropic drug resistance 1 | AT1G66950.1 | GO:0098519 GO:0016887 GO:0016301 GO:0015093 GO:0005525 GO:0005524 GO:0003924 GO:0003724 GO:0003723 | GO:0015684 GO:0007264 GO:0006576 GO:0002949 | GO:0016021 GO:0016020 | NE | 249,4 | $1e^{-21}$ | 5 ±1 | 1634 ±681 |
| PSME_00014534-RA | GDSL esterase/lipase At4g01130 | AT4G01130.1 | GO:0003723 | | | EM | 11,7 | $1e^{-09}$ | 726 ±81 | 58 ±28 |
| PSME_00014771-RA | Os10g0458700 | AT3G01410.1 | GO:0098519 GO:0016887 GO:0016301 GO:0009378 GO:0005525 GO:0005524 GO:0003924 GO:0003724 GO:0003723 | GO:0006810 GO:0006614 GO:0006576 GO:0006310 GO:0006281 GO:0002949 | GO:0016020 | EM | 88,1 | $8e^{-14}$ | 26 ±8 | 0 ±0 |
| PSME_00014849-RA | L-ascorbate oxidase homolog | AT4G22010.1 | GO:0015078 GO:0009982 GO:0003735 GO:0003723 | GO:0015986 GO:0009451 GO:0006412 GO:0001522 | GO:0045263 GO:0005840 | EM | 49,6 | $5e^{-41}$ | 864 ±105 | 16 ±4 |
| PSME_00015395-RA | Coatomer subunit beta-1 | AT1G15520.1 | GO:0016887 GO:0009378 GO:0005524 GO:0003724 GO:0003723 | GO:0006310 GO:0006281 | | EM | 2,6 | $1e^{-12}$ | 39031 ±2358 | 15004 ±1623 |
| PSME_00015801-RA | pectinesterase isoform X1 | AT5G19730.1 | GO:0098519 GO:0043531 GO:0016887 GO:0016787 GO:0016301 GO:0009378 GO:0008134 GO:0005524 GO:0003724 GO:0003723 GO:0003678 GO:0003677 | GO:0019079 GO:0006355 GO:0006310 GO:0006281 GO:0002949 | | EM | 5,5 | $4e^{-08}$ | 18 ±3 | 3 ±1 |
| PSME_00016380-RA | cell division cycle 48 homolog | AT5G03340.1 | GO:0098519 GO:0043531 GO:0016887 GO:0016301 GO:0009378 GO:0008134 GO:0005524 GO:0003724 GO:0003723 GO:0003678 | GO:0019079 GO:0006355 GO:0006310 GO:0006281 GO:0002949 | | NE | 12,3 | $3e^{-07}$ | 1 ±0 | 11 ±3 |
| PSME_00016694-RA | polygalacturonase non-catalytic subunit JP650 | AT1G70370.1 | GO:0043531 GO:0016887 GO:0016301 GO:0009378 GO:0008134 GO:0005524 GO:0004127 GO:0003724 GO:0003723 GO:0003678 | GO:0019079 GO:0006576 GO:0006355 GO:0006310 GO:0006281 GO:0006139 GO:0002949 | | EM | 3,9 | $5e^{-08}$ | 2067 ±296 | 522 ±85 |
| PSME_00017230-RA | calmodulin binding | AT3G58480.1 | GO:0016887 GO:0008134 GO:0005525 GO:0005524 GO:0003924 GO:0003724 GO:0003723 GO:0003678 | GO:0006810 GO:0006614 GO:0006355 | | EM | 3,5 | $4e^{-08}$ | 1258 ±259 | 356 ±38 |
| PSME_00017263-RA | asparagine synthetase | AT3G47340.1 | GO:0098519 GO:0043531 GO:0016887 GO:0016301 GO:0009378 GO:0008134 GO:0005524 GO:0003724 GO:0003723 GO:0003678 | GO:0019079 GO:0006810 GO:0006355 GO:0006310 GO:0006281 GO:0002949 GO:0000723 | | EM | 5,8 | $2e^{-12}$ | 159 ±29 | 27 ±4 |
| PSME_00017644-RA | LRR receptor serine/threonine- kinase ERL1 isoform X2 | AT5G62230.1 | GO:0033897 GO:0003723 | | | EM | 3,3 | $8e^{-06}$ | 205 ±23 | 61 ±13 |
| PSME_00017672-RA | high mobility group B 15 isoform X1 | AT1G04880.1 | GO:0003723 | | | EM | 13,5 | $7e^{-08}$ | 2843 ±77 | 191 ±59 |
| PSME_00018597-RA | DEAD-box ATP-dependent RNA helicase 38 | AT3G53110.1 | GO:0098519 GO:0031683 GO:0019001 GO:0016887 GO:0016301 GO:0005525 GO:0005524 GO:0004871 GO:0003924 GO:0003724 GO:0003723 GO:0003678 | GO:0008152 GO:0007186 GO:0006810 GO:0006614 GO:0006281 GO:0000723 | | EM | 2,2 | $2e^{-13}$ | 108 ±8 | 50 ±4 |
| PSME_00019609-RA | von willebrand factor type A domain | | GO:0098519 GO:0016887 GO:0016301 GO:0009378 GO:0005525 GO:0005524 GO:0003924 GO:0003724 GO:0003723 | GO:0007264 GO:0006777 GO:0006576 GO:0006310 GO:0006281 GO:0002949 | GO:0016020 | EM | 4 | $3e^{-05}$ | 208 ±17 | 51 ±11 |
| PSME_00020362-RA | ubiquitin fusion degradation 1 homolog isoform X2 | AT2G21270.3 | GO:0003723 | GO:0006511 GO:0006353 | | NE | 61 | $2e^{-09}$ | 0 ±0 | 12 ±3 |

| | | | | | | | | | | |
|---|---|---|---|---|---|---|---|---|---|---|
| PSME_00020723-RA | O-acyltransferase WSD1 | AT5G53390.1 | GO:0046872 GO:0004535 GO:0003723 GO:0003676 | GO:0006402 | GO:0005737 GO:0005634 | EM | 131,5 | $1e^{-21}$ | 293 ±81 | 2 ±1 |
| PSME_00021307-RA | TF PosF21 | AT2G42380.2 | GO:0098519 GO:0016887 GO:0016301 GO:0005525 GO:0005524 GO:0003724 GO:0003723 GO:0003678 | GO:0008152 GO:0006810 GO:0006614 GO:0006281 GO:0000723 | | EM | 2,1 | $7e^{-66}$ | 4571 ±155 | 2129 ±57 |
| PSME_00021865-RA | late embryogenesis abundant B19.1A | AT2G40170.1 | GO:0003723 | | | EM | 853,2 | $2e^{-58}$ | 372 ±57 | 0 ±0 |
| PSME_00023263-RA | multidrug resistance | AT3G28345.1 | GO:0042626 GO:0031683 GO:0019001 GO:0016887 GO:0016301 GO:0005525 GO:0005524 GO:0004871 GO:0003924 GO:0003724 GO:0003723 | GO:0055085 GO:0008152 GO:0007186 GO:0006810 GO:0006777 | GO:0016021 | NE | 2,1 | $1e^{-23}$ | 3850 ±161 | 8139 ±470 |
| PSME_00024031-RA | UF642 l-GalL-responsive 1 | AT5G25460.1 | GO:0098519 GO:0016887 GO:0016301 GO:0009378 GO:0005525 GO:0005524 GO:0003924 GO:0003724 GO:0003723 | GO:0007264 GO:0006614 GO:0006576 GO:0006310 GO:0006281 | GO:0016020 | EM | 9,5 | $4e^{-56}$ | 25047 ±2059 | 2625 ±283 |
| PSME_00024538-RA | cytochrome P450 78A7 | AT5G09970.1 | GO:0003723 GO:0003676 | | | EM | 50,2 | $1e^{-68}$ | 159 ±13 | 3 ±1 |
| PSME_00024700-RA | ribonuclease 2 | AT2G39780.1 | GO:0033897 GO:0003723 | | | NE | 3 | $2e^{-25}$ | 623 ±65 | 1880 ±97 |
| PSME_00024720-RA | 2-methylene-furan-3-one reductase isoform X2 | AT1G23740.1 | GO:0016887 GO:0009378 GO:0005524 GO:0003724 GO:0003723 | GO:0006367 GO:0006310 GO:0006281 | GO:0005672 | EM | 45,4 | $7e^{-27}$ | 117 ±16 | 2 ±1 |
| PSME_00024826-RA | REF/SRPP At3g05500 | AT3G05500.1 | GO:0005524 GO:0004386 GO:0003723 GO:0003676 | | GO:0070461 GO:0005634 | EM | 4,6 | $3e^{-16}$ | 103 ±12 | 22 ±3 |
| PSME_00025196-RA | pentatricopeptide repeat-containing At3g03580 | AT3G03580.1 | GO:0043531 GO:0016887 GO:0009378 GO:0008134 GO:0005524 GO:0003724 GO:0003723 GO:0003678 | GO:0019079 GO:0006355 GO:0006310 GO:0006281 GO:0002949 | | EM | 2,2 | $4e^{-11}$ | 293 ±19 | 131 ±12 |
| PSME_00025295-RA | glycosyltransferase At5g03795 | AT5G03795.1 | GO:0003723 GO:0003690 | GO:0006355 | GO:0019031 GO:0005739 | EM | 10,1 | $5e^{-06}$ | 1260 ±268 | 114 ±34 |
| PSME_00025764-RA | pentatricopeptide repeat-containing At1g73400 mitochondrial | AT1G73400.1 | GO:0046983 GO:0003735 GO:0003723 | GO:0006412 | GO:0005840 GO:0005622 | EM | 2,5 | $2e^{-16}$ | 214 ±16 | 87 ±6 |
| PSME_00025833-RA | xyloglucan endotransglucosylase/hydrolase 7 | AT5G65730.1 | GO:0005525 GO:0005524 GO:0005515 GO:0003723 | GO:0051276 GO:0007264 GO:0006406 | GO:0005737 GO:0005694 GO:0005634 | EM | 190,2 | $2e^{-24}$ | 380 ±55 | 2 ±1 |
| PSME_00026159-RA | : palmitoyl-monogalactosyldiacylglycerol delta-7 desaturase chloroplastic | AT3G15850.1 | GO:0030246 GO:0016773 GO:0005524 GO:0004560 GO:0003723 | GO:0006396 GO:0005975 GO:0002682 | | EM | 2,8 | $1e^{-07}$ | 41 ±4 | 15 ±2 |
| PSME_00026932-RA | phosphoenolpyruvate carboxylase 4 | AT1G68750.1 | GO:0008964 GO:0003723 | GO:0015977 GO:0006099 | GO:0005829 | EM | 3,1 | $4e^{-10}$ | 16 ±1 | 5 ±1 |
| PSME_00027099-RA | CBL-interacting serine/threonine- kinase 23 isoform X3 | AT1G30270.1 | GO:0003723 GO:0003676 | | | EM | 3 | $4e^{-05}$ | 83 ±11 | 27 ±5 |
| PSME_00027997-RA | LURP-one-related 8 | AT3G15810.1 | GO:0016887 GO:0005525 GO:0005524 GO:0003924 GO:0003724 GO:0003723 | GO:0006614 GO:0006576 | | EM | 22,3 | $2e^{-06}$ | 15 ±6 | 0 ±0 |
| PSME_00028192-RA | GATA TF 22 | AT5G49300.1 | GO:0033897 GO:0003723 | | | EM | 60,9 | $2e^{-16}$ | 1100 ±187 | 15 ±8 |
| PSME_00028416-RA | delta(24)-sterol reductase | AT3G19820.1 | GO:0008982 GO:0008536 GO:0005524 GO:0005515 GO:0004674 GO:0003968 GO:0003723 | GO:0019079 GO:0016192 GO:0009401 GO:0006886 | GO:0030117 GO:0016021 | EM | 3,3 | $4e^{-10}$ | 1123 ±182 | 337 ±37 |
| PSME_00028893-RA | 60S ribosomal L8 | AT2G18020.1 | GO:0003735 GO:0003723 | GO:0002181 | GO:0022625 | NE | 100,1 | $9e^{-32}$ | 1 ±0 | 95 ±19 |
| PSME_00030323-RA | notum homolog isoform X1 | AT4G19420.1 | GO:0016787 GO:0005525 GO:0003746 GO:0003723 | GO:0001514 | GO:0005737 | NE | 9,8 | $1e^{-39}$ | 305 ±43 | 3010 ±275 |
| PSME_00030404-RA | metacaspase-9 isoform X2 | AT5G04200.1 | GO:0003723 | | | EM | 6,2 | $4e^{-12}$ | 534 ±44 | 84 ±14 |

| | | | | | | | | | | |
|---|---|---|---|---|---|---|---|---|---|---|
| PSME_00030551-RA | 5-methyltetrahydropteroyltriglutamate--homocysteine methyltransferase 1 isoform X4 | AT5G17920.1 | GO:0004809 GO:0003723 | GO:0008033 | | EM | 3,8 | $1e^{-09}$ | 681 ±59 | 177 ±30 |
| PSME_00030804-RA | DEAD box RNA helicase isoform 2 | AT5G14610.1 | GO:0008173 GO:0003723 | GO:0006396 | | EM | 6,5 | $2e^{-05}$ | 14 ±3 | 2 ±1 |
| PSME_00031165-RA | indole-3-acetate O-methyltransferase 1 | AT5G55250.1 | GO:0098519 GO:0016887 GO:0016301 GO:0005525 GO:0005524 GO:0003924 GO:0003724 GO:0003723 | GO:0006810 GO:0006576 GO:0002949 | GO:0016020 | EM | 246,2 | $5e^{-43}$ | 242 ±47 | 1 ±0 |
| PSME_00031180-RA | ABC transporter E member 2 | AT4G19210.1 | GO:0098519 GO:0043531 GO:0031683 GO:0019001 GO:0016887 GO:0005525 GO:0005524 GO:0004871 GO:0003924 GO:0003724 GO:0003723 GO:0003677 | GO:0030261 GO:0007186 GO:0007059 GO:0006614 GO:0002949 | GO:0009295 | NE | 2,2 | $5e^{-09}$ | 340 ±36 | 742 ±57 |
| PSME_00032223-RA | serine/arginine-rich SC35 splicing factor SCL28 | AT3G13570.1 | GO:0003723 GO:0003677 GO:0000166 | GO:0007283 | GO:0016607 GO:0000786 | EM | 2,7 | $8e^{-14}$ | 1333 ±107 | 490 ±54 |
| PSME_00032247-RA | Os09g0400200 | AT4G39330.1 | GO:0004185 GO:0003723 | | GO:0005773 | EM | 22,6 | $1e^{-08}$ | 581 ±164 | 22 ±8 |
| PSME_00032964-RA | protease Do 7 | AT3G03380.1 | GO:0070009 GO:0008239 GO:0005524 GO:0005515 GO:0004252 GO:0003724 GO:0003723 | GO:0006508 | | NE | 3,8 | $3e^{-10}$ | 38 ±3 | 145 ±20 |
| PSME_00032965-RA | protease Do 7 isoform X2 | AT3G03380.1 | GO:0070009 GO:0008239 GO:0005524 GO:0005515 GO:0004252 GO:0003724 GO:0003723 | GO:0019082 GO:0016032 GO:0006508 | | NE | 3,8 | $1e^{-28}$ | 2909 ±185 | 10951 ±894 |
| PSME_00032966-RA | protease Do 7 isoform X1 | AT3G03380.1 | GO:0070009 GO:0008239 GO:0008236 GO:0005524 GO:0005515 GO:0004252 GO:0003724 GO:0003723 | GO:0019087 GO:0019082 GO:0016032 GO:0006508 | | NE | 2,2 | $9e^{-10}$ | 366 ±30 | 808 ±62 |
| PSME_00032968-RA | Cyclic nucleotide gated channel 5 isoform 1 | AT1G15990.1 | GO:0033897 GO:0005515 GO:0003723 | | | NE | 2,1 | $1e^{-28}$ | 964 ±59 | 2006 ±56 |
| PSME_00033756-RA | G2/mitotic-specific cyclin-2 isoform X1 | AT1G76310.1 | GO:0003723 GO:0003676 | | | EM | 2,3 | $2e^{-13}$ | 83 ±6 | 36 ±3 |
| PSME_00033759-RA | thioredoxin reductase 1 | AT2G17420.1 | GO:0003723 GO:0003676 | | | EM | 2,6 | $6e^{-08}$ | 338 ±35 | 129 ±21 |
| PSME_00034056-RA | omega-hydroxypalmitate O-feruloyl transferase isoform X2 | AT1G65450.1 | GO:0016887 GO:0016787 GO:0016301 GO:0008134 GO:0005525 GO:0005524 GO:0005515 GO:0003924 GO:0003724 GO:0003723 GO:0003677 | GO:0019538 GO:0007264 GO:0006810 GO:0006355 GO:0002949 | | EM | 690,1 | $4e^{-67}$ | 3320 ±520 | 4 ±2 |
| PSME_00034347-RA | aminotransferase TAT2 | AT5G53970.1 | GO:0003735 GO:0003723 | GO:0006412 | GO:0005840 GO:0005622 | EM | 6 | $1e^{-08}$ | 284 ±19 | 46 ±12 |
| PSME_00035260-RA | TCM_016710 | AT4G19390.1 | GO:0016887 GO:0005525 GO:0005524 GO:0003924 GO:0003724 GO:0003723 | | GO:0016020 | EM | 4,2 | $2e^{-08}$ | 492 ±35 | 117 ±20 |
| PSME_00035514-RA | GTP-binding nuclear Ran1A | AT5G55190.1 | GO:0031683 GO:0019001 GO:0016887 GO:0005525 GO:0005524 GO:0004871 GO:0003924 GO:0003724 GO:0003723 | GO:0007264 GO:0007186 | | NE | 2013 | $1e^{-33}$ | 0 ±0 | 1341 ±457 |
| PSME_00036270-RA | myb-related 306 | AT5G57620.1 | GO:0098519 GO:0016887 GO:0016301 GO:0009378 GO:0005525 GO:0005524 GO:0003924 GO:0003724 GO:0003723 | GO:0007264 GO:0006777 GO:0006614 GO:0006310 GO:0006281 | GO:0016020 | EM | 9,3 | $8e^{-09}$ | 181 ±26 | 18 ±4 |
| PSME_00036372-RA | Senescence/dehydration-associated | | GO:0070009 GO:0008239 GO:0005524 GO:0005515 GO:0004252 GO:0003724 GO:0003723 GO:0003677 | GO:0016032 GO:0006508 | GO:0016021 | EM | 7,4 | $6e^{-05}$ | 46 ±8 | 6 ±2 |
| PSME_00036602-RA | SET domain 35 | AT1G26760.1 | GO:0098519 GO:0016887 GO:0016301 GO:0009378 GO:0005525 GO:0005524 GO:0003924 GO:0003724 GO:0003723 | GO:0007264 GO:0006777 GO:0006576 GO:0006310 GO:0006281 GO:0002949 | GO:0016020 | EM | 14,7 | $6e^{-12}$ | 54 ±9 | 3 ±1 |

| | | | | | | | | | | |
|---|---|---|---|---|---|---|---|---|---|---|
| PSME_00036962-RA | DEAD-box ATP-dependent RNA helicase 38 | AT3G53110.1 | GO:0016779 GO:0003723 | GO:0006396 | | EM | 2 | $5e^{-14}$ | 425 ±20 | 208 ±15 |
| PSME_00037269-RA | germin 2-1 | AT4G14630.1 | GO:0004518 GO:0003723 GO:0003677 | | | EM | 13,1 | $9e^{-09}$ | 144 ±42 | 10 ±2 |
| PSME_00038561-RA | phosphoglycerate kinase | AT1G79550.1 | GO:0003735 GO:0003723 | GO:0006412 | GO:0005840 | EM | 5,8 | $1e^{-04}$ | 255 ±30 | 42 ±27 |
| PSME_00038590-RA | Pre-mRNA cleavage complex 2 Pcf11 isoform 3 | AT3G22550.1 | GO:0004540 GO:0003723 | GO:0008033 GO:0006379 GO:0006364 | GO:0030677 GO:0000172 | EM | 14,7 | $7e^{-10}$ | 54 ±14 | 3 ±1 |
| PSME_00039496-RA | serine/threonine- phosphatase 6 regulatory ankyrin repeat subunit C | AT3G28880.1 | GO:0045182 GO:0005525 GO:0005506 GO:0005089 GO:0003746 GO:0003723 | GO:0006417 GO:0001514 | GO:0005737 | EM | 64,3 | $1e^{-07}$ | 30 ±8 | 0 ±0 |
| PSME_00040631-RA | RING-H2 finger ATL74 | AT3G10910.1 | GO:0003723 | | | EM | 4,2 | $1e^{-07}$ | 130 ±14 | 30 ±6 |
| PSME_00041065-RA | SRG1 isoform X1 | AT3G21420.1 | GO:0043531 GO:0016887 GO:0016301 GO:0009378 GO:0005524 GO:0003724 GO:0003723 GO:0003678 | GO:0019079 GO:0006310 GO:0006281 GO:0002949 | | EM | 21,4 | $2e^{-14}$ | 859 ±94 | 37 ±10 |
| PSME_00041471-RA | dnaJ homolog sub B member 6 isoform X3 | AT1G56300.1 | GO:0003723 | | | EM | 3 | $2e^{-11}$ | 833 ±37 | 277 ±38 |
| PSME_00041502-RA | pectate lyase 8 | AT4G13710.1 | GO:0045182 GO:0005525 GO:0003746 GO:0003723 | GO:0016192 GO:0006886 GO:0006417 GO:0001514 GO:0000372 | GO:0030529 GO:0005737 | EM | 6,1 | $6e^{-15}$ | 142 ±29 | 23 ±2 |
| PSME_00041701-RA | LURP-one-related 8 isoform X2 | AT2G38640.1 | GO:0005525 GO:0003724 GO:0003723 | GO:0007264 | | EM | 117,4 | $6e^{-41}$ | 148 ±21 | 1 ±1 |
| PSME_00041732-RA | acyl-coenzyme A thioesterase 13 | AT2G29590.1 | GO:0043531 GO:0016887 GO:0016787 GO:0009378 GO:0008134 GO:0005524 GO:0003724 GO:0003723 GO:0003678 GO:0003677 | GO:0019079 GO:0006355 GO:0006310 GO:0006281 GO:0002949 | | EM | 4,4 | $1e^{-05}$ | 36 ±8 | 8 ±2 |
| PSME_00041971-RA | purple acid phosphatase 23 | AT3G07130.1 | GO:0043565 GO:0016779 GO:0003723 GO:0003700 | GO:0006396 GO:0006355 GO:0006351 | | EM | 3 | $1e^{-04}$ | 1796 ±368 | 584 ±120 |
| PSME_00042006-RA | receptor kinase At5g47070 | AT5G03320.1 | GO:0003723 GO:0003676 | | | EM | 9,9 | $2e^{-08}$ | 31 ±5 | 3 ±1 |
| PSME_00043286-RA | intracellular ribonuclease LX | AT2G02990.1 | GO:0033897 GO:0003723 | | | NE | 7 | $6e^{-08}$ | 4 ±1 | 25 ±4 |
| PSME_00043623-RA | QWRF motif-containing 2 | AT4G30710.1 | GO:0016887 GO:0009378 GO:0008134 GO:0005524 GO:0003724 GO:0003723 GO:0003678 | GO:0006355 GO:0006310 GO:0006281 GO:0002949 | | EM | 17,1 | $1e^{-07}$ | 14 ±4 | 1 ±0 |
| PSME_00044624-RA | glutelin type-A 1 | AT2G28680.1 | GO:0016887 GO:0009378 GO:0008134 GO:0005524 GO:0003724 GO:0003723 GO:0003678 | GO:0006355 GO:0006310 GO:0006281 GO:0002949 | | EM | 15,1 | $2e^{-65}$ | 28285 ±4110 | 1855 ±134 |
| PSME_00046060-RA | knotted 1-binding | AT5G03050.1 | GO:0003723 | GO:0070206 | | NE | 6,2 | $7e^{-10}$ | 11 ±3 | 68 ±6 |
| PSME_00046806-RA | mitochondrial chaperone BCS1-B | AT3G28580.1 | GO:0016887 GO:0009378 GO:0005524 GO:0003724 GO:0003723 | GO:0006310 GO:0006281 | | NE | 48 | $3e^{-32}$ | 53 ±8 | 2727 ±473 |
| PSME_00048125-RA | isoform 1 | AT3G51290.1 | GO:0003723 | GO:0006777 | | EM | 17,4 | $3e^{-17}$ | 4883 ±210 | 265 ±69 |
| PSME_00048243-RA | RNA-binding (RRM/RBD/RNP motifs) | AT1G14340.1 | GO:0003723 GO:0003676 | | | NE | 4,5 | $1e^{-33}$ | 1083 ±114 | 4931 ±348 |
| PSME_00048431-RA | Os03g0151800 | AT5G03340.1 | GO:0043531 GO:0016887 GO:0009378 GO:0005524 GO:0003724 GO:0003723 GO:0003678 | GO:0019079 GO:0006310 GO:0006281 GO:0002949 | | NE | 18,4 | $4e^{-06}$ | 1 ±1 | 30 ±9 |
| PSME_00048472-RA | cytochrome P450 CYP736A12 | AT3G48280.1 | GO:0008173 GO:0003723 | GO:0006396 | | EM | 204,1 | $6e^{-46}$ | 51 ±8 | 0 ±0 |
| PSME_00048657-RA | glucan endo-13-beta-glucosidase A6 | AT3G07320.1 | GO:0003723 | | | EM | 4,3 | $3e^{-06}$ | 68 ±15 | 15 ±3 |

| | | | | | | | | | | |
|---|---|---|---|---|---|---|---|---|---|---|
| PSME_00048908-RA | pectate lyase 8 isoform X1 | AT3G07010.1 | GO:0003723 | | | EM | 1143 | $4e^{-55}$ | 323 ±82 | 0 ±0 |
| PSME_00050702-RA | Os05g0171900 | AT1G80160.1 | GO:0003723 | | | NE | 32,3 | $9e^{-15}$ | 6 ±2 | 228 ±38 |
| PSME_00050753-RA | SOMBRERO | AT1G79580.1 | GO:0003723 | | | EM | 7,9 | $1e^{-17}$ | 13815 ±2082 | 1706 ±290 |
| PSME_00051837-RA | actin | AT5G09810.1 | GO:0098519 GO:0043531 GO:0016887 GO:0016787 GO:0016301 GO:0009378 GO:0008134 GO:0005524 GO:0003724 GO:0003723 GO:0003678 GO:0003677 | GO:0019079 GO:0006355 GO:0006310 GO:0006281 GO:0002949 | | EM | 3,5 | $5e^{-33}$ | 490 ±31 | 138 ±10 |
| PSME_00051945-RA | 5-methyltetrahydropteroyltriglutamate--homocysteine methyltransferase | AT3G03780.1 | GO:0008897 GO:0003723 GO:0003676 GO:0000287 | | | EM | 5,7 | $2e^{-07}$ | 16 ±2 | 3 ±1 |
| PSME_00052130-RA | transporter arsB | AT1G02260.1 | GO:0003723 | | | EM | 3,8 | $3e^{-11}$ | 75 ±6 | 20 ±3 |
| PSME_00052451-RA | long-chain-alcohol oxidase FAO2 | AT3G23410.1 | GO:0033897 GO:0003723 | | | EM | 15,8 | $1e^{-12}$ | 20 ±3 | 1 ±0 |
| PSME_00052605-RA | xyloglucan endotransglucosylase/hydrolase 9 | AT4G03210.1 | GO:0005524 GO:0004386 GO:0003723 GO:0003676 | | GO:0005634 | EM | 4,2 | $9e^{-06}$ | 389 ±76 | 90 ±20 |
| PSME_00052678-RA | mitochondrial chaperone BCS1-B | AT3G28510.1 | GO:0016887 GO:0009378 GO:0008918 GO:0005524 GO:0003724 GO:0003723 GO:0003677 | GO:0009103 GO:0006313 GO:0006310 GO:0006281 | | NE | 3,1 | $3e^{-08}$ | 1625 ±280 | 5142 ±516 |
| PSME_00052764-RA | histone H2AX | AT1G54690.1 | GO:0098519 GO:0043531 GO:0016887 GO:0016301 GO:0009378 GO:0008134 GO:0005524 GO:0003724 GO:0003723 GO:0003678 | GO:0019079 GO:0006576 GO:0006355 GO:0006310 GO:0006281 GO:0002949 | | EM | 2,1 | $1e^{-40}$ | 55932 ±2086 | 26005 ±1011 |
| PSME_00053348-RA | endoribonuclease Dicer homolog 1 isoform X1 | AT3G20420.1 | GO:0004525 GO:0003723 | GO:0006396 | | NE | 4,2 | $5e^{-11}$ | 212 ±16 | 890 ±128 |
| PSME_00054009-RA | uninformative | | GO:0003723 | | | EM | 20,9 | $1e^{-18}$ | 12 ±2 | 0 ±0 |
| PSME_00054332-RA | Cytochrome P450 | AT2G45550.1 | GO:0003723 | | GO:0005743 | EM | 31,1 | $4e^{-12}$ | 10 ±3 | 0 ±0 |
| PSME_00055130-RA | glucan endo-13-beta-glucosidase 5 | AT5G58480.1 | GO:0004521 GO:0003723 | | | EM | 4,2 | $3e^{-17}$ | 143 ±9 | 33 ±5 |
| PSME_00056488-RA | subtilisin protease | AT3G14240.1 | GO:0008173 GO:0008168 GO:0003723 | GO:0006396 | GO:0016021 | EM | 216,7 | $5e^{-24}$ | 62 ±16 | 0 ±0 |
| PSME_00056929-RA | alpha-14-glucan- synthase | AT3G02230.1 | GO:0098519 GO:0043531 GO:0016887 GO:0016787 GO:0016740 GO:0016301 GO:0009378 GO:0008134 GO:0005524 GO:0003724 GO:0003723 GO:0003678 GO:0003677 | GO:0019079 GO:0006576 GO:0006355 GO:0006310 GO:0006281 GO:0002949 | | EM | 2,8 | $6e^{-21}$ | 2010 ±104 | 707 ±57 |
| PSME_00056941-RA | mitochondrial chaperone BCS1 | AT2G46620.1 | GO:0016887 GO:0009378 GO:0005524 GO:0003724 GO:0003723 | GO:0006310 GO:0006281 | | NE | 2,1 | $3e^{-17}$ | 310 ±15 | 660 ±40 |
| PSME_00057055-RA | : H/ACA ribonucleo complex subunit 4 | AT3G57150.1 | GO:0009982 GO:0003723 | GO:0006396 GO:0001522 | | EM | 2,5 | $6e^{-31}$ | 22349 ±1236 | 8816 ±486 |
