# Supplementary material for: Cytological, Biochemical and Molecular Events of the Embryogenic State in Douglas-fir (Pseudotsuga menziesii [Mirb.])
Source: Front Plant Sci. 2019 Feb 28;10:118. doi: 10.3389/fpls.2019.00118 (PMC6403139; doi:10.3389/fpls.2019.00118)
Supplement: Supplementary file 14 [file Table_4.pdf]

Supplementary Table S4. Characteristics of significantly differentially expressed proteins (ID Douglas-fir) in isogenic embryonal mass (EM) and non-embryogenic callus (NEC) of three genotypes (SD4-8, TD15-1 and TD17-1) of Douglas-fir after two weeks of proliferation. Functional annotation (Function), and GO ontologies (biological process) were obtained from Blast2GO and PineRefSeq. *Arabidopsis thaliana* orthologies (At_orthologs and At_protein) were obtained from Pathway Studio. FDR-adjusted p-value for differential expression were obtained from the limma R package for each genotype before mean calculations. FC: fold-change. Expr: trend of expression relative to fold-change. Mean Padj: mean of the adjusted P-value obtained for each genotype.

ID_Douglas-fir	Function	At_ortholog	At_protein	Biological process	FC	Expr.	Mean Padj.
PSME_00003908-RA	1,2-dihydroxy-3-keto-5-methylthiopentene dioxygenase 2	AT4G14710		GO:0006555 GO:0055114	0,3	NEC	$9,38\ 10^{-04}$
PSME_00017765-RA	1,4-dihydroxy-2-naphthoyl-CoA synthase, peroxisomal isoform X1	AT1G60550	ECHID		0,4	NEC	$4,31\ 10^{-03}$
PSME_00029162-RA	12-oxophytodienoate reductase 2-like isoform X1	AT1G76690	OPR2	GO:0055114 GO:0009611	3,2	EM	$1,54\ 10^{-04}$
PSME_00046623-RA	12-oxophytodienoate reductase 7	AT2G06050	OPR3	GO:0009620 GO:0055114 GO:0009611 GO:0009695	0,3	NEC	$6,16\ 10^{-06}$
PSME_00005541-RA	14-3-3-like protein D	AT1G26480	GRF12		0,8	NEC	$2,16\ 10^{-02}$
PSME_00037344-RA	17.6 kDa class I heat shock protein-like	AT1G53540	AT1G53540.1	GO:0009408	0,1	NEC	$1,68\ 10^{-04}$
PSME_00023023-RA	2,3-bisphosphoglycerate-independent phosphoglycerate mutase	AT1G09780	iPGAM1	GO:0046686 GO:0006096 GO:0009409 GO:0009555 GO:0006007 GO:0010118	1,3	EM	$1,62\ 10^{-02}$
PSME_00010871-RA	2,3-bisphosphoglycerate-independent phosphoglycerate mutase, putative	AT3G30841		GO:0008152	2,6	EM	$3,15\ 10^{-03}$
PSME_00051006-RA	24-methylene lophenol C-24 methyltransferase	AT1G20330	SMT2	GO:0010051 GO:0032259 GO:0016126	2,0	EM	$7,08\ 10^{-03}$
PSME_00019628-RA	26S proteasome non-ATPase regulatory subunit 5	AT3G15180	AT3G15180.2		1,4	EM	$1,38\ 10^{-02}$
PSME_00042587-RA	2-alkenal reductase (NADP(+)-dependent)-like	AT5G16970	AER	GO:0006979 GO:0046686 GO:0055114	0,1	NEC	$2,50\ 10^{-05}$
PSME_00023357-RA	2-hydroxyacyl-CoA lyase	AT5G17380	AT5G17380.1		0,5	NEC	$3,74\ 10^{-03}$
PSME_00024224-RA	2-methylene-furan-3-one reductase-like	AT1G23740	AOR	GO:0055114 GO:0009409	0,2	NEC	$2,41\ 10^{-05}$
PSME_00028204-RA	3-demethylubiquinone-9 3-methyltransferase domain protein	AT5G48480	AT5G48480.1		3,1	EM	$1,66\ 10^{-05}$
PSME_00005757-RA	3-ketoacyl-CoA reductase 1 isoform 1	AT1G67730	KCR1	GO:0042335 GO:0055114 GO:0009790	1,7	EM	$2,06\ 10^{-03}$
PSME_00003827-RA	3-ketoacyl-CoA thiolase 2, peroxisomal	AT2G33150	PKT3	GO:0006635 GO:0009695 GO:0009611	1,4	EM	$1,30\ 10^{-02}$
PSME_00053512-RA	3-oxo-Delta(4,5)-steroid 5-beta-reductase	AT4G24220	VEP1	GO:0010051 GO:0009611 GO:0055114	0,4	NEC	$6,78\ 10^{-03}$
PSME_00020743-RA	3-phosphoshikimate 1-carboxyvinyltransferase 2	AT2G45300		GO:0009073	0,3	NEC	$1,71\ 10^{-05}$
PSME_00018601-RA	40S ribosomal protein S25-2-like	AT2G21580	AT2G21580.1	GO:0006412	1,6	EM	$5,97\ 10^{-03}$
PSME_00013554-RA	4-alpha-glucanotransferase DPE2 isoform X2	AT2G40840	DPE2		2,6	EM	$3,65\ 10^{-07}$
PSME_00006219-RA	4-coumarate--CoA ligase-like 7	AT4G05160	AT4G05160.1	GO:0009695	3,8	EM	$6,69\ 10^{-04}$
PSME_00034041-RA	5-methyltetrahydropteroyltriglutamate--homocysteine methyltransferase	AT5G17920	ATMS1	GO:0009651 GO:0032259 GO:0046686 GO:0009086	2,8	EM	$1,39\ 10^{-03}$
PSME_00053040-RA	60S ribosomal L12-like protein	AT5G60670	AT5G60670.1	GO:0006412 GO:0000027 GO:0042254	1,3	EM	$1,12\ 10^{-02}$
PSME_00006747-RA	60S ribosomal protein L15-1	AT4G16720	AT4G16720.1	GO:0006412	1,5	EM	$7,69\ 10^{-03}$
PSME_00002291-RA	60S ribosomal protein L27a-3	AT1G70600		GO:0006412	1,5	EM	$6,35\ 10^{-03}$
PSME_00052545-RA	60S ribosomal protein L35	AT5G02610	AT5G02610.2	GO:0006412 GO:0042254	1,6	EM	$8,66\ 10^{-03}$
PSME_00003145-RA	60S ribosomal protein L4	AT3G09630	SAC56	GO:0002181 GO:0006412	1,2	EM	$1,89\ 10^{-02}$
PSME_00005856-RA	60S ribosomal protein L8	AT4G36130		GO:0006412	2,0	EM	$5,67\ 10^{-05}$
PSME_00013038-RA	66 kDa stress protein isoform X1	AT3G18060	AT3G18060.1		2,5	EM	$1,70\ 10^{-04}$

PSME_00016643-RA	8-hydroxygeraniol dehydrogenase-like	AT3G19450	ATCAD4	GO:0009809 GO:0055114 GO:0080167	0,6	NEC	5,58 10^{-04}
PSME_00015981-RA	ABC transporter F family member 1-like	AT5G60790	ABCF1	GO:0006810	1,6	EM	6,77 10^{-04}
PSME_00045612-RA	ABC transporter F family member 4-like	AT3G54540	ABCF4	GO:0006810	1,9	EM	2,35 10^{-05}
PSME_00019197-RA	acid phosphatase 1-like	AT4G29260	AT4G29260.1		2,3	EM	5,92 10^{-04}
PSME_00007485-RA	actin	AT5G09810	ACT7	GO:0051301 GO:0009845 GO:0009611 GO:0048364 GO:0007010 GO:0009416 GO:0009733	5,4	EM	2,07 10^{-05}
PSME_00008062-RA	actin-depolymerizing factor 7-like	AT4G00680	ADF8		1,6	EM	1,96 10^{-04}
PSME_00011991-RA	actin-like	AT3G12110	ACT11	GO:0030036	6,0	EM	1,21 10^{-07}
PSME_00044995-RA	activator of 90 kDa heat shock protein ATPase homolog	AT3G12050	AT3G12050.1	GO:0006950	1,8	EM	3,41 10^{-03}
PSME_00009588-RA	acyl-CoA-binding domain-containing protein 4-like isoform X2	AT3G05420	ACBP4	GO:0009723 GO:0006869 GO:0009416 GO:0009753	1,9	EM	5,14 10^{-04}
PSME_00004950-RA	Adenosine kinase 2	AT5G03300	ADK2	GO:0016032 GO:0006166 GO:0016310	1,8	EM	1,16 10^{-07}
PSME_00001927-RA	alanine aminotransferase 2	AT1G72330		GO:0009058	13,8	EM	3,88 10^{-06}
PSME_00001072-RA	alcohol dehydrogenase-like	AT1G77120	ADH1	GO:0009414 GO:0046686 GO:0009737 GO:0009651 GO:0009744 GO:0006970 GO:0009409 GO:0055114 GO:0042542	0,3	NEC	1,23 10^{-06}
PSME_00035677-RA	aldehyde dehydrogenase family 2 member C4 isoform X2	AT3G48000	ALDH2B4	GO:0055114 GO:0046686	0,2	NEC	1,63 10^{-02}
PSME_00052443-RA	allene oxide synthase	AT5G42650	AOS	GO:0016125 GO:0009611 GO:0009695 GO:0050832 GO:0009753 GO:0006952 GO:0031407 GO:0009620 GO:0055114	0,4	NEC	5,40 10^{-03}
PSME_00008870-RA	allene oxide synthase, chloroplastic-like	AT5G42650	AOS	GO:0031407 GO:0009620 GO:0006952 GO:0055114 GO:0050832 GO:0009753 GO:0009695 GO:0016125 GO:0009611	0,1	NEC	2,37 10^{-06}
PSME_00033948-RA	alpha,alpha-trehalose-phosphate synthase	AT4G17770	TPS5	GO:0005992	0,5	NEC	1,69 10^{-03}
PSME_00016742-RA	alpha-1,4 glucan phosphorylase L isozyme, chloroplastic/amyloplastic	AT3G29320	PHS1	GO:0009414 GO:0009266	0,4	NEC	8,41 10^{-03}
PSME_00046007-RA	alpha-1,4-glucan-protein synthase	AT3G08900	RGP3	GO:0071555 GO:0030244 GO:0033356 GO:0009832	1,4	EM	4,34 10^{-03}
PSME_00009571-RA	alpha-L-arabinofuranosidase 1	AT3G10740	ASD1	GO:0045493 GO:0046373	0,3	NEC	4,42 10^{-07}
PSME_00039135-RA	alpha-soluble NSF attachment protein-like	AT3G56190	ALPHA-SNAP2	GO:0006886	1,5	EM	4,54 10^{-03}
PSME_00027843-RA	amidase 1-like isoform X1	AT3G17970	TOC64-III	GO:0015031	1,5	EM	1,52 10^{-03}
PSME_00048866-RA	aminoacylase-1 isoform X2	AT4G38220	AQI		0,6	NEC	1,23 10^{-04}
PSME_00001199-RA	anamorsin homolog	AT5G18400	DRE2	GO:0016226	0,1	NEC	3,89 10^{-03}
PSME_00021228-RA	anthocyanidin reductase	AT1G61720	BAN	GO:0055114 GO:0009813	0,2	NEC	9,88 10^{-05}
PSME_00046128-RA	anthocyanidin reductase	AT5G42800	DFR	GO:0055114	0,2	NEC	6,25 10^{-04}
PSME_00000575-RA	anthranilate synthase alpha subunit 2, chloroplastic isoform X2	AT2G29690	ASA2	GO:0009073 GO:0009058	1,3	EM	1,09 10^{-02}
PSME_00001853-RA	apoptosis-inducing factor homolog B-like	AT3G44190	AT3G44190.1	GO:0055114	6,2	EM	2,74 10^{-03}
PSME_00009870-RA	aspartate aminotransferase, chloroplastic	AT4G31990	ASP5	GO:0009409 GO:0009058 GO:0046686 GO:0006520	1,3	EM	8,40 10^{-03}
PSME_00052836-RA	aspartic proteinase nepenthesin-1-like	AT3G25700			9,0	EM	1,77 10^{-06}
PSME_00007040-RA	aspartic proteinase nepenthesin-2	AT3G25700			6,0	EM	3,79 10^{-05}
PSME_00035372-RA	ATP sulfurylase 2	AT1G19920	APS2		2,2	EM	6,46 10^{-03}
PSME_00020216-RA	ATPase 1, plasma membrane-type	AT1G80660	HA9	GO:0015992 GO:0006754	2,9	EM	4,13 10^{-06}
PSME_00016475-RA	ATPase, AAA domain containing protein				0,4	NEC	7,95 10^{-03}
PSME_00013241-RA	ATP-dependent 6-phosphofructokinase 3-like	AT5G61580	PFK4	GO:0006002 GO:0006096	0,7	NEC	1,22 10^{-02}
PSME_00000476-RA	ATP-dependent chaperone ClpB	AT1G74310	HSP101	GO:0009408 GO:0009644 GO:0019538 GO:0042542	0,2	NEC	2,39 10^{-06}
PSME_00000131-RA	ATP-dependent Clp protease ATP-binding subunit clpA homolog CD4B, chloroplastic	AT5G50920	CLPC1	GO:0045037 GO:0009658 GO:0009735	1,7	EM	3,15 10^{-03}
PSME_00040415-RA	ATP-dependent RNA helicase-like protein DB10	AT5G14610	AT5G14610.1		2,5	EM	8,08 10^{-04}

PSME_00047423-RA	auxin-repressed 12.5 kDa protein-like	AT1G28330	DYL1	GO:0009744	0,2	NEC	7,35 10^{-04}
PSME_00044312-RA	beta-adaptin-like protein A	AT5G11490	AT5G11490.2	GO:0016192 GO:0006886	1,9	EM	1,98 10^{-03}
PSME_00013828-RA	beta-adaptin-like protein C	AT4G23460	AT4G23460.1	GO:0006886 GO:0016192	1,2	EM	1,28 10^{-02}
PSME_00023147-RA	beta-D-xylosidase 1-like	AT5G64570	XYL4	GO:0045493 GO:0005975	0,2	NEC	1,07 10^{-02}
PSME_00041230-RA	beta-xylosidase/alpha-L-arabinofuranosidase 2	AT5G64570	XYL4	GO:0005975 GO:0045493	0,3	NEC	2,86 10^{-04}
PSME_00031787-RA	bifunctional 3-dehydroquinate dehydratase/shikimate dehydrogenase, chloroplastic isoform X1	AT3G06350	MEE32	GO:0055114 GO:0009073 GO:0009793	0,6	NEC	6,62 10^{-03}
PSME_00029904-RA	bifunctional aspartate aminotransferase and glutamate/aspartate-prephenate aminotransferase-like	AT2G22250	AAT	GO:0009094 GO:0009793	0,5	NEC	5,95 10^{-03}
PSME_00007495-RA	bifunctional epoxide hydrolase 2	AT3G51000	AT3G51000.1		0,5	NEC	4,35 10^{-03}
PSME_00012694-RA	bifunctional glutamate/proline--tRNA ligase-like isoform X2	AT3G62120	AT3G62120.1	GO:0006418 GO:0006433	1,4	EM	1,47 10^{-02}
PSME_00031912-RA	bifunctional L-3-cyanoalanine synthase/cysteine synthase 2, mitochondrial isoform X1	AT3G61440	CYSC1	GO:0006955 GO:0080147 GO:0019500 GO:0006535	0,3	NEC	1,71 10^{-03}
PSME_00035279-RA	bifunctional L-3-cyanoalanine synthase/cysteine synthase 2, mitochondrial isoform X1	AT3G61440	CYSC1	GO:0080147 GO:0006955 GO:0006535 GO:0019500	0,4	NEC	4,20 10^{-05}
PSME_00022884-RA	cellulose synthase-interactive protein 1	AT2G22125	CSI1	GO:0030244	2,2	EM	1,48 10^{-02}
PSME_00039185-RA	calcium-dependent protein kinase 3	AT5G12180	CPK17	GO:0046777 GO:0006468 GO:0009738 GO:0035556 GO:0018105	0,7	NEC	7,60 10^{-03}
PSME_00033520-RA	calcium-transporting ATPase 1, plasma membrane-type	AT4G37640	ACA2	GO:0006810 GO:0070588	1,4	EM	8,55 10^{-03}
PSME_00001231-RA	callose synthase 10	AT2G36850	GSL8	GO:0006075 GO:0071555 GO:0009555 GO:0008360	2,5	EM	7,44 10^{-04}
PSME_00010506-RA	cathepsin B-like	AT1G02305	AT1G02305.1	GO:0006508 GO:0050790 GO:0006952 GO:0051603	2,5	EM	5,27 10^{-04}
PSME_00047559-RA	cationic peroxidase 1-like	AT5G05340	PRX52	GO:0006979 GO:0042744 GO:0055114 GO:0009809	0,2	NEC	9,05 10^{-03}
PSME_00000477-RA	chaperone protein ClpB1	AT1G74310	HSP101	GO:0009408 GO:0042542 GO:0019538 GO:0009644	0,2	NEC	9,04 10^{-05}
PSME_00000058-RA	chaperonin 60 subunit beta 2, chloroplastic isoform X2	AT3G13470	Cpn60beta2	GO:0061077 GO:0006458 GO:0007005	0,6	NEC	2,26 10^{-03}
PSME_00046974-RA	chorismate mutase 1, chloroplastic	AT3G29200	CM1	GO:0055114 GO:0098869 GO:0046417 GO:0009073	0,4	NEC	9,82 10^{-04}
PSME_00032578-RA	cinnamoyl-CoA reductase 1-like	AT5G19440	AT5G19440.1		0,2	NEC	2,11 10^{-05}
PSME_00006186-RA	citrate synthase, mitochondrial	AT2G44350	ATCS	GO:0046686 GO:0006099	0,7	NEC	2,08 10^{-02}
PSME_00005056-RA	CO(2)-response secreted protease	AT1G20160	ATSBT5.2	GO:0006508	0,2	NEC	7,02 10^{-05}
PSME_00010853-RA	CO(2)-response secreted protease	AT1G20160	ATSBT5.2	GO:0006508	0,1	NEC	3,50 10^{-08}
PSME_00044462-RA	CO(2)-response secreted protease	AT1G20160	ATSBT5.2	GO:0006508	0,3	NEC	4,23 10^{-03}
PSME_00024193-RA	coatomer subunit alpha-1	AT1G62020	AT1G62020.1	GO:0006888	1,3	EM	1,80 10^{-03}
PSME_00015316-RA	Coatomer subunit beta-1	AT1G66950	ABCG39	GO:0006855	3,7	EM	2,84 10^{-03}
PSME_00015395-RA	Coatomer subunit beta-1	AT1G15520	ABCG40	GO:0009723 GO:0002229 GO:0006810 GO:0006855 GO:0009753	3,0	EM	1,35 10^{-03}
PSME_00037059-RA	coproporphyrinogen-III oxidase 1, chloroplastic	AT1G03475	LIN2	GO:0055114 GO:0009735 GO:0033014	0,6	NEC	2,98 10^{-04}
PSME_00005557-RA	cucumber peeling cupredoxin	AT2G31050			0,4	NEC	1,55 10^{-03}
PSME_00019416-RA	cyprosin	AT1G11910	APA1	GO:0009651 GO:0006629 GO:0009735 GO:0006508 GO:0030163	7,8	EM	4,19 10^{-04}
PSME_00022064-RA	cyprosin	AT1G11910	APA1	GO:0030163 GO:0006508 GO:0009735 GO:0006629 GO:0009651	1,6	EM	3,13 10^{-03}
PSME_00029276-RA	cysteine proteinase RD19a-like	AT4G39090	RD19	GO:0042742 GO:0051603 GO:0006508 GO:0009414 GO:0006970 GO:0009651	2,3	EM	2,52 10^{-05}
PSME_00021084-RA	cysteine proteinase RD21a-like	AT5G43060	RD21B	GO:0009651 GO:0051603	3,4	EM	1,89 10^{-05}
PSME_00048674-RA	cysteine-rich repeat secretory protein 15	AT2G01660	PDLP6	GO:0006810 GO:0016032	0,2	NEC	2,68 10^{-06}
PSME_00039937-RA	cytochrome P450 76C1	AT2G45560	CYP76C1	GO:0055114 GO:0044550	0,1	NEC	7,41 10^{-03}
PSME_00047109-RA	cytokinin dehydrogenase 6-like	AT1G75450	CKX5	GO:0055114 GO:0009690	0,3	NEC	4,48 10^{-03}
PSME_00022324-RA	D-3-phosphoglycerate dehydrogenase 1, chloroplastic	AT4G34200	EDA9	GO:0009793 GO:0009555 GO:0055114	0,5	NEC	2,40 10^{-05}
PSME_00017755-RA	DAG protein, chloroplastic	AT3G06790	MORF3	GO:0006397	1,8	EM	1,49 10^{-03}
PSME_00032936-RA	DDT domain-containing protein DDB_G0282237-like	AT5G08630	AT5G08630.1		2,0	EM	2,32 10^{-02}

PSME_00010814-RA	DEAD-box ATP-dependent RNA helicase 20	AT1G55150	RH20	GO:0006364 GO:0000184 GO:0010501	1,5	EM	2,26 10^{-02}
PSME_00039428-RA	DEAD-box ATP-dependent RNA helicase 53	AT3G22330		GO:0010501 GO:0009409	1,7	EM	2,03 10^{-03}
PSME_00040230-RA	delta(24)-sterol reductase	AT3G19820	DWF1	GO:0009416 GO:0009826 GO:0006694 GO:0055114 GO:0009834	2,0	EM	4,48 10^{-03}
PSME_00055611-RA	delta(24)-sterol reductase	AT3G19820	DWF1	GO:0009834 GO:0055114 GO:0006694 GO:0009826 GO:0009416	1,7	EM	2,83 10^{-04}
PSME_00000585-RA	delta-1-pyrroline-5-carboxylate synthase	AT2G39800	P5CS1	GO:0042538 GO:0055114 GO:0016310 GO:0006561 GO:0009555 GO:0009414 GO:0048364 GO:0009737 GO:0006979 GO:0009651	2,4	EM	2,48 10^{-04}
PSME_00033245-RA	dihydroflavonol-4-reductase	AT5G42800	DFR	GO:0055114	0,1	NEC	4,82 10^{-03}
PSME_00007384-RA	dihydroflavonol-4-reductase-like	AT5G42800	DFR	GO:0055114	0,3	NEC	1,44 10^{-02}
PSME_00037696-RA	Dihydrolipoamide acetyltransferase component of pyruvate dehydrogenase isoform 3	AT3G52200	LTA3	GO:0006096 GO:0008152	0,5	NEC	5,04 10^{-05}
PSME_00002566-RA	dihydrolipoyllysine-residue acetyltransferase component 4 of pyruvate dehydrogenase complex, chloroplastic	AT3G25860	LTA2	GO:0006096	2,0	EM	3,84 10^{-04}
PSME_00027089-RA	dihydrolipoyllysine-residue succinyltransferase component of 2-oxoglutarate dehydrogenase complex 2, mitochondrial-like	AT4G26910	AT4G26910.1	GO:0006099	0,6	NEC	3,96 10^{-03}
PSME_00031175-RA	dihydropyrimidinase	AT5G12200	PYD2		0,4	NEC	1,03 10^{-02}
PSME_00006584-RA	dihydropyrimidine dehydrogenase	AT3G17810	PYD1	GO:0055114	0,7	NEC	1,05 10^{-02}
PSME_00000781-RA	DNA gyrase subunit A	AT3G10690	GYRA	GO:0006355 GO:0006265 GO:0006351	1,6	EM	8,38 10^{-04}
PSME_00029161-RA	DNA repair protein recA homolog 3, mitochondrial isoform X1	AT2G19490		GO:0006281 GO:0006310	2,0	EM	5,65 10^{-04}
PSME_00048815-RA	DNA-directed RNA polymerases II, IV and V subunit 3	AT2G15430		GO:0006351	1,5	EM	2,69 10^{-02}
PSME_00020105-RA	dynamin-related protein 5A	AT5G42080	DL1	GO:0009793 GO:0010051	1,6	EM	6,08 10^{-03}
PSME_00041836-RA	electron transfer flavoprotein subunit beta, mitochondrial	AT5G43430	ETFBETA	GO:0015996 GO:0055114	0,5	NEC	2,90 10^{-03}
PSME_00019786-RA	elongation factor 1-gamma	AT1G09640	AT1G09640.1	GO:0006414	1,5	EM	1,60 10^{-04}
PSME_00008845-RA	elongation factor 1-gamma 2-like	AT1G09640	AT1G09640.1	GO:0006414	0,5	NEC	1,29 10^{-03}
PSME_00015545-RA	elongation factor Tu, mitochondrial-like isoform X1	AT4G02930	AT4G02930.1	GO:0006414 GO:0046686	1,2	EM	1,05 10^{-02}
PSME_00016232-RA	endoglucanase 23-like	AT1G23210	GH9B6	GO:0071555 GO:0030245	0,1	NEC	7,43 10^{-05}
PSME_00013360-RA	endoglucanase 3-like isoform X1	AT4G02290	GH9B13	GO:0030245 GO:0071555	0,2	NEC	4,83 10^{-04}
PSME_00034975-RA	enhanced disease susceptibility protein	AT3G48080	AT3G48080.1	GO:0006952 GO:0006629	0,4	NEC	1,45 10^{-02}
PSME_00002297-RA	enolase 1-like	AT2G36530	LOS2	GO:0009409 GO:0006355 GO:0009651 GO:0009737 GO:0006096 GO:0006351 GO:0009416 GO:0046686	1,7	EM	1,13 10^{-03}
PSME_00042482-RA	erlin-2-B	AT2G03510	AT2G03510.1		0,7	NEC	1,09 10^{-02}
PSME_00056370-RA	esterase/lipase/thioesterase family protein	AT1G52760	LysoPL2	GO:0009809 GO:0042542 GO:0006979 GO:0046686 GO:0006629	0,5	NEC	1,11 10^{-02}
PSME_00055777-RA	Eukaryotic aspartyl protease family protein isoform 1	AT3G61820	AT3G61820.1	GO:0006508	0,2	NEC	4,29 10^{-04}
PSME_00051846-RA	eukaryotic peptide chain release factor subunit 1-3	AT3G26618	ERF1-3	GO:0006415	0,7	NEC	3,53 10^{-03}
PSME_00056021-RA	eukaryotic translation elongation factor, putative	AT1G56070	LOS1	GO:0009409 GO:0009735 GO:0006414	1,6	EM	4,40 10^{-03}
PSME_00008125-RA	eukaryotic translation initiation factor 3 subunit A	AT4G11420	EIF3A	GO:0006413	1,4	EM	8,79 10^{-03}
PSME_00056689-RA	eukaryotic translation initiation factor 3 subunit G	AT3G11400	EIF3G1	GO:0006413	1,3	EM	3,92 10^{-03}
PSME_00042951-RA	eukaryotic translation initiation factor 5A-2-like	AT1G13950	ELF5A-1	GO:0045901 GO:0006452 GO:0006413 GO:0045905	1,4	EM	6,74 10^{-03}
PSME_00051817-RA	expansin-A11-like	AT1G20190	EXPA11	GO:0009664 GO:0009828 GO:0009826 GO:0009831	7,2	EM	8,06 10^{-09}
PSME_00004400-RA	expansin-like A1	AT3G45970	EXLA1	GO:0009826 GO:0009828	0,5	NEC	1,52 10^{-03}
PSME_00005463-RA	extensin	AT5G09620	AT5G09620.1		2,4	EM	1,59 10^{-02}
PSME_00022126-RA	extradiol ring-cleavage dioxygenase-like	AT4G15093	LigB	GO:0055114 GO:0006725	1,8	EM	8,34 10^{-03}
PSME_00003708-RA	far upstream element-binding protein 1-like	AT2G25970	AT2G25970.1		4,8	EM	2,62 10^{-03}

PSME_00004229-RA	ferredoxin-dependent glutamate synthase 1, chloroplastic/mitochondrial-like	AT5G04140	GLU1	GO:0009744 GO:0009853 GO:0009416 GO:0006537 GO:0055114	0,5	NEC	1,40 10^{-03}
PSME_00047906-RA	ferredoxin--NADP reductase, putative	AT4G05390	RFNR1	GO:0015979 GO:0055114	0,4	NEC	3,38 10^{-04}
PSME_00012777-RA	Ferredoxin--nitrite reductase	AT2G15620	NIR1	GO:0010167 GO:0042128 GO:0055114	0,2	NEC	1,33 10^{-02}
PSME_00045450-RA	Ferredoxin-related	AT1G02180	AT1G02180.1		4,0	EM	2,05 10^{-04}
PSME_00038196-RA	fibrous sheath CABYR-binding protein-like isoform X2				0,1	NEC	1,50 10^{-04}
PSME_00055512-RA	fibrous sheath CABYR-binding protein-like isoform X2				0,0	NEC	1,40 10^{-07}
PSME_00017291-RA	formate dehydrogenase, mitochondrial	AT5G14780	FDH	GO:0009611 GO:0046686 GO:0055114	0,4	NEC	2,23 10^{-04}
PSME_00055424-RA	fruit protein pKIWI501-like				0,2	NEC	8,42 10^{-04}
PSME_00020981-RA	fumarate hydratase 1, mitochondrial	AT2G47510	FUM1	GO:0009651 GO:0006979 GO:0006099 GO:0006108	1,4	EM	9,33 10^{-03}
PSME_00005366-RA	gamma carbonic anhydrase 1, mitochondrial	AT1G47260	GAMMA CA2	GO:0009901 GO:0009853 GO:0009651	0,7	NEC	2,27 10^{-03}
PSME_00041516-RA	GDSL esterase/lipase At4g01130 isoform X1	AT4G01130	AT4G01130.1	GO:0016042	2,6	EM	1,96 10^{-04}
PSME_00021218-RA	glucan endo-1,3-beta-glucosidase 12	AT5G24318			0,1	NEC	1,07 10^{-05}
PSME_00023819-RA	glucan endo-1,3-beta-glucosidase GII-like	AT4G16260	AT4G16260.1	GO:0005975 GO:0009651	0,2	NEC	2,02 10^{-03}
PSME_00019983-RA	glucan endo-1,3-beta-glucosidase, acidic isoform-like	AT4G16260	AT4G16260.1	GO:0009651 GO:0005975	0,1	NEC	5,70 10^{-03}
PSME_00033216-RA	glucose-1-phosphate adenylyltransferase	AT5G48300	ADG1	GO:0005978 GO:0019252	2,5	EM	1,46 10^{-02}
PSME_00009881-RA	glucose-6-phosphate 1-dehydrogenase, cytoplasmic isoform	AT5G40760	G6PD6	GO:0055114 GO:0006006	0,4	NEC	1,48 10^{-02}
PSME_00009882-RA	glucose-6-phosphate 1-dehydrogenase, cytoplasmic isoform	AT5G40760	G6PD6	GO:0055114 GO:0006006	0,3	NEC	8,88 10^{-06}
PSME_00015181-RA	glutamate synthase 1	AT5G53460	GLT1	GO:0042128 GO:0055114 GO:0046686 GO:0006537 GO:0009735	2,5	EM	1,26 10^{-06}
PSME_00010711-RA	glutathione S-transferase F10-like	AT2G30860	GSTF9	GO:0055114 GO:0006952 GO:0006749 GO:0042742 GO:0046686	0,1	NEC	1,99 10^{-05}
PSME_00000864-RA	glutathione S-transferase L3-like isoform X1	AT5G02790	GSTL3	GO:0046686 GO:0006749 GO:0009636	1,6	EM	1,50 10^{-03}
PSME_00031819-RA	glutathione S-transferase omega-like 2	AT4G19880	AT4G19880.2	GO:0046686	0,6	NEC	7,84 10^{-03}
PSME_00032764-RA	glutathione S-transferase U17	AT1G59700	GSTU16	GO:0006749	0,1	NEC	2,05 10^{-04}
PSME_00055362-RA	glutathione S-transferase U17-like	AT1G59700	GSTU16	GO:0006749	4,4	EM	9,62 10^{-05}
PSME_00020299-RA	glutathione S-transferase, amino-terminal domain protein	AT2G30870	GSTF10	GO:0009414 GO:0046686 GO:0006749	0,3	NEC	2,20 10^{-03}
PSME_00032232-RA	glutathione-dependent formaldehyde dehydrogenase	AT5G43940	HOT5	GO:0008219 GO:0055114 GO:0010286 GO:0009611 GO:0048316	0,2	NEC	5,31 10^{-03}
PSME_00044624-RA	glutelin type-A 1-like	AT2G28680	AT2G28680.1		8,3	EM	7,82 10- 10
PSME_00015394-RA	glyceraldehyde-3-phosphate dehydrogenase GAPCP2, chloroplastic	AT1G16300		GO:0006096 GO:0006006 GO:0055114 GO:0080022 GO:0005975	0,8	NEC	1,28 10^{-02}
PSME_00007962-RA	glycerate dehydrogenase isoform X2	AT1G68010	HPR	GO:0055114 GO:0009853	0,7	NEC	1,13 10^{-03}
PSME_00017753-RA	glycerophosphodiester phosphodiesterase GDPDL3 isoform X1	AT5G58170	SVL5	GO:0006071 GO:0006629	2,1	EM	2,24 10^{-05}
PSME_00008228-RA	glycerophosphodiester phosphodiesterase GDPDL6	AT4G26690	SHV3	GO:0006071 GO:0006629	1,8	EM	1,58 10^{-02}
PSME_00021849-RA	glycine-rich protein DOT1	AT1G76010	AT1G76010.1		2,8	EM	8,21 10^{-04}
PSME_00030801-RA	glycine-rich RNA-binding protein GRP1A-like	AT4G39260	CCR1	GO:0009651 GO:0009737 GO:0009735 GO:0009409	1,4	EM	9,06 10^{-03}
PSME_00055366-RA	glycine-rich RNA-binding, abscisic acid-inducible protein isoform X1	AT5G04280	AtRZ-1c	GO:0045892 GO:0009409 GO:0009414	1,5	EM	5,58 10^{-03}
PSME_00028884-RA	heat shock 70 kDa protein 8	AT2G32120	HSP70T-2	GO:0009644 GO:0009408 GO:0042542 GO:0006457	0,3	NEC	2,79 10^{-04}
PSME_00017046-RA	heat shock 70 kDa protein, mitochondrial	AT5G09590	HSP70	GO:0006457 GO:0009651 GO:0009408 GO:0046686	2,4	EM	1,57 10^{-03}
PSME_00002089-RA	heat shock protein 83-like	AT5G52640	HSP90.1	GO:0009408 GO:0042742 GO:0061077	0,3	NEC	1,82 10^{-06}
PSME_00046532-RA	heat shock protein 83-like	AT5G52640	HSP90.1	GO:0042742 GO:0061077 GO:0009408	0,4	NEC	9,33 10^{-05}
PSME_00005303-RA	heat shock protein 90-1	AT2G04030	CR88	GO:0045037 GO:0009414 GO:0009704 GO:0009793 GO:0009408 GO:0009651 GO:0006457	1,9	EM	1,02 10^{-04}

PSME_00005257-RA	heme oxygenase 1, chloroplastic	AT2G26670	TED4	GO:0015979 GO:0006788 GO:0009813 GO:0010119	0,3	NEC	$4,62\ 10^{-03}$
PSME_00001372-RA	heterogeneous nuclear ribonucleoprotein 1-like	AT5G55550	AT5G55550.3		2,6	EM	$1,47\ 10^{-02}$
PSME_00041420-RA	heterogeneous nuclear ribonucleoprotein A2 homolog 1	AT3G13224			1,6	EM	$2,34\ 10^{-03}$
PSME_00008343-RA	heterogeneous nuclear ribonucleoprotein F-like	AT5G66010	AT5G66010.1		1,6	EM	$5,38\ 10^{-03}$
PSME_00001780-RA	high mobility group B protein 1-like	AT3G51880	HMGB1	GO:0006333	1,8	EM	$2,00\ 10^{-03}$
PSME_00011989-RA	histone deacetylase HDT2-like	AT3G44750	HDA3	GO:0006355 GO:0009294 GO:0006351	2,1	EM	$6,87\ 10^{-03}$
PSME_00013947-RA	hsp70-Hsp90 organizing protein 3	AT4G12400	Hop3	GO:0009644 GO:0009408 GO:0010286 GO:0042542	0,6	NEC	$1,89\ 10^{-03}$
PSME_00051470-RA	hydroxyphenylpyruvate reductase-like, partial	AT1G79870	AT1G79870.1	GO:0055114	1,6	EM	$1,07\ 10^{-03}$
PSME_00009048-RA	hypersensitive-induced response protein 1	AT5G62740	HIR1		0,5	NEC	$2,94\ 10^{-02}$
PSME_00033280-RA	importin alpha, putative	AT3G06720	IMPA-1	GO:0006886 GO:0006607 GO:0006606	2,1	EM	$6,27\ 10^{-05}$
PSME_00011005-RA	importin beta-like SAD2	AT2G31660	SAD2	GO:0006606 GO:0007165	1,6	EM	$2,72\ 10^{-04}$
PSME_00034436-RA	inositol-3-phosphate synthase isoform X1	AT2G22240	MIPS2	GO:0006021 GO:0009644 GO:0009408 GO:0009793 GO:0051607 GO:0008654 GO:0042542 GO:0009733 GO:0016036 GO:0050832 GO:0042742	4,9	EM	$2,87\ 10^{-08}$
PSME_00003232-RA	isoflavone reductase-like protein	AT4G39230	AT4G39230.1	GO:0046686	0,5	NEC	$1,41\ 10^{-03}$
PSME_00047891-RA	isopentenyl diphosphate isomerase	AT5G16440	IPP1	GO:0050992 GO:0008299 GO:0015979	0,7	NEC	$9,05\ 10^{-03}$
PSME_00055258-RA	kinesin light chain, putative	AT4G10840	KLCR1		2,0	EM	$2,53\ 10^{-02}$
PSME_00001492-RA	la protein 1	AT4G32720	La1	GO:0042254 GO:0009790	1,3	EM	$2,49\ 10^{-03}$
PSME_00013997-RA	L-ascorbate oxidase homolog	AT4G22010	sks4	GO:0055114	3,0	EM	$1,18\ 10^{-04}$
PSME_00002336-RA	L-ascorbate peroxidase 2	AT1G07890	APX1	GO:0055114 GO:0042744 GO:0006979 GO:0009651 GO:0046686 GO:0009793 GO:0009408 GO:0009735	0,4	NEC	$4,35\ 10^{-07}$
PSME_00033903-RA	L-ascorbate peroxidase, cytosolic	AT1G07890	APX1	GO:0055114 GO:0006979 GO:0009651 GO:0042744 GO:0009735 GO:0009408 GO:0009793 GO:0046686	2,1	EM	$2,74\ 10^{-05}$
PSME_00026301-RA	leucine-rich repeat extensin-like protein 4	AT3G24480	AT3G24480.1	GO:0071555	2,0	EM	$7,44\ 10^{-05}$
PSME_00055770-RA	leucine-rich repeat extensin-like protein 4	AT3G24480	AT3G24480.1	GO:0071555	2,7	EM	$2,79\ 10^{-04}$
PSME_00025010-RA	leucine--tRNA ligase, cytoplasmic	AT1G09620	AT1G09620.1	GO:0006418	1,5	EM	$9,12\ 10^{-03}$
PSME_00008869-RA	linoleate 9S-lipoxygenase 5, chloroplastic-like	AT3G22400	LOX5	GO:0010311 GO:0048364	0,1	NEC	$6,65\ 10^{-03}$
PSME_00039391-RA	lipase-like PAD4	AT3G48080	AT3G48080.1	GO:0006629 GO:0006952	0,5	NEC	$7,12\ 10^{-03}$
PSME_00043872-RA	LOW QUALITY PROTEIN: phospholipase A1-IIdelta	AT2G42690	AT2G42690.1	GO:0006629 GO:0016042	0,1	NEC	$8,44\ 10^{-04}$
PSME_00046745-RA	malate dehydrogenase, mitochondrial-like	AT1G53240	mMDH1	GO:0006108 GO:0005975 GO:0009409 GO:0042742 GO:0009651 GO:0006099 GO:0046686	0,6	NEC	$4,27\ 10^{-04}$
PSME_00007502-RA	mannose-1-phosphate guanyltransferase alpha isoform X1	AT1G74910	AT1G74910.1	GO:0046686 GO:0009058	2,4	EM	$1,46\ 10^{-05}$
PSME_00049017-RA	mannose-1-phosphate guanylyltransferase 1	AT2G39770	CYT1	GO:0030244 GO:0009753 GO:0042742 GO:0009651 GO:0009298 GO:0019853 GO:0009408 GO:0009058	1,7	EM	$1,49\ 10^{-02}$
PSME_00009971-RA	cysteine endopeptidase 1	AT5G50260	CEP1	GO:0009555 GO:0006508 GO:0050832 GO:0051603	2,6	EM	$3,85\ 10^{-03}$
PSME_00031225-RA	mitochondrial aldehyde dehydrogenase family protein	AT1G23800	ALDH2B7	GO:0055114	0,2	NEC	$1,97\ 10^{-03}$
PSME_00019501-RA	mitochondrial outer membrane protein porin of 36 kDa	AT3G01280	VDAC1	GO:0006952 GO:0006820	1,5	EM	$1,38\ 10^{-04}$
PSME_00040188-RA	mitochondrial phosphate carrier protein 3, mitochondrial	AT3G48850	PHT3;2	GO:0009651 GO:0006839 GO:0006810	3,6	EM	$4,15\ 10^{-05}$
PSME_00018930-RA	mitochondrial Rho GTPase 1-like isoform X1	AT5G27540	MIRO1	GO:0007264 GO:0009790 GO:0007005 GO:0009793	1,5	EM	$9,07\ 10^{-03}$
PSME_00019117-RA	molybdate-anion transporter	AT4G27720	AT4G27720.1	GO:0015689	1,5	EM	$1,78\ 10^{-02}$
PSME_00022879-RA	myosin heavy chain, skeletal muscle	AT2G32240	AT2G32240.1	GO:0009737 GO:0046686	2,4	EM	$3,26\ 10^{-03}$
PSME_00001652-RA	NADH dehydrogenase	AT2G20360	AT2G20360.1	GO:0009651 GO:0055114	0,5	NEC	$3,57\ 10^{-03}$
PSME_00026116-RA	NADH dehydrogenase	AT5G08530	CI51	GO:0055114 GO:0006120	0,5	NEC	$6,43\ 10^{-04}$
PSME_00013240-RA	NADP-dependent malic enzyme	AT1G79750	NADP-ME4	GO:0055114 GO:0006108 GO:0006633 GO:0009845 GO:0009793	0,4	NEC	$1,26\ 10^{-04}$

PSME_00006922-RA	N-alpha-acetyltransferase 15, NatA auxiliary subunit-like isoform X2	AT1G80410	EMB2753	GO:0009793 GO:0009414	1,8	EM	$7,80\ 10^{-05}$
PSME_00032826-RA	nitrilase/nitrile hydratase NIT4A-like protein	AT5G22300	NIT4	GO:0006807	0,1	NEC	$1,61\ 10^{-06}$
PSME_00006791-RA	non-symbiotic hemoglobin, putative	AT2G16060	HB1		0,5	NEC	$4,04\ 10^{-03}$
PSME_00054870-RA	not_annotated				0,1	NEC	$2,85\ 10^{-04}$
PSME_00006308-RA	nuclear cap-binding protein subunit 1	AT2G13540	ABH1	GO:0009737 GO:0000184 GO:0006370 GO:0006412 GO:0006406 GO:0000398	1,7	EM	$8,94\ 10^{-04}$
PSME_00026346-RA	Os02g0831900	AT4G23650	CDPK6	GO:0010119 GO:0018105 GO:0035556 GO:0009738 GO:0046777 GO:0006468 GO:0009651	2,6	EM	$1,23\ 10^{-06}$
PSME_00030199-RA	Os06g0602600	AT2G34160	AT2G34160.1		1,8	EM	$1,15\ 10^{-02}$
PSME_00053283-RA	Os06g0726100	AT3G12500	HCHIB	GO:0006032 GO:0016998 GO:0031640 GO:0000272 GO:0050832 GO:0046686	0,3	NEC	$4,58\ 10^{-03}$
PSME_00000323-RA	Os09g0400100	AT4G39330	CAD9	GO:0055114 GO:0009809 GO:0009735	11,6	EM	$8,75\ 10^{-03}$
PSME_00048700-RA	Os12g0468600				0,2	NEC	$1,12\ 10^{-02}$
PSME_00003087-RA	Os12g0623900	AT5G17920	ATMS1	GO:0009086 GO:0009651 GO:0046686 GO:0032259	1,7	EM	$4,26\ 10^{-04}$
PSME_00024602-RA	patellin-3	AT1G30690	AT1G30690.1	GO:0006810 GO:0007049 GO:0051301	1,6	EM	$2,90\ 10^{-04}$
PSME_00050927-RA	pathogenesis-related protein bet V I family protein				0,2	NEC	$4,88\ 10^{-03}$
PSME_00054279-RA	pathogenesis-related protein PR-4-like	AT3G04720	PR4	GO:0031640 GO:0042742 GO:0009723 GO:0009651	0,1	NEC	$7,64\ 10^{-05}$
PSME_00043968-RA	pectinesterase	AT1G53830	PME2	GO:0045490 GO:0042545	0,1	NEC	$1,11\ 10^{-03}$
PSME_00043969-RA	pectinesterase-like	AT1G11580	PMEPCRA	GO:0031640 GO:0042545 GO:0050832 GO:0017148 GO:0045490	0,1	NEC	$3,30\ 10^{-08}$
PSME_00004775-RA	peptide methionine sulfoxide reductase A1-like	AT5G07470	PMSR3	GO:0055114 GO:0006464 GO:0009416 GO:0006979	0,5	NEC	$5,14\ 10^{-04}$
PSME_00037764-RA	peptidyl-prolyl cis-trans isomerase	AT2G16600	ROC3	GO:0000413 GO:0006457 GO:0007165	2,1	EM	$9,62\ 10^{-04}$
PSME_00044050-RA	peptidyl-prolyl cis-trans isomerase	AT2G16600	ROC3	GO:0000413 GO:0007165 GO:0006457	1,4	EM	$1,55\ 10^{-02}$
PSME_00010934-RA	peptidyl-prolyl cis-trans isomerase CYP40	AT2G15790	SQN	GO:0000413 GO:0006457	2,5	EM	$4,44\ 10^{-04}$
PSME_00016025-RA	peroxidase 12	AT1G71695	AT1G71695.1	GO:0055114 GO:0042744 GO:0006979	0,1	NEC	$1,19\ 10^{-02}$
PSME_00016027-RA	peroxidase 12	AT1G71695	AT1G71695.1	GO:0055114 GO:0042744 GO:0006979	0,4	NEC	$4,26\ 10^{-04}$
PSME_00030113-RA	peroxidase 12	AT1G71695	AT1G71695.1	GO:0055114 GO:0006979 GO:0042744	0,2	NEC	$4,13\ 10^{-04}$
PSME_00038261-RA	peroxidase 12	AT1G71695	AT1G71695.1	GO:0055114 GO:0042744 GO:0006979	0,3	NEC	$3,24\ 10^{-06}$
PSME_00048140-RA	peroxidase 12-like	AT1G71695	AT1G71695.1	GO:0055114 GO:0042744 GO:0006979	0,6	NEC	$2,33\ 10^{-02}$
PSME_00038357-RA	peroxidase 55-like	AT4G37530		GO:0042744 GO:0009651 GO:0006979 GO:0055114	0,3	NEC	$3,62\ 10^{-03}$
PSME_00013623-RA	peroxidase 56-like, partial	AT5G05340	PRX52	GO:0009809 GO:0055114 GO:0042744 GO:0006979	0,2	NEC	$1,97\ 10^{-04}$
PSME_00016462-RA	peroxidase 72-like	AT5G66390	PRX72	GO:0042744 GO:0006979 GO:0009809 GO:0055114	0,1	NEC	$8,22\ 10^{-06}$
PSME_00005464-RA	peroxiredoxin-2B-like	AT1G60740	AT1G60740.1	GO:0045454 GO:0055114	1,9	EM	$2,24\ 10^{-03}$
PSME_00012153-RA	peroxisomal acyl-coenzyme A oxidase 1	AT4G16760	ACX1	GO:0009555 GO:0006635 GO:0009620 GO:0009695 GO:0046686 GO:0009611	1,5	EM	$2,85\ 10^{-03}$
PSME_00017712-RA	peroxisomal fatty acid beta-oxidation multifunctional protein AIM1	AT4G29010	AIM1	GO:0009695 GO:0009845 GO:0009908 GO:0007275 GO:0006635	2,7	EM	$3,06\ 10^{-04}$
PSME_00021289-RA	phosphoenolpyruvate carboxylase 4	AT1G68750	PPC4	GO:0015977 GO:0015979 GO:0006099	9,5	EM	$7,02\ 10^{-05}$
PSME_00010128-RA	phosphoethanolamine N-methyltransferase 1 isoform X1	AT1G73600		GO:0032259	4,9	EM	$5,11\ 10^{-05}$
PSME_00019283-RA	phosphoglucomutase, chloroplastic-like isoform X1	AT5G51820	PGM	GO:0005975 GO:0019252 GO:0009409 GO:0005978 GO:0006006	0,4	NEC	$2,34\ 10^{-04}$
PSME_00035611-RA	phosphoglucomutase, putative	AT5G17530	AT5G17530.3	GO:0005975	0,6	NEC	$1,50\ 10^{-02}$
PSME_00020464-RA	phosphoglycerate kinase	AT1G79550	PGK	GO:0009408 GO:0009416 GO:0006096	0,6	NEC	$4,44\ 10^{-03}$
PSME_00020463-RA	phosphoglycerate kinase	AT1G79550	PGK	GO:0006096 GO:0009408 GO:0009416	2,3	EM	$5,90\ 10^{-04}$
PSME_00042401-RA	phosphoglycerate kinase, chloroplastic	AT3G12780	PGK1	GO:0009409 GO:0046686 GO:0019253 GO:0006096	0,5	NEC	$5,73\ 10^{-04}$
PSME_00008521-RA	phosphoinositide phosphatase SAC7 isoform X3	AT3G51460	RHD4	GO:0009611	1,6	EM	$2,03\ 10^{-02}$

PSME_00012089-RA	phospholipase D alpha 1-like	AT3G15730	PLDALPHA1	GO:0046470 GO:0009737 GO:0009845 GO:0046686 GO:0006631 GO:0009738 GO:0016042 GO:0010119	0,3	NEC	$7{,}05\ 10^{-03}$
PSME_00016010-RA	phosphoserine aminotransferase 1, chloroplastic-like	AT4G35630	PSAT1	GO:0009735	0,6	NEC	$7{,}08\ 10^{-04}$
PSME_00053194-RA	phosphoserine aminotransferase 2, chloroplastic-like, partial	AT4G35630	PSAT1	GO:0009735	0,4	NEC	$1{,}01\ 10^{-05}$
PSME_00002972-RA	plasma membrane-associated cation-binding protein 1	AT4G20260	PCAP1	GO:0009414 GO:0009735 GO:0075733 GO:0051716 GO:0009409 GO:0042742	11,5	EM	$1{,}54\ 10^{-02}$
PSME_00014881-RA	plasma membrane-associated cation-binding protein 1	AT4G20260	PCAP1	GO:0009409 GO:0042742 GO:0009414 GO:0009735 GO:0075733 GO:0051716	2,9	EM	$7{,}77\ 10^{-03}$
PSME_00011312-RA	plastidic glucose transporter 4-like	AT5G16150	PGLCT	GO:0046323 GO:0035428	0,4	NEC	$1{,}02\ 10^{-03}$
PSME_00011513-RA	polyadenylate-binding protein 2	AT4G34110	PAB2	GO:0006413 GO:0016032 GO:0009651 GO:0006446 GO:0000184	1,7	EM	$7{,}47\ 10^{-05}$
PSME_00028723-RA	polyadenylate-binding protein 5	AT1G49760	PAB8	GO:0046686 GO:0006417 GO:0016032	0,6	NEC	$1{,}53\ 10^{-02}$
PSME_00003206-RA	pre-mRNA-processing factor 39	AT1G04080	PRP39		1,2	EM	$1{,}87\ 10^{-02}$
PSME_00000080-RA	pre-mRNA-processing protein 40A	AT1G44910	PRP40A	GO:0006355 GO:0008380 GO:0000398 GO:0006351	1,6	EM	$2{,}09\ 10^{-02}$
PSME_00012209-RA	pre-mRNA-processing-splicing factor 8-like	AT1G80070	SUS2	GO:0000398	1,4	EM	$1{,}59\ 10^{-02}$
PSME_00007928-RA	presequence protease 2, chloroplastic/mitochondrial-like isoform X2	AT1G49630	PREP2	GO:0006508 GO:0016485	1,5	EM	$3{,}31\ 10^{-04}$
PSME_00037603-RA	probable ADP-ribosylation factor GTPase-activating protein AGD8	AT4G17890	AGD8		5,9	EM	$2{,}23\ 10^{-04}$
PSME_00000620-RA	probable aldehyde dehydrogenase	AT5G62530	ALDH12A1	GO:0072593 GO:0055114 GO:0010133 GO:0009651	1,5	EM	$4{,}93\ 10^{-03}$
PSME_00017173-RA	probable aldo-keto reductase 1	AT1G60730	AT1G60730.3	GO:0055114	0,6	NEC	$1{,}65\ 10^{-03}$
PSME_00021883-RA	probable aldo-keto reductase 4	AT1G60710	ATB2	GO:0046686 GO:0055114	0,6	NEC	$6{,}92\ 10^{-05}$
PSME_00021353-RA	probable apyrase 1	AT5G18280	APY2	GO:0009846	2,3	EM	$8{,}67\ 10^{-04}$
PSME_00013602-RA	probable chalcone--flavonone isomerase 3	AT5G05270	CHIL	GO:0080167 GO:0009813	0,2	NEC	$1{,}01\ 10^{-02}$
PSME_00054019-RA	probable cinnamyl alcohol dehydrogenase 1	AT1G72680	CAD1	GO:0055114 GO:0009809	0,5	NEC	$9{,}32\ 10^{-03}$
PSME_00021323-RA	probable fructokinase-1	AT3G59480	AT3G59480.1	GO:0019252	3,3	EM	$3{,}72\ 10^{-08}$
PSME_00055205-RA	probable glutathione S-transferase GSTU6-like	AT1G10370	ERD9	GO:0080167 GO:0006749 GO:0009704 GO:0009651	0,4	NEC	$1{,}55\ 10^{-03}$
PSME_00000032-RA	probable GPI-anchored adhesin-like protein PGA55 isoform X2	AT5G53620	AT5G53620.1		2,1	EM	$1{,}01\ 10^{-02}$
PSME_00032525-RA	probable histone-arginine methyltransferase 1.3	AT5G49020	PRMT4A	GO:0006355 GO:0009909 GO:0010228 GO:0006351	1,9	EM	$7{,}87\ 10^{-04}$
PSME_00054694-RA	probable inactive receptor kinase At1g48480	AT3G17840	RLK902	GO:0007169 GO:0006468	3,2	EM	$2{,}30\ 10^{-03}$
PSME_00017674-RA	probable methyltransferase PMT14	AT1G26850	AT1G26850.1	GO:0032259	1,6	EM	$4{,}44\ 10^{-03}$
PSME_00034626-RA	probable methyltransferase PMT17 isoform X1	AT4G10440	AT4G10440.1		2,5	EM	$5{,}85\ 10^{-04}$
PSME_00031559-RA	probable methyltransferase PMT21	AT1G31850	AT1G31850.1		3,3	EM	$5{,}34\ 10^{-07}$
PSME_00011927-RA	probable methyltransferase PMT9 isoform X1	AT3G23300	AT3G23300.1		2,6	EM	$7{,}88\ 10^{-04}$
PSME_00032129-RA	probable mitochondrial-processing peptidase subunit beta	AT3G02090	MPPBETA	GO:0006122 GO:0006508 GO:0016485 GO:0009060	5,1	EM	$5{,}15\ 10^{-04}$
PSME_00015539-RA	probable N-acetyl-gamma-glutamyl-phosphate reductase, chloroplastic	AT2G19940	AT2G19940.1	GO:0006526 GO:0046686 GO:0006520 GO:0055114	0,5	NEC	$1{,}09\ 10^{-02}$
PSME_00008314-RA	probable pectinesterase/pectinesterase inhibitor 51	AT5G09760	AT5G09760.1	GO:0045490 GO:0042545	0,3	NEC	$4{,}50\ 10^{-06}$
PSME_00032134-RA	probable prolyl 4-hydroxylase 6	AT3G28480	AT3G28480.2	GO:0055114	2,2	EM	$1{,}18\ 10^{-03}$
PSME_00046703-RA	probable protein Pop3-like	AT5G22580	AT5G22580.1		0,1	NEC	$7{,}62\ 10^{-03}$
PSME_00031401-RA	probable quinone oxidoreductase	AT5G61510	AT5G61510.1	GO:0030154 GO:0055114 GO:0009644	0,4	NEC	$6{,}73\ 10^{-08}$
PSME_00026241-RA	probable receptor protein kinase TMK1	AT2G01820	AT2G01820.1	GO:0009555	4,6	EM	$6{,}88\ 10^{-06}$
PSME_00018936-RA	probable rhamnose biosynthetic enzyme 1	AT1G63000	NRS/ER	GO:0010253 GO:0019305 GO:0055114 GO:0071555	3,4	EM	$1{,}49\ 10^{-07}$
PSME_00036537-RA	probable ribose-5-phosphate isomerase 3, chloroplastic	AT2G01290	RPI2	GO:0008219 GO:0010228 GO:0009052	0,6	NEC	$2{,}21\ 10^{-02}$

PSME_00035171-RA	probable UMP/CMP kinase 3 isoform X1	AT5G26667	PYR6	GO:0006221	1,6	EM	$1,31\ 10^{-03}$
PSME_00028506-RA	probable xyloglucan glycosyltransferase 5	AT4G31590	CSLC5	GO:0071555	2,4	EM	$1,88\ 10^{-02}$
PSME_00032965-RA	protease Do-like 7 isoform X2	AT3G03380	DEG7	GO:0006508	0,2	NEC	$8,80\ 10^{-08}$
PSME_00003897-RA	protein argonaute 10, partial	AT5G43810	AGO10	GO:0007275 GO:0031047 GO:0006355 GO:0006351 GO:0006417 GO:0051607	3,1	EM	$1,97\ 10^{-03}$
PSME_00031420-RA	protein BONZAI 1-like	AT2G32910	AT2G32910.1		2,0	EM	$2,88\ 10^{-04}$
PSME_00031277-RA	protein CDI	AT1G64980	CDI	GO:0009846	1,7	EM	$4,15\ 10^{-03}$
PSME_00044523-RA	protein COBRA-like isoform X1	AT5G60920	COB	GO:0009651 GO:0016049 GO:0010215	2,8	EM	$4,17\ 10^{-03}$
PSME_00007776-RA	protein disulfide isomerase-like protein	AT5G60640	PDIL1-4	GO:0006979 GO:0045454 GO:0006457	1,6	EM	$3,70\ 10^{-04}$
PSME_00026375-RA	protein disulfide oxidoreductase, putative	AT5G38900	AT5G38900.1		0,2	NEC	$9,65\ 10^{-05}$
PSME_00000513-RA	protein DJ-1 homolog D-like isoform X2	AT3G02720	DJ1D		0,6	NEC	$5,59\ 10^{-03}$
PSME_00039380-RA	protein HOTHEAD-like isoform X1	AT1G12570	f5o11 31	GO:0055114	0,1	NEC	$4,68\ 10^{-03}$
PSME_00025197-RA	protein notum homolog	AT2G46930	PAE3	GO:0071555	0,2	NEC	$2,62\ 10^{-04}$
PSME_00001755-RA	protein RSI-1-like				5,7	EM	$5,41\ 10^{-07}$
PSME_00017679-RA	protein SGT1 homolog	AT4G11260	SGT1B	GO:0006511 GO:0009734 GO:0050832 GO:0030163 GO:0009867 GO:0010187 GO:0009793 GO:0009408	0,7	NEC	$1,\ 10\ 10^{-02}$
PSME_00022313-RA	protein shisa-4-like	AT1G63830	AT1G63830.2		2,0	EM	$1,59\ 10^{-02}$
PSME_00055640-RA	protein TORNADO 2	AT5G46700	TRN2	GO:0009956 GO:0010015	0,1	NEC	$1,94\ 10^{-07}$
PSME_00001066-RA	protein transport protein SEC23	AT3G23660	AT3G23660.2	GO:0006810	1,5	EM	$1,57\ 10^{-03}$
PSME_00002579-RA	protein transport protein sec24	AT2G27460	AT2G27460.1	GO:0016192 GO:0006888 GO:0006886	2,7	EM	$7,12\ 10^{-04}$
PSME_00010258-RA	protein transport protein Sec24-like At3g07100	AT3G07100	ERMO2	GO:0007030 GO:0006810 GO:0016049 GO:0006888 GO:0006886	5,2	EM	$3,02\ 10^{-04}$
PSME_00013998-RA	protein transport protein Sec24-like At4g32640 isoform X2	AT4G32640	AT4G32640.1	GO:0006888 GO:0006886	2,7	EM	$3,52\ 10^{-04}$
PSME_00045047-RA	protein YLS2-like	AT3G51430	YLS2	GO:0009753 GO:0009620 GO:0009058 GO:0009723	0,5	NEC	$6,13\ 10^{-03}$
PSME_00036731-RA	purple acid phosphatase 4-like	AT1G14700	PAP3	GO:0016311	0,2	NEC	$1,91\ 10^{-03}$
PSME_00000594-RA	putative 4-hydroxy-4-methyl-2-oxoglutarate aldolase 2	AT5G16450	AT5G16450.1	GO:0051252	0,3	NEC	$1,12\ 10^{-03}$
PSME_00001217-RA	putative formamidase C869.04 isoform X4	AT4G37560	AT4G37560.1		0,2	NEC	$1,86\ 10^{-03}$
PSME_00036273-RA	putative methylesterase 11, chloroplastic	AT1G69240			2,8	EM	$2,52\ 10^{-03}$
PSME_00012693-RA	putative proline--tRNA ligase C19C7.06	AT3G62120	AT3G62120.1	GO:0006418 GO:0006433	1,7	EM	$1,19\ 10^{-03}$
PSME_00041149-RA	pyrophosphate-energized vacuolar membrane proton pump-like	AT1G15690	AVP1	GO:0048366 GO:0009651 GO:0009414 GO:0015992	2,7	EM	$3,23\ 10^{-07}$
PSME_00016809-RA	pyruvate dehydrogenase E1 component subunit alpha, mitochondrial	AT1G59900	E1 ALPHA	GO:0046686 GO:0006096 GO:0055114	0,7	NEC	$1,04\ 10^{-02}$
PSME_00016703-RA	ran-binding protein 1 homolog b	AT5G58590	RANBP1	GO:0006511 GO:0006405 GO:0000060 GO:0051028	1,7	EM	$6,16\ 10^{-03}$
PSME_00035817-RA	ras GTPase-activating protein-binding protein 1	AT3G25150	AT3G25150.2		3,2	EM	$5,91\ 10^{-05}$
PSME_00006929-RA	ras-related protein RABD1	AT1G02130	RA-5	GO:0046686 GO:0015031 GO:0006888	1,4	EM	$9,29\ 10^{-03}$
PSME_00042189-RA	ras-related protein RABH1e-like	AT5G10260	RABH1e	GO:0015031 GO:0006891 GO:0042147 GO:0006890	1,9	EM	$2,54\ 10^{-03}$
PSME_00009436-RA	receptor-like protein kinase FERONIA	AT3G51550	FER	GO:0048364 GO:0009742 GO:0046777 GO:0006468 GO:0009723 GO:0009791 GO:0050832 GO:0009738	2,0	EM	$6,11\ 10^{-03}$
PSME_00038375-RA	receptor-like protein kinase THESEUS 1	AT5G54380	THE1	GO:0009826 GO:0009742 GO:0046777 GO:0006468 GO:0009791	2,9	EM	$1,20\ 10^{-04}$
PSME_00020246-RA	reticulocalbin-2	AT5G08580	AT5G08580.1		0,5	NEC	$8,19\ 10^{-03}$
PSME_00019330-RA	reticulon-like protein B1	AT4G11220	BTI2		1,5	EM	$2,63\ 10^{-02}$
PSME_00046521-RA	ribonuclease 1-like	AT1G26820	RNS3		3,5	EM	$1,02\ 10^{-03}$
PSME_00029357-RA	RNA-binding protein 24-B	AT1G76460	AT1G76460.1		6,3	EM	$4,44\ 10^{-04}$
PSME_00002235-RA	RNA-binding protein Nova-1 isoform X2	AT5G04430	BTR1L	GO:0008380 GO:0009735	1,4	EM	$5,70\ 10^{-03}$

PSME_00000537-RA	ruBisCO large subunit-binding protein subunit alpha isoform X1	AT2G28000	CPN60A	GO:0009658 GO:0006457 GO:0009790 GO:0007005 GO:0061077 GO:0006458	0,8	NEC	7,12 10^{-03}
PSME_00025101-RA	S-adenosylmethionine synthase 1	AT2G36880	MAT3	GO:0009809 GO:0006556 GO:0006730	2,5	EM	1,86 10^{-06}
PSME_00026577-RA	S-adenosylmethionine synthase 2	AT3G17390	MTO3	GO:0009409 GO:0006555 GO:0009809 GO:0006556 GO:0006730	0,3	NEC	2,46 10^{-03}
PSME_00028718-RA	S-adenosylmethionine synthase 2	AT3G17390	MTO3	GO:0006730 GO:0006556 GO:0006555 GO:0009809 GO:0009409	0,7	NEC	6,87 10^{-03}
PSME_00026579-RA	S-adenosylmethionine synthase 5	AT3G17390	MTO3	GO:0009409 GO:0006555 GO:0009809 GO:0006556 GO:0006730	0,1	NEC	3,88 10^{-04}
PSME_00033735-RA	serine carboxypeptidase-like 27	AT3G07990	SCPL27	GO:0006508 GO:0051603	3,0	EM	1,26 10^{-02}
PSME_00033307-RA	Serine carboxypeptidase-like 35	AT5G08260	scpl35	GO:0051603 GO:0006508	0,3	NEC	7,09 10^{-05}
PSME_00002021-RA	serine carboxypeptidase-like 45	AT1G28110	SCPL45	GO:0006508 GO:0051603	8,4	EM	3,56 10^{-07}
PSME_00033213-RA	serine carboxypeptidase-like 45	AT1G28110	SCPL45	GO:0006508 GO:0051603	3,3	EM	2,66 10^{-06}
PSME_00031997-RA	serine carboxypeptidase-like enzyme	AT5G42240	scpl42	GO:0051603 GO:0006508	5,3	EM	3,50 10^{-05}
PSME_00014571-RA	serine/threonine-protein phosphatase 5	AT2G42810	PP5.2	GO:0046686 GO:0006470	1,4	EM	8,15 10^{-03}
PSME_00007668-RA	S-formylglutathione hydrolase	AT2G41530	SFGH	GO:0046686	0,7	NEC	1,73 10^{-02}
PSME_00010789-RA	signal recognition particle receptor subunit alpha	AT4G30600	AT4G30600.1	GO:0006614 GO:0006605	0,5	NEC	2,78 10^{-04}
PSME_00005435-RA	single-stranded DNA-binding protein WHY1, chloroplastic-like isoform X2	AT1G14410	WHY1	GO:0006351 GO:0006952 GO:0006281 GO:0006355	3,0	EM	1,13 10^{-04}
PSME_00013696-RA	small nuclear ribonucleoprotein Sm D2-like	AT3G62840	AT3G62840.1	GO:0008380	1,3	EM	2,85 10^{-03}
PSME_00028857-RA	Soluble inorganic pyrophosphatase	AT1G01050	PPa1	GO:0019915 GO:0006796	2,0	EM	5,79 10^{-05}
PSME_00015642-RA	staphylococcal nuclease domain-containing protein 1	AT5G07350	Tudor1	GO:0009651 GO:0006402 GO:0046686 GO:0006397 GO:0031047 GO:0009306	1,4	EM	7,08 10^{-03}
PSME_00014875-RA	stress responsive A/B barrel domain protein	AT2G31670	UP3		0,5	NEC	2,30 10^{-04}
PSME_00029325-RA	strictosidine synthase 1-like	AT3G57030	AT3G57030.1	GO:0009058	0,6	NEC	2, 10 10^{-03}
PSME_00005252-RA	subtilisin-like protease	AT5G67360	ARA12	GO:0006508	0,1	NEC	3,99 10^{-04}
PSME_00003877-RA	sucrose synthase 2	AT4G02280	SUS3	GO:0005986 GO:0009414 GO:0005985	0,4	NEC	5,80 10^{-05}
PSME_00003878-RA	sucrose synthase 3-like	AT4G02280	SUS3	GO:0005985 GO:0009414 GO:0005986	0,3	NEC	6,44 10^{-05}
PSME_00022970-RA	superkiller viralicidic activity 2-like 2	AT2G06990	HEN2	GO:0006397 GO:0016070	1,7	EM	1,79 10^{-05}
PSME_00018456-RA	T-complex protein 1 subunit beta	AT5G20890	AT5G20890.1		1,3	EM	1,12 10^{-02}
PSME_00016087-RA	thaumatin-like protein	AT1G18250	ATLP-1	GO:0051707	0,2	NEC	5,79 10^{-03}
PSME_00001915-RA	thiol protease aleurain-like	AT3G45310	AT3G45310.1	GO:0006508	7,0	EM	2,17 10^{-05}
PSME_00014373-RA	thiol protease aleurain-like	AT3G45310	AT3G45310.1	GO:0006508	2,0	EM	1, 10 10^{-02}
PSME_00042511-RA	Thioredoxin	AT1G11530	CXXS1	GO:0045454	0,1	NEC	4,62 10^{-03}
PSME_00050044-RA	thioredoxin H4-1	AT3G08710	TH9	GO:0055114 GO:0045454 GO:0007154	2,2	EM	7,22 10^{-04}
PSME_00049348-RA	TolB protein-related isoform 2, partial	AT1G21670	AT1G21670.1		0,3	NEC	1,89 10^{-02}
PSME_00037425-RA	transaldolase-like protein	AT5G13420	TRA2	GO:0006098 GO:0005975 GO:0009809 GO:0046686	0,2	NEC	3,30 10^{-08}
PSME_00042167-RA	transketolase, chloroplastic	AT2G45290	TKL2	GO:0046686	0,4	NEC	3,28 10^{-06}
PSME_00040482-RA	transmembrane 9 superfamily member 2	AT2G01970	AT2G01970.1	GO:0006810	1,6	EM	1,89 10^{-04}
PSME_00006153-RA	transmembrane 9 superfamily member 4-like	AT3G13772	TMN7	GO:0006811	2,0	EM	1,47 10^{-06}
PSME_00007345-RA	transmembrane 9 superfamily member 7	AT3G13772	TMN7	GO:0006811	4,3	EM	5,12 10^{-09}
PSME_00033866-RA	transmembrane nine 7 isoform 1	AT3G13772	TMN7	GO:0006811	1,7	EM	3,28 10^{-03}
PSME_00011992-RA	transmembrane protein, putative	AT3G12650	AT3G12650.1		0,5	NEC	3,27 10^{-03}
PSME_00025791-RA	triosephosphate isomerase, chloroplastic	AT2G21170	TIM	GO:0006094 GO:0080022 GO:0009658 GO:0006096 GO:0019253	0,5	NEC	1,31 10^{-04}
PSME_00048393-RA	tRNA pseudouridine synthase A, mitochondrial-like	AT1G20370	AT1G20370.1		3,5	EM	2,05 10^{-05}
PSME_00048275-RA	tropinone reductase-like 3	AT4G05530	IBR1	GO:0006631 GO:0055114	1,3	EM	2,78 10^{-02}
PSME_00001420-RA	tryptophan--tRNA ligase, cytoplasmic	AT3G04600	AT3G04600.1	GO:0006418	1,4	EM	2,59 10^{-04}
PSME_00010571-RA	tubulin alpha chain	AT1G50010	TUA2	GO:0007017 GO:0009651	1,5	EM	9,97 10^{-03}

PSME_00048529-RA	tubulin alpha chain	AT1G50010	TUA2	GO:0007017 GO:0009651	2,2	EM	$3,81\ 10^{-03}$
PSME_00045437-RA	tubulin alpha-3 chain	AT5G19780	TUA5	GO:0007017 GO:0046686	0,5	NEC	$7,29\ 10^{-03}$
PSME_00045033-RA	tubulin alpha-3 chain-like	AT5G19780	TUA5	GO:0046686 GO:0007017	0,5	NEC	$2,77\ 10^{-04}$
PSME_00022639-RA	tubulin beta-1 chain	AT5G12250	TUB6	GO:0009651 GO:0007017 GO:0009409	2,0	EM	$2,80\ 10^{-04}$
PSME_00037947-RA	two-on-two hemoglobin-3	AT4G32690	GLB3	GO:0009733 GO:0015671	3,5	EM	$6,36\ 10^{-03}$
PSME_00013135-RA	U1 small nuclear ribonucleoprotein A-like	AT1G76940	AT1G76940.1		1,5	EM	$1,42\ 10^{-02}$
PSME_00016277-RA	ubiquinol oxidase 1a, mitochondrial-like	AT3G22370	AOX1A	GO:0009409 GO:0055114	4,6	EM	$1,72\ 10^{-06}$
PSME_00009309-RA	ubiquitin-conjugating enzyme E2 7	AT3G46460	UBC13	GO:0006511 GO:0000209	3,0	EM	$2,21\ 10^{-03}$
PSME_00009333-RA	UBP1-associated protein 2B-like	AT2G41060	AT2G41060.1	GO:0008219 GO:0006952	1,9	EM	$1,69\ 10^{-04}$
PSME_00024734-RA	UDP-arabinopyranose mutase 1	AT3G02230	RGP1	GO:0009651 GO:0009832 GO:0009555 GO:0071555 GO:0033356 GO:0030244	5,3	EM	$7,83\ 10^{-09}$
PSME_00032374-RA	UDP-D-apiose/UDP-D-xylose synthase 2	AT1G08200		GO:0009226 GO:0071555	1,5	EM	$2,49\ 10^{-03}$
PSME_00028107-RA	UDP-glucose 6-dehydrogenase 4	AT3G29360	UGD2	GO:0005975 GO:0055114 GO:0006024	2,2	EM	$1,93\ 10^{-06}$
PSME_00003124-RA	UDP-glucose:glycoprotein glucosyltransferase	AT1G71220		GO:0006486 GO:0018279	1,4	EM	$1,19\ 10^{-02}$
PSME_00000157-RA	UDP-glucuronate 4-epimerase 3	AT1G02000	GAE2	GO:0005975	1,8	EM	$3,37\ 10^{-04}$
PSME_00031028-RA	UDP-glucuronic acid decarboxylase 2-like	AT3G62830		GO:0019305	1,8	EM	$4,42\ 10^{-04}$
PSME_00022060-RA	UDP-glucuronic acid decarboxylase 6-like	AT2G28760	UXS6		2,5	EM	$2,49\ 10^{-03}$
PSME_00024031-RA	UF642 l-GalL-responsive protein 1	AT5G25460	DGR2	GO:0010015 GO:0080167	5,6	EM	$1,46\ 10^{-08}$
PSME_00038002-RA	UF642 l-GalL-responsive protein 1	AT5G25460	DGR2	GO:0010015 GO:0080167	4,2	EM	$1,90\ 10^{-05}$
PSME_00026496-RA	Uncharacterized protein isoform 1	AT5G13500	AT5G13500.1		0,2	NEC	$1,26\ 10^{-02}$
PSME_00046317-RA	Uncharacterized protein isoform 1	AT1G07040	AT1G07040.1		0,1	NEC	$5,45\ 10^{-03}$
PSME_00000641-RA	Uncharacterized protein L484_007981	AT3G20790	AT3G20790.1	GO:0055114	1,5	EM	$1,90\ 10^{-02}$
PSME_00026338-RA	Uncharacterized protein TCM_006340	AT3G49720	CGR2	GO:0040007 GO:0045489	2,3	EM	$1,60\ 10^{-05}$
PSME_00049835-RA	uninformative				5,3	EM	$2,69\ 10^{-07}$
PSME_00031292-RA	vacuolar amino acid transporter 1	AT2G39130	AT2G39130.1		28,2	EM	$1,99\ 10^{-06}$
PSME_00000072-RA	very-long-chain (3R)-3-hydroxyacyl-CoA dehydratase PASTICCINO 2A	AT5G10480	PAS2	GO:0007275 GO:0030154 GO:0051302	0,7	NEC	$6,21\ 10^{-03}$
PSME_00016527-RA	villin-2	AT2G41740	VLN2	GO:0007010 GO:0051017	1,9	EM	$6,21\ 10^{-05}$
PSME_00000168-RA	villin-3	AT3G57410	VLN3	GO:0051017 GO:0007010	0,5	NEC	$1,02\ 10^{-02}$
PSME_00015064-RA	V-type proton ATPase 16 kDa proteolipid subunit-like	AT1G19910		GO:0015991	2,8	EM	$1,34\ 10^{-06}$
PSME_00004177-RA	V-type proton ATPase catalytic subunit A	AT1G78900	VHA-A	GO:0015992 GO:0015991 GO:0009555 GO:0009651 GO:0046034 GO:0007030	1,4	EM	$1,42\ 10^{-03}$
PSME_00002523-RA	V-type proton ATPase subunit a3	AT4G39080	VHA-A3	GO:0070072 GO:0015986 GO:0015991	2,0	EM	$3,20\ 10^{-05}$
PSME_00004839-RA	V-type proton ATPase subunit C	AT1G12840	DET3	GO:0009809 GO:0015991 GO:0009826	1,8	EM	$1,90\ 10^{-04}$
PSME_00001707-RA	V-type proton ATPase subunit d2	AT3G28715	AT3G28715.1	GO:0015991 GO:0015992	1,9	EM	$1,97\ 10^{-03}$
PSME_00023262-RA	V-type proton ATPase subunit E	AT4G11150	TUF	GO:0009409 GO:0042742 GO:0015991 GO:0009735 GO:0009832 GO:0009793 GO:0009651 GO:0015986 GO:0007030	1,4	EM	$9,52\ 10^{-03}$
PSME_00015864-RA	YTH domain-containing family protein 1 isoform X1	AT3G13460	ECT2		2,3	EM	$9,38\ 10^{-03}$
PSME_00011787-RA	YTH domain-containing family protein 2	AT1G48110	ECT7		1,9	EM	$2,67\ 10^{-04}$
PSME_00014883-RA	YTH domain-containing family protein 2	AT1G48110	ECT7		1,9	EM	$5,66\ 10^{-03}$
PSME_00034990-RA	zinc finger CCCH domain-containing protein 14-like	AT5G06770	AT5G06770.1	GO:0006355	3,0	EM	$5,07\ 10^{-04}$
