# Supplementary material for: Cytological, Biochemical and Molecular Events of the Embryogenic State in Douglas-fir (Pseudotsuga menziesii [Mirb.])
Source: Front Plant Sci. 2019 Feb 28;10:118. doi: 10.3389/fpls.2019.00118 (PMC6403139; doi:10.3389/fpls.2019.00118)
Supplement: Supplementary file 15 [file Table_5.pdf]

Supplementary Table S5. Functional classification according to gene ontology (GO) of proteins that were significantly differentially expressed between isogenic embryonal mass (EM) and non-embryogenic callus (NEC) of all three Douglas-fir genotypes. Total number of proteins annotated to each GO term indicated in the first column and corresponding percentage in the second column.

	EM		NEC	
	**No**	**%**	**No**	**%**
**Biological regulation (GO:0065007)**	**7**	**2.0**	**5**	**2.5**
**Cellular component organization or biogenesis (GO:0071840)**	**24**	**6.9**	**8**	**4.0**
**Cellular process (GO:0009987)**	**83**	**24.0**	**33**	**16.7**
Cellular component movement (GO:0006928)	*4*	*1.2*	*2*	*1.0*
Cytokinesis (GO:0000910)	*2*	*0.6*	*0*	*0.0*
Cell cycle (GO:0007049)	*7*	*2.0*	*3*	*1.5*
Chromosome segregation (GO:0007059)	*4*	*1.2*	*2*	*1.0*
Cell communication (GO:0007154)	*4*	*1.2*	*3*	*1.5*
**Developmental process (GO:0032502)**	**3**	**0.9**	**3**	**1.5**
**Localization (GO:0051179)**	**34**	**9.4**	**5**	**2.5**
Protein localization (GO:0008104)	*4*	*1.2*	*0*	*0.0*
Transport (GO:0006810)	*34*	*9.8*	*5*	*2.5*
**Metabolic process (GO:0008152)**	**106**	**30.6**	**72**	**36.4**
Phosphate-containing compound metabolic process (GO:0006796)	*24*	*6.9*	*2*	*1.0*
Biosynthetic process (GO:0009058)	*21*	*6.1*	*13*	*6.6*
Vitamin metabolic process (GO:0006766)	*0*	*0.0*	*3*	*1.5*
Primary metabolic process (GO:0044238)	*102*	*29.5*	*59*	*29.8*
Catabolic process (GO:0009056)	*26*	*7.5*	*11*	*5.6*
Sulfur compound metabolic process (GO:0006790)	*6*	*1.7*	*8*	*4.0*
Coenzyme metabolic process (GO:0006732)	*1*	*0.3*	*6*	*3.0*
Nitrogen compound metabolic process (GO:0006807)	*36*	*10.4*	*14*	*7.1*
Generation of precursor metabolites and energy (GO:0006091)	*5*	*1.4*	*12*	*6.1*
Secondary metabolic process (GO:0019748)	*1*	*0.3*	*3*	*1.5*
**Response to stimulus (GO:0050896)**	**8**	**2.3**	**9**	**4.5**
Response to stress (GO:0006950)	*6*	*1.7*	*8*	*4.0*
response to abiotic stimulus (GO:0009628)	*1*	*0.3*	*0*	*0.0*
Response to endogenous stimulus (GO:0009719)	*1*	*0.3*	*0*	*0.0*
Immune response (GO:0006955)	*1*	*0.3*	*0*	*0.0*
**Immune system process (GO:0002376)**	**1**	**0.3**	**0**	**0.0**
**Unclassified**	**80**	**23.1**	**63**	**31.8**
